# Notch signaling regulates metabolic heterogeneity in glioblastoma stem cells

**DOI:** 10.18632/oncotarget.18117

**Published:** 2017-05-23

**Authors:** N. Sumru Bayin, Joshua D. Frenster, Rajeev Sen, Sheng Si, Aram S. Modrek, Nataliya Galifianakis, Igor Dolgalev, Valerio Ortenzi, Irineu Illa-Bochaca, Anadjeet Khahera, Jonathan Serrano, Luis Chiriboga, David Zagzag, John G. Golfinos, Werner Doyle, Aristotelis Tsirigos, Adriana Heguy, Mitch Chesler, Mary Helen Barcellos-Hoff, Matija Snuderl, Dimitris G. Placantonakis

**Affiliations:** ^1^ Department of Neurosurgery, NYU School of Medicine, New York, NY, USA; ^2^ Kimmel Center for Stem Cell Biology, NYU School of Medicine, New York, NY, USA; ^3^ Neuroscience Institute, NYU School of Medicine, New York, NY, USA; ^4^ Genome Technology Center, NYU School of Medicine, New York, NY, USA; ^5^ Department of Pathology, NYU School of Medicine, New York, NY, USA; ^6^ Department of Medicine, NYU School of Medicine, New York, NY, USA; ^7^ Department of Radiation Oncology, NYU School of Medicine, New York, NY, USA; ^8^ Perlmutter Cancer Center, NYU School of Medicine, New York, NY, USA; ^9^ Brain Tumor Center, NYU School of Medicine, New York, NY, USA; ^10^ Applied Bioinformatics Laboratories, Office of Science & Research, NYU School of Medicine, New York, NY, USA; ^11^ Developmental Biology Program, Sloan Kettering Institute, New York, NY, USA; ^12^ Department of Medicine, Washington University School of Medicine, St Louis, MO, USA; ^13^ Department of Radiation Oncology, University of California, San Francisco, CA, USA

**Keywords:** tumor metabolism, glioblastoma stem cells, Notch signaling, CD133, tumor vasculature

## Abstract

Glioblastoma (GBM) stem cells (GSCs) reside in both hypoxic and vascular microenvironments within tumors. The molecular mechanisms that allow GSCs to occupy such contrasting niches are not understood. We used patient-derived GBM cultures to identify GSC subtypes with differential activation of Notch signaling, which co-exist in tumors but occupy distinct niches and match their metabolism accordingly. Multipotent GSCs with Notch pathway activation reside in perivascular niches, and are unable to entrain anaerobic glycolysis during hypoxia. In contrast, most CD133-expressing GSCs do not depend on canonical Notch signaling, populate tumors regardless of local vascularity and selectively utilize anaerobic glycolysis to expand in hypoxia. Ectopic activation of Notch signaling in CD133-expressing GSCs is sufficient to suppress anaerobic glycolysis and resistance to hypoxia. These findings demonstrate a novel role for Notch signaling in regulating GSC metabolism and suggest intratumoral GSC heterogeneity ensures metabolic adaptations to support tumor growth in diverse tumor microenvironments.

## INTRODUCTION

Glioblastoma (GBM) is the most common primary brain malignancy (http://www.cbtrus.org). Despite surgery and chemoradiotherapy, prognosis remains poor [1-3]. GBM growth is maintained by a dynamic cellular hierarchy dominated by GBM stem cells (GSCs) [4-10]. Due to their inherent resistance to current therapies, GSCs represent crucial therapeutic targets [9-11].

A major challenge in GBM is its inter- [12, 13] and intra-tumoral [14-16] heterogeneity. In particular, GBM’s microenvironmental heterogeneity is highlighted by areas of microvascular proliferation interspersed with hypoxic regions of pseudopalisading necrosis (PPN) [17]. Both microenvironments harbor GSCs [18-27]. The differences in vascularity and oxygen tension between these niches suggest distinct molecular mechanisms regulating GSC self-renewal and metabolism. However, these mechanisms are not understood.

The Notch signaling pathway, critical for neural stem cell (NSC) self-renewal and differentiation [28-31], also regulates GSC self-renewal, tumorigenicity, radioresistance and differentiation into vascular lineages [6, 32-36]. Upon binding the Delta or Jagged families of ligands, the Notch receptor is cleaved by γ-secretase [31]. Cleaved Notch intracellular domain (NICD) translocates to the nucleus, where it forms a complex with RBPJ and MAML to activate transcription of *HES* and *HEY* genes, which maintain multipotency [29-31]. Tumor endothelium provides Notch ligands, which support GSC self-renewal in the perivascular niche [18, 37-40], similar to NSCs [41-43]. In light of the theory that avascular microenvironments lacking endothelium-derived Notch ligands also harbor GSCs, we hypothesized that the Notch pathway is required for self-renewal of perivascular GSCs, but not GSCs located in hypoxic niches.

Here, we show that nuclear NICD is found in tumor cells within perivascular but not hypoxic regions of human GBM. In contrast, CD133 (PROM1), a transmembrane glycoprotein that marks GSCs [4, 9, 19, 20, 44, 45], is expressed primarily in areas of PPN and less so in perivascular niches. Prospective isolation of cells with active Notch signaling (Notch^hi^) using patient-derived cultures bearing a fluorescent Notch reporter demonstrates only partial overlap and extensive segregation of Notch^hi^ cells and CD133-expressing (CD133^hi^) GSC populations *in vitro* and *in vivo* in intracranial xenograft tumors.. These cell populations occupy discreet niches in tumor xenografts, similar to patient tumors, and demonstrate distinct metabolic, transcriptional and differentiation profiles. Notch^hi^ cells not only reside in perivascular niches, but also contribute pericyte lineages to their vascular microenvironment *via* a broader multipotency profile compared to CD133^hi^ cells. We demonstrate that Notch^hi^ cells are vulnerable to hypoxia due to inability to entrain anaerobic glycolysis, as opposed to CD133^hi^ cells. Ectopic activation of Notch signaling in CD133^hi^ cells is sufficient to confer vulnerability to hypoxia and reprogram metabolism away from anaerobic glycolysis.

Our findings indicate Notch signaling is heterogeneously activated within the GSC population and regulates metabolic adaptations to the local microenvironment. Our model provides a mechanistic understanding of intratumoral GSC heterogeneity, as well as a platform for elucidating microenvironmental regulation of stem cell behavior.

## RESULTS

### Notch signaling is activated in perivascular niches but not hypoxic areas of human GBM

**Figure 1:**
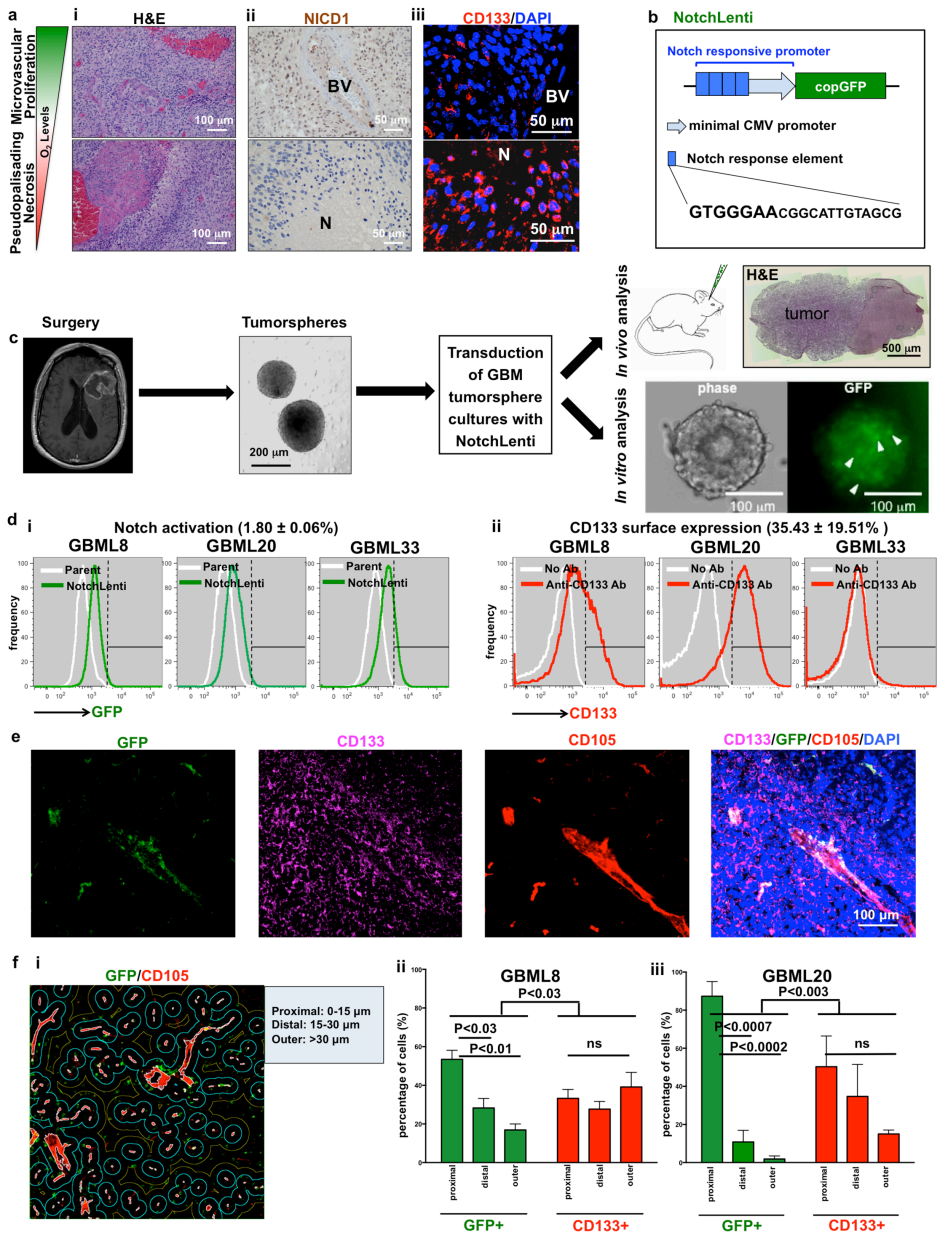
Notch activation and CD133 cell surface expression show differential intratumoral localization in human GBM **a.**, i. H&E reveals areas of microvascular proliferation (top panel) and PPN (bottom panel) within the same human GBM biospecimen. **a.**, ii. Nuclear NICD1 immunoreactivity is seen in perivascular areas but not in PPN regions. **a.**, iii. In contrast, CD133 immunoreactivity is seen in both microenvironments. **b.** Schematic of the lentiviral vector used to monitor Notch pathway activation. The 20 bp-long Notch response element contains a consensus GTGGGAA site found in Notch transcriptional targets. **c.** Schematic depicting the approach for obtaining patient-derived primary tumorsphere cultures and orthotopic tumor xenografts after transduction with NotchLenti. Scattered Notch-activated (GFP+) cells (arrowheads) were observed in tumorspheres *in vitro*. **d.** Flow cytometry histograms of GBM lines show gradients of (i) increased GFP fluorescence after transduction with NotchLenti and (ii) CD133 immunoreactivity after incubation with anti-CD133 antibody (*n* = 3 primary cultures). **e.** Immunofluorescent analysis of xenograft tumors generated by GBML8 cells modified with NotchLenti reveals perivascular GFP staining for Notch activation. **f.** Quantification of the distance of GFP+ or CD133-expressing cells from the vasculature (i) in xenograft tumors generated with 2 patient-derived cultures already modified with NotchLenti shows that GFP+ cells prefer a perivascular localization. (*n* = 3 animals for each condition, ii: GBML8: ANOVA, F_(2,8)_ = 16.93, *P* < 0.003 and iii: GBML20: ANOVA, F_(2,8)_ = 6.049, P < 0.03). H&E: hematoxylin and eosin; N: necrosis; BV: Blood vessel; CMV: Cytomegalovirus; GFP: Green Fluorescence Protein; CD105: Endoglin; DAPI: nuclear counter stain; ns: not significant.

To determine the spatial profile of Notch pathway activation in GBM, we stained 9 formalin-fixed paraffin-embedded (FFPE) human GBM biospecimens for Notch1 Intracellular Domain (NICD1) (Supplementary Table 1). All 9 biospecimens were classified as GBM based on H&E staining (Figure 1ai). Nuclear NICD1 was detected in perivascular areas (Figure 1aii, top panel), but was absent in regions of PPN (4/9 biospecimens with areas of PPN; 5 biospecimens did not show PPN) (Figure 1aii, bottom panel; Supplementary Figure 1a).

Immunofluorescence analysis indicated CD133 localizes to both perivascular and avascular areas in human GBM, as in previous reports [19, 20] (Figure 1aiii). Unlike NICD1, CD133 was consistently found in PPN areas in each biospecimen (Supplementary Figure 1b). Given that Notch signaling and CD133 expression showed differential localization and both have been linked with GSCs, we hypothesized expression of these markers identifies distinct GSC populations.

### Prospective isolation of tumor cells with Notch pathway activation

In order to further characterize cell populations identified by activation of Notch signaling and expression of CD133, we used patient-derived tumorsphere cultures (GBML8, 20, 33, 61) [7, 44, 46, 47]. Molecular subtyping of parental biospecimens was performed with DNA methylation profiling (Illumina 450K arrays) (Supplementary Figure 2ai) [47, 48]. The analysis classified GBML8 in the mesenchymal, and GBML20, GBML33 and GBML61 in the RTK1 (proneural) subtypes (Supplementary Figure 2b). Parental tumors had several molecular hallmarks of primary GBM, including trisomy 7 and loss of PTEN, CDKN2A, NF1 and RB1. Tumors showed microvascular proliferation and PPN. In addition, tumors were negative for IDH1/2 mutations (Supplementary Figure 2ai-2aiii).

In these patient-derived cultures, we prospectively isolated cells with active canonical Notch signaling using a lentiviral reporter (NotchLenti) that expresses copGFP, a green fluorescent protein with short half-life (∼2 hours) [49] that allows dynamic visualization of cells upon activation of the Notch pathway (Figure 1b) [29, 30]. The reporter consists of 4 tandem Notch response elements attached to a minimal CMV promoter. Each response element contains a consensus GTGGGAA motif found in the promoters of the NICD target genes *HES1* and *HES5* (Figure 1b). After treatment with 1 μg/ml puromycin to select for transduced cells, we observed scattered GFP+ cells in our tumorsphere cultures (Figure 1c). These cultures were used for further characterization (Figure 1c). Flow cytometric analysis of 3 primary cultures transduced with NotchLenti (GBML8, GBML20, GBML33) showed a gradient of GFP signal (Figure 1d). This flow cytometric profile was similar to the one we obtained with a HES5-mCherry reporter utilizing 760 bp of the *HES5* promoter (not shown) [29]. To define cells with GFP intensities higher than the negative control (parental cultures not transduced with NotchLenti), we used conventional gating with linear cutoffs in fluorescence intensity. We found that only a small population of cells (1.80 ± 0.06%) showed the highest activation of Notch signaling (Figure 1di). CD133 surface expression in these primary cultures was also represented by a gradient with variable percentages of CD133-positive cells (35.43 ± 19.51%) (Figure 1dii).

To test the fidelity of the Notch reporter, we examined the effects of soluble Notch ligand delta-like 4 (Dll4) or the γ-secretase inhibitor DAPT on the abundance of GFP+ cells, using flow cytometry. We observed significant (1.9 ± 0.3-fold) upregulation in the percentage of GFP+ cells upon Dll4 (100 ng/ml) treatment (Supplementary Figure 2a). Conversely, we found significant downregulation in the fraction of GFP+ cells (0.5 ± 0.1-fold) after DAPT (10 μM) treatment (Supplementary Figure 3a). *In vitro* lentiviral overexpression of NICD1 (NICD-OE) increased the mean intensity of GFP fluorescence in primary cultures (Supplementary Figure 3bi) and the abundance of GFP+ cells (Supplementary Figure 3bii). These findings suggested that the NotchLenti construct faithfully reports Notch pathway activation in GBM cells.

We used confocal microscopy to examine the distribution of CD133+ and GFP+ (active Notch signaling) cells in orthotopic tumor xenografts generated from two primary GBM cultures (Figure 1e-1f). While GFP+ cells localized to perivascular spaces, CD133+ cells showed a diffuse distribution within tumors. Using quantitative image analysis, we analyzed the prevalence of CD133+ and GFP+ cells in zones defined according to the distance from the tumor endothelium (Figure 1fi). This analysis revealed that GFP+ cells had a significantly higher probability of residing close to endothelium than CD133+ cells, which had a more uniform spatial distribution (GBML8 and GBML20, Figure 1fii-iii). Collectively, these findings reproduce observations in patients’ tumors and suggest the CD133+ and GFP+ populations are distinct and occupy different niches.

### Notch activation and CD133 surface expression mark distinct cell populations

**Figure 2:**
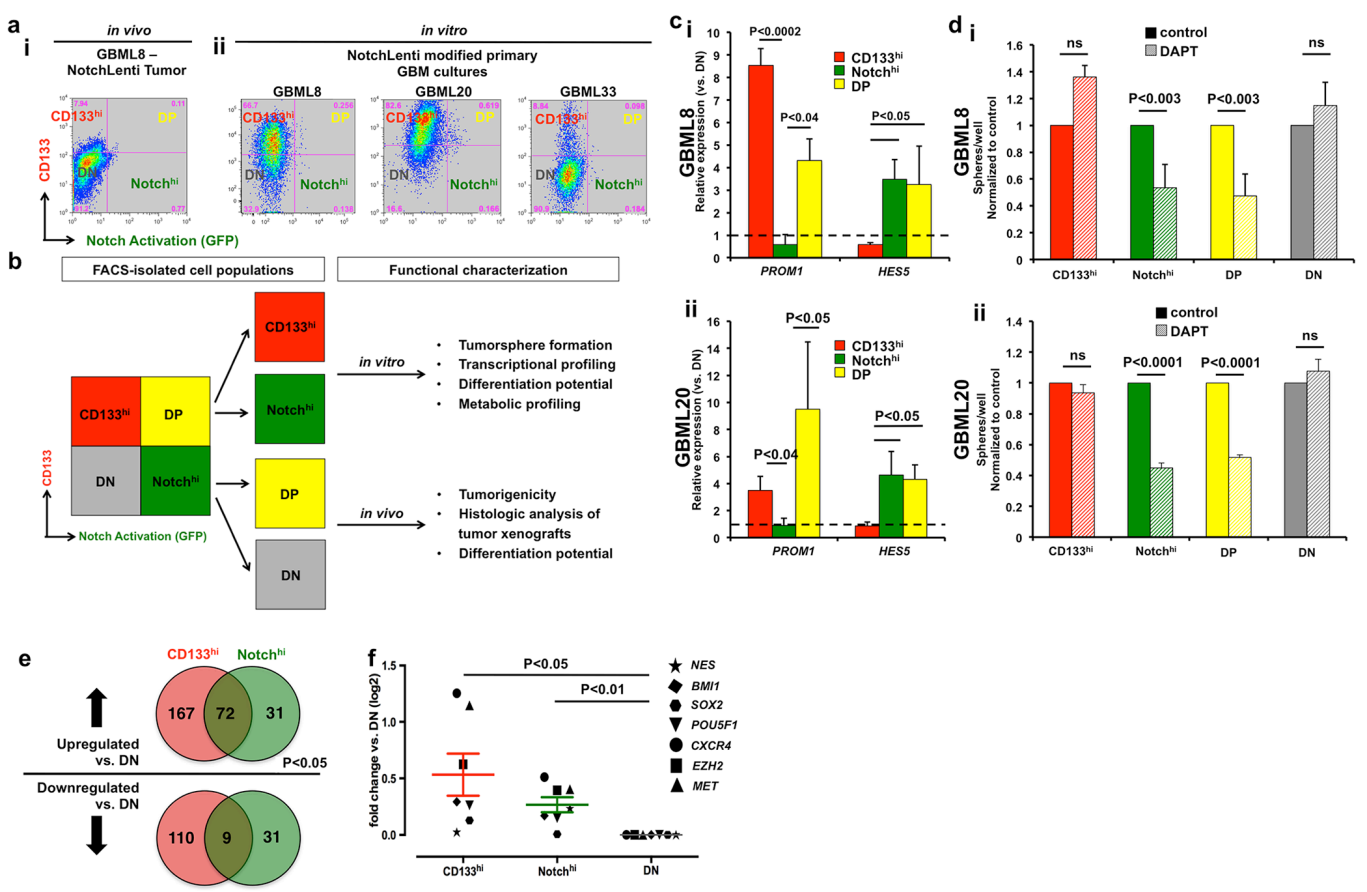
Segregation of Notch^hi^ and CD133^hi^ cell populations **a.**, i Flow cytometric analysis of an intracranial tumor xenograft derived from GBML8-NotchLenti cells shows the segregation of CD133 expression (CD133^hi^) and Notch activation (Notch^hi^). **a.**, ii Flow cytometric analysis of cell surface CD133 and Notch activation (GFP reporter) in 3 primary cultures *in vitro* shows that there is only partial overlap between the two markers. For the analysis in **a.**, GFP gates were drawn based on parental cultures not transduced with NotchLenti, and CD133 gates were based on control conditions without antibody (as shown in Supplementary Figure 2c). **b.** Experimental plan for studying Notch^hi^ and CD133^hi^ cells. **c.** FACS-sorted CD133^hi^, Notch^hi^ and DP cells were compared to DN cells for *CD133* (*PROM1*) and *HES5* mRNA expression by qPCR. The experiment was performed on two different primary cultures transduced with NotchLenti: (i) GBML8, and (ii) GBML20 (*n* = 3 FACS experiments/primary cell line, *t*-tests, *P* < 0.05). **d.** After FACS isolation, cells were seeded at low density (10 cells/μl) and tumorsphere formation was analyzed 7 days after isolation. Only Notch-activated populations (Notch^hi^ and DP) showed decreased tumorsphere formation upon pharmacological inhibition of the Notch pathway with 10 µM DAPT in 2 cultures: (i) GBML8: ANOVA, F_(7,14)_ = 9.472, *P* < 0.0002, (ii) GBML20: ANOVA, F_(1.4)_ = 74.98, *P* < 0.001) (*n* = 3 experiments/primary cell line). **e.** To further confirm CD133^hi^ and Notch^hi^ cells represent distinct populations in GBM, we performed RNA-seq from FACS isolated cells from GBML8 and GBML20. RNA sequencing revealed 420 genes that were differentially expressed in CD133^hi^ and Notch^hi^ GSCs compared to DN cells (*P* < 0.05). **f.** When the RNA-seq data were analyzed for known GSC markers, CD133^hi^ and Notch^hi^ cells showed enrichment for these transcripts compared to the DN population (*n* = 7 genes, ANOVA F_(1, 6)_ = 7.490, *P* < 0.03). ns: not significant.

To test the hypothesis that cells with active Notch signaling are distinct from CD133-expressing cells, we performed flow cytometric analysis of NotchLenti-transduced GBM tumorsphere cultures (GBML8, GBML20 and GBML33) and tumor xenografts from these cells. We found *in vivo* segregation of the CD133-expressing (CD133^hi^) cell population (17.55 ± 2.50% of all tumor cells) and the population of cells with active Notch signaling (Notch^hi^; 3.87 ± 0.85% of all tumor cells, *n* = 4 mice implanted with NotchLenti-transduced GBML8 cells) (Figure 2ai). *In vitro* flow cytometric analysis of 3 primary cultures transduced with NotchLenti reproduced the segregation observed between Notch^hi^ and CD133^hi^ cells (Figure 2aii, Supplementary Figure 3c). There was only partial overlap between the Notch^hi^ and CD133^hi^ populations, so that only 43.64 ± 25.38% of Notch^hi^ cells were CD133^hi^. Conversely, only 2.4 ± 0.3% of CD133^hi^ cells were Notch^hi^. Supplementary Figure 3cii summarizes the cumulative statistics of all the subpopulations generated with respect to CD133 and Notch activation status: CD133^hi^/Notch^lo^ (CD133^hi^), Notch^hi^/CD133^lo^ (Notch^hi^), double positives (DP) and double negatives (DN). These findings suggested partial overlap between these two markers and largely distinct CD133^hi^ and Notch^hi^ cell populations.

To validate the segregation of CD133^hi^ and Notch^hi^ cell populations at the molecular level and to assess their cellular properties, we FACS-isolated CD133^hi^, Notch^hi^, DP and DN cells, and subjected them to further characterization (Figure 2b). In order to ensure that our results were not confounded by loss of the NotchLenti reporter construct, we performed PCR for the copGFP transgene from genomic DNA isolated from all 4 FACS-sorted cell populations, which confirmed that the reporter transgene was present in the genome of all subpopulations (GBML8, Supplementary Figure 4a).

We performed qRT-PCR for transcripts encoding *HES5*, the principal transcriptional target of the Notch pathway in GBM [50], as well as the *PROM1* (*CD133*) transcript, in FACS-isolated subpopulations. We observed significant enrichment of *PROM1* transcript in CD133^hi^ and DP populations, and *HES5* transcript in Notch^hi^ and DP populations, when compared to DN cells (GBML8: Figure 2ci, GBML20: Figure 2cii), providing validation of our experimental model. Expression of other Notch pathway elements, including *NOTCH1, NOTCH2*, *HES1, HEY 1*, *DLL1*, *JAG1* and *JAG2* showed no significant changes in expression levels among the 4 subpopulations (Supplementary Figure 4b, 4c).

We next examined the ability of these cell populations to initiate tumorspheres *in vitro* and tumor xenografts *in vivo*. First, we FACS-isolated the subpopulations (CD133^hi^, Notch^hi^, DP and DN) and plated them in limiting dilutions (10 cells/μL) to determine sphere formation efficiency. All 4 populations showed equivalent sphere formation ability in 2 primary cultures tested (GBML8: Supplementary Figure 5ai, GBML20: Supplementary Figure 5aii). There were no differences in the size of the spheres (not shown). To test *in vivo* tumorigenicity, we injected FACS-isolated cells into the cerebrum of NOD.SCID mice (1.5 × 10^4^ cells/animal). We observed tumor formation in all conditions (*n* = 2 primary GBM cultures, Supplementary Figure 5b, 5d), suggesting the presence of tumor-initiating cells in all subpopulations. Notably however, while the size of Notch^hi^ cell-derived tumors was not different from that of CD133^hi^ tumors, it was significantly larger than the size of DP and DN-derived tumors (Supplementary Figure 5c), suggesting enhanced tumorigenic potential in Notch^hi^ and CD133^hi^ cells compared to DP and DN cells. CD133^hi^ and Notch^hi^ cells produced tumor xenografts even when injected at even greater dilutions (1.5 × 10^3^ cells/animal; Supplementary Figure 5d).

In addition, we tested the self-renewal capacity of the distinct populations. Serial tumorsphere assay using FACS-isolated subpopulations from GBML8 revealed that all populations maintain their self-renewal for at least 3 passages (Supplementary Figure 5e). To test serial xenograft formation, we FACS-isolated subpopulations from a xenograft tumor formed from GBML20 cells. We observed that all of the animals injected with CD133^hi^ and Notch^hi^ cells showed tumor formation, however only 1/2 animals showed tumor formation from DN cells (*n* = 3 for CD133^hi^ and Notch^hi^ and *n* = 2 for DN) (Supplementary Figure 5f). DP cells were not used for this assay. These findings indicate that CD133^hi^ and Notch^hi^ cells have enhanced and sustained tumorigenicity. Of note, the percentage of CD133^hi^ and Notch^hi^ cells remained stable over a 15-month period *in vitro*, indicating long-term self-renewal (*n* = 3 cultures, Supplementary Figure 5g-5h).

Previous data suggested inhibition of Notch signaling depletes CD133-expressing cells [6, 33]. However, recent reports indicated that not all CD133-positive GBM cells are sensitive to γ-secretase inhibitors [51]. To explain this controversy, we tested whether inhibition of Notch signaling with the γ-secretase inhibitor DAPT (10 µM) differentially impairs tumorsphere formation in these subpopulations. Inhibition of Notch activation selectively inhibited tumorsphere formation in Notch^hi^ and DP, but not CD133^hi^ or DN, cells (GBML8: Figure 2di, GBML20: Figure 2dii). This result suggests tumorsphere initiation by CD133^hi^ cells does not depend on Notch signaling.

### Notch^hi^ and CD133^hi^ have distinct transcriptional profiles but overlapping GSC signatures

To understand the molecular etiology of biological differences between Notch^hi^ and CD133^hi^ GSCs, we performed RNA-seq on FACS-isolated cells from GBML8 and GBML20. We identified 420 genes that were differentially expressed in Notch^hi^ and CD133^hi^ GSCs relative to DN cells (*P* < 0.05; Figure 2e, Supplementary Tables 2-7). Supervised hierarchical clustering based on these 420 genes (Supplementary Figure 6a) revealed cells of the same GSC subtype from different cultures clustered together, suggesting preserved transcriptional signatures of these subtypes across tumors. Unsupervised hierarchical clustering using the entire transcriptome produced similar results (Supplementary Figure 6b). Furthermore, Notch^hi^ and CD133^hi^ cells clustered more closely to each other than to DN cells, possibly due to shared GSC transcripts underrepresented in DN cells. Indeed, when we interrogated RNA-seq data for transcripts associated with GSCs such as *NES*, *SOX2*, *BMI1*, *POU5F1*, *CXCR4*, *EZH2* and *MET* [5, 52], we found CD133^hi^ and Notch^hi^ cells upregulate this gene network relative to the DN population (Figure 2f). These findings suggest CD133^hi^ and Notch^hi^ possess stem-like properties but differ in other aspects of their biology.

We next tested whether molecular signatures of parental tumors, as defined by TCGA data analysis [12], were detected in the 4 cell populations (CD133^hi^, Notch^hi^, DP and DN). Supervised hierarchical clustering with 448 genes used to subtype TCGA biospecimens in Verhaak et al. [12] (Supplementary Figure 6c) validated that the TCGA gene expression signature of parental tumors is preserved within the 4 subpopulations of each tumor (Supplementary Figure 6d). This finding suggested that GSC subtypes did not represent genomically distinct subclones within each tumor, but rather functionally specialized subpopulations. Furthermore, it supports the hypothesis that within any given GBM tumor, regardless of its TCGA molecular subgroup, there exist GSC subtypes with defined and differentially regulated gene networks.

As shown in Supplementary Figure 4b, 4c, CD133^hi^ and Notch^hi^ cells express similar levels of *NOTCH1* and *NOTCH2* transcripts using qRT-PCR assays. This finding was reproduced with RNA-seq data. Furthermore, RNA-seq did not show any difference between the two cell types in transcripts encoding components of the γ-secretase complex (*PSEN1*, *APH1*, *PSENEN*, *NCSTN*) or NICD’s nuclear binding partners *RBPJ* and *MAML1-3*. Overall, these findings suggest that the lack of Notch pathway activation in CD133^hi^ cells is not due to low expression of the basic molecular machinery required for Notch signaling.

### Notch^hi^ GSCs have broader differentiation potential than CD133^hi^ GSCs

To test the cells’ differentiation potential, we examined the progeny of the 4 FACS-sorted populations 7 days after isolation (GBML8 and GBML20, Supplementary Figure 6a-6c). While populations with active Notch signaling (Notch^hi^ and DP) gave rise to both Notch^hi^ and Notch^lo^ populations *in vitro*, CD133^hi^ cells were only able to differentiate into Notch^lo^ progeny (CD133^hi^ and DN cells). DN cells generated CD133^hi^ lineages but failed to produce Notch^hi^ progeny (Supplementary Figure 7a-7c). These results suggest that Notch^hi^ cells have broader differentiation potential than CD133^hi^ cells, which show a restricted differentiation program that excludes lineages with active Notch signaling *in vitro*.

**Figure 3:**
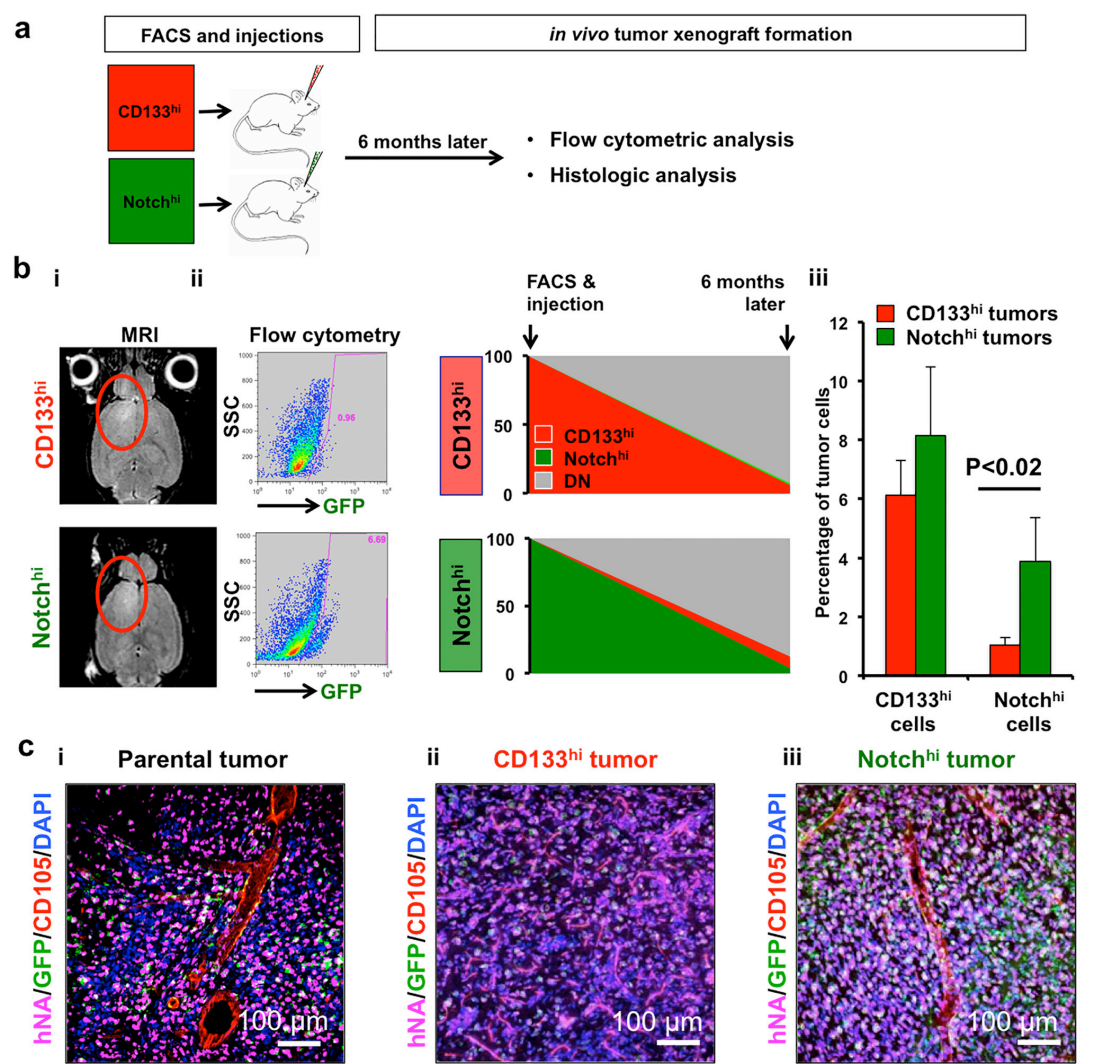
Notch^hi^ cells reside higher in the cellular hierarchy *in vivo* **a.** Cells were isolated with FACS and xenograft tumors generated were analyzed by flow cytometry and histology 6 months after injection. **b.**, i Tumor cells were isolated from intracranial xenografts generated either by CD133^hi^ or Notch^hi^ cells (GBML8) after confirmation of tumors with MRI. **b.**, ii-iii Flow cytometric analysis revealed that tumors initiated by CD133^hi^ GSCs contained significantly lower numbers of Notch^hi^ cells, whereas CD133 percentages did not significantly change between the two different types of tumors (*n* = 3 animals/condition, ANOVA F_(1,8)_ = 9.7, *P* < 0.01). **c.** Confocal immunofluorescence analysis of tumor xenografts generated by parental (i), CD133^hi^ (ii) and Notch^hi^ (iii) GBML8 cells shows that tumors generated by Notch^hi^ GSCs more closely resemble parental tumors than CD133^hi^ tumors (*n* = 3 animals/condition).

To identify progeny of CD133^hi^ and Notch^hi^ GSCs *in vivo*, we injected FACS-sorted GBML8 cells into mouse brains and analyzed tumor xenografts with flow cytometry 6 months later (Figure 3a). After confirmation of tumors with MRI (Figure 3bi), we observed that tumors initiated by Notch^hi^ cells contained all lineages, while tumors initiated by CD133^hi^ GSCs were inefficient in generating Notch^hi^ cells. The abundance of CD133^hi^ cells in the two types of tumor was not different (Figure 3bii-3biii). These findings indicated broader differentiation potential in Notch^hi^ cells and suggested the Notch^hi^ to CD133^hi^ differentiation program is tightly regulated and predominantly unidirectional.

To test the hypothesis that Notch^hi^ cells reside higher than CD133^hi^ cells in GBM’s cellular hierarchy by virtue of their broader differentiation potential, we examined the histology of tumor xenografts obtained by either cell type and compared them to parental tumors derived from unsorted cultures (GBML8, Figure 3ci-3ciii). Tumors generated by Notch^hi^ GSCs (Figure 3ciii) showed elaborate vascular trees, similar to xenografts initiated by injection of parental cultures (Figure 3ci). In contrast, tumors initiated by CD133^hi^ gave rise to a rather monotonous histology, characterized by a uniform network of small vessels (Figure 3cii). This finding supported the hypothesis that Notch^hi^ GSCs have a broader differentiation potential than CD133^hi^ GSCs.

### Notch^hi^ and CD133^hi^ cells differ in their angiogenic potential

**Figure 4:**
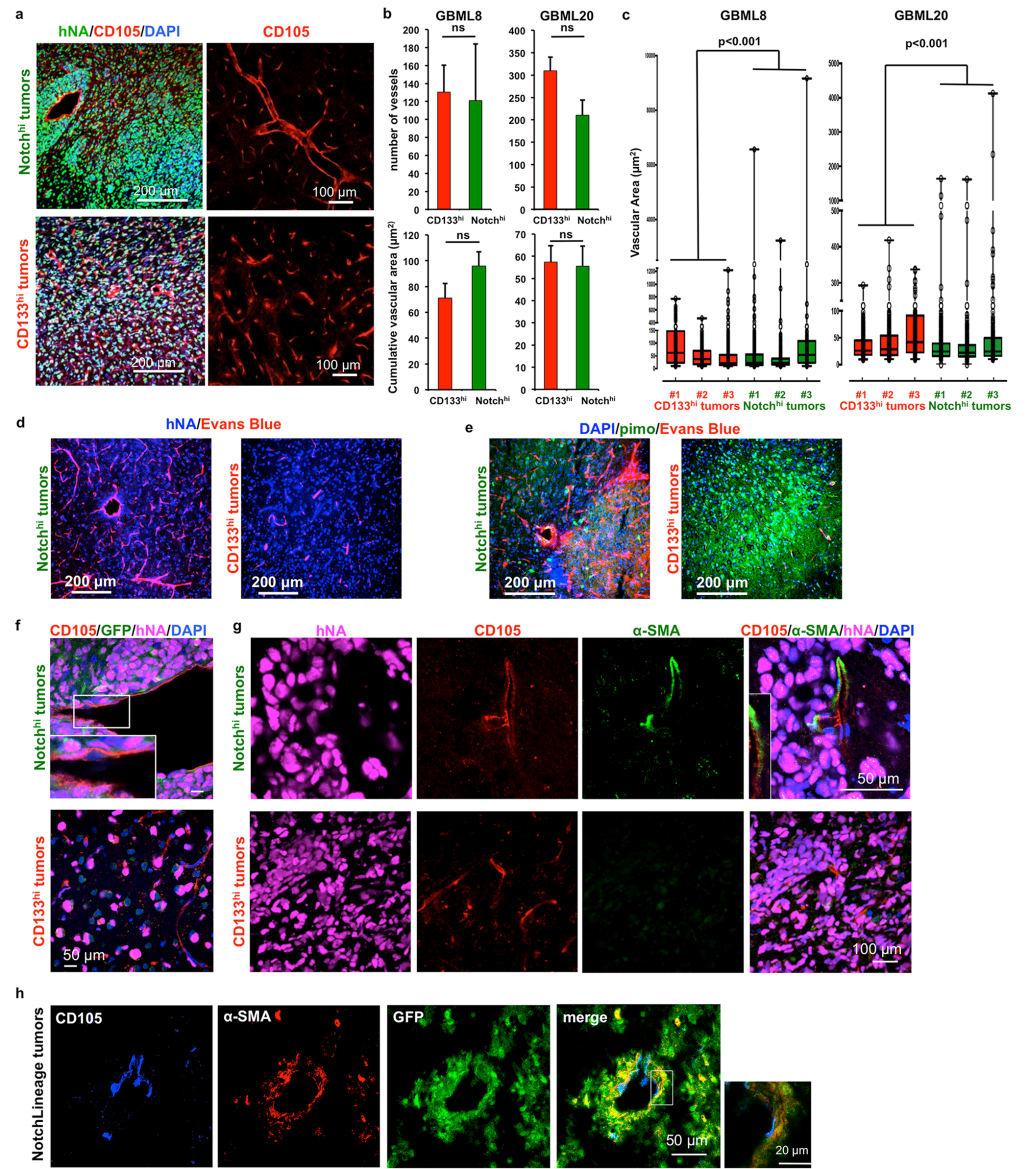
CD133^hi^ and Notch^hi^ GSCs have distinct angiogenic properties **a.** Representative images of GBML8 xenografts show the contrast in vascular morphology of tumors derived from Notch^hi^ (top panel) and CD133^hi^ (bottom panel) cells (*n* = 3 animals/cell type). **b.** No significant change was observed in the number of vessels and average vascular area in xenografts generated by Notch^hi^ or CD133^hi^ cells from cultures GBML8 and GBML20 (4 20x fields/condition, *n* = 3 animals/cell type, *t*-tests, *P* > 0.05). **c.** Notch^hi^-initiated tumors contain large-caliber vessels, which are absent in CD133^hi^ tumors (GBML8 and GBML20, *n* = 3 animals/cell type; Wilcoxon test, *P* < 0.001). **d.**,**e.** CD133^hi^ tumors showed reduced perfusion (Evans Blue staining) and increased hypoxia (pimonidazole staining), compared to tumors generated by Notch^hi^ cells (representative images from GBML8 xenografts, *n* = 3 animals/condition). **f.** Tumors initiated by Notch^hi^ and CD133^hi^ cells do not contain hNA+ endothelium (CD105+ cells) (representative images from GBML8 xenografts, *n* = 3 animals/cell type). **g.** α-SMA+ pericytes envelope larger vessels in Notch^hi^ tumors (top panel). CD133^hi^ tumors are devoid of pericytes (representative images from GBML8 xenografts, *n* = 3 animals/cell type). **h.** Lineage tracing in GBML8 xenografts shows GFP+ pericytes, indicating that they are derived from Notch^hi^ cells. The inset shows that CD105+ endothelium is GFP- (*n* = 3 animals). ns: not significant.

The vascular phenotype of the xenografts initiated by Notch^hi^ and CD133^hi^ cells was distinct. In Notch^hi^-derived tumors, we observed a combination of large and small diameter vessels, whereas CD133^hi^-initiated tumors were devoid of large-diameter vessels and contained a uniform network of smaller vessels (GBML8: Figure 4a, GBML20: Supplementary Figure 8a). Although the number of vessels and cumulative vascular area did not differ between the two conditions (Figure 4b), a dot plot of the area of individual vessels demonstrated a clear difference in size distribution (Figure 4c).

We then tested whether the differences in the morphology of the tumor vasculature correlated with changes in tissue oxygenation. Immunofluorescence analysis revealed that tumors initiated by CD133^hi^ cells, but not Notch^hi^ cells, expressed the hypoxia marker Carbonic Anhydrase 9 (CAIX) (Supplementary Figure 8b) [53]. We observed that, when animals were injected with pimonidazole i.p and Evans Blue i.v. before sacrificing, to analyze vascular perfusion and the extent of hypoxia in these tumors [47, 54], Notch^hi^-initiated tumors showed elevated perfusion, while CD133^hi^-initiated tumors showed less perfusion and increased hypoxia (Figure 4d, 4e). Collectively, these findings indicate that CD133^hi^ cells give rise to hypo-perfusing and hypoxic tumors, in contrast to Notch^hi^-initiated tumors.

To understand the differences in vascular morphology, we analyzed the extent of pericytic and endothelial differentiation, since previous reports indicated GSCs generate endothelial and pericytic lineages [32, 34, 35]. Using confocal microscopy and immunostaining for human nuclear antigen (hNA) and CD105, we were unable to observe tumor-derived endothelial cells in either Notch^hi^ or CD133^hi^ tumors (Figure 4f). This finding suggested that the distinct angiogenic profiles of Notch^hi^ and CD133^hi^ GSCs are unlikely to be due to differences in endothelial differentiation.

The importance of Notch signaling in the generation of vascular pericytes has been previously demonstrated in GBM [32, 55]. To test whether Notch^hi^ GSCs differentiate into pericytes, we compared tumor xenografts initiated by Notch^hi^ and CD133^hi^ GSCs for pericyte immunoreactivity. We found pericytes, identified by α-smooth muscle actin (α-SMA) staining, only in Notch^hi^ tumors, where they surrounded larger-caliber vessels (Figure 4g, top panel). We observed no pericyte staining in CD133^hi^ tumors (Figure 4g, bottom panel). Quantitatively, 49.11 ± 6.56% of α-SMA+ pericytes in Notch^hi^ tumors (GBML8) were tumor-derived, as determined by their co-staining for hNA, similar to ratios in previous literature [32]. These findings suggested that Notch^hi^ GSCs may generate pericyte lineages *in vivo*, which, correlates with formation of large-caliber tumor vessels.

To test the hypothesis that pericytes represent progeny of Notch^hi^ GSCs, we used a tamoxifen-inducible lineage tracing system (NotchLineage) in GBML8, where CreER^T2^ expression is driven by the same Notch-responsive promoter we used for our NotchLenti reporter constructs (Supplementary Figure 8ci) [56]. In this system, induction with tamoxifen causes CreER^T2^, which is specifically expressed in Notch^hi^ GSCs, to catalyze recombination of the reporter construct, leading to deletion of DsRed and expression of GFP in progeny of Notch^hi^ GSCs. One month after tamoxifen induction, we found clones of GFP+ cells within DsRed+ tumor xenografts (Supplementary Figure 8cii). Confocal microscopic analysis of GFP+ cells indicated that the pericyte marker α-SMA overlapped with GFP expression, whereas endothelial cells were GFP- (Figure 4h).

These results suggest Notch^hi^ cells are multipotent and give rise to tumor pericytes, as well as CD133^hi^ lineages. In contrast, CD133^hi^ cells have a restricted differentiation program and do not generate Notch^hi^ cells or pericytes. Tumors initiated by CD133^hi^ cells do not exhibit the complex vascular pattern that characterizes parental and Notch^hi^ GSC-derived tumors. The pericytic transdifferentiation of the Notch^hi^ cells correlates with increased tumor vascularity, perfusion and oxygenation, consistent with previous reports [55].

### CD133^hi^ GSCs expand in hypoxia by selective utilization of anaerobic glycolysis

**Figure 5:**
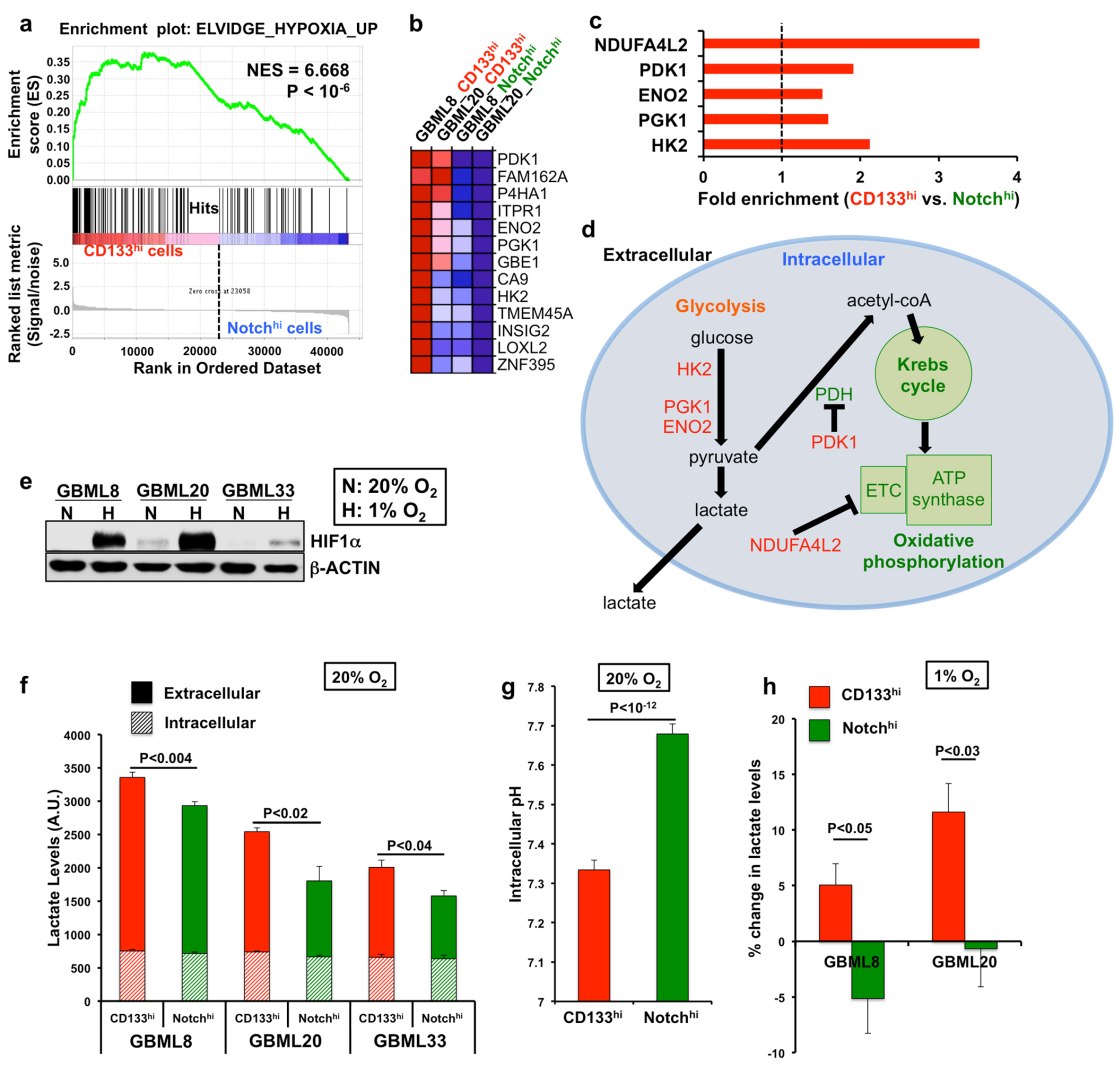
CD133^hi^ GSCs selectively utilize anaerobic glycolysis **a.** GSEA analysis of transcriptomes of CD133^hi^ and Notch^hi^ cells revealed enrichment of hypoxia-responsive genes in CD133^hi^ GSCs. **b.** Heatmap showing differentially expressed genes between CD133^hi^ and Notch^hi^ cells from the same gene set as in (a). **c.** Fold enrichment of critical hypoxia-induced genes in the CD133^hi^ population. **d.** Schematic representation of these genes in the glycolytic and oxidative phosphorylation pathways. **e.** Western blotting for HIF1α shows increased protein levels after 24 hours of hypoxia in 3 different GBM cultures. **f.** CD133^hi^ GSCs had higher total lactate levels than Notch^hi^ GSCs in normoxic conditions (*n* = 3 experiments/primary cell line, *t*-tests, *P* < 0.04). **g.** CD133^hi^ cells had significantly lower intracellular pH compared to Notch^hi^ cells in normoxia (GBML8, *t*-test, *P* < 10^-12^). **h**. After 24 hours of hypoxia, CD133^hi^ cells were able to further increase lactate production compared to Notch^hi^ cells (*n* = 3 experiments, GBML8: *t*-test, *P* < 0.05, GBML20: *t*-test, *P* < 0.03). NES: normalized enrichment score; ETC: electron transport chain.

Our observations raise the possibility that CD133^hi^ cells and Notch^hi^ cells may entrain distinct metabolic adaptations to their local microenvironments. To identify transcriptional networks unique to CD133^hi^ and Notch^hi^ cells that may underlie metabolic differences, we performed unbiased Gene Set Enrichment Analysis (GSEA) [57] on the complete gene lists in the 2 cell types. Gene sets upregulated by hypoxia were significantly enriched in CD133^hi^ cells (Figure 5a, 5b; Supplementary Table 8). This set of genes included the key glycolytic enzymes hexokinase 2 (HK2), phosphoglycerate kinase (PGK1), and enolase 2 (ENO2); but also pyruvate dehydrogenase kinase (PDK1) and NADH dehydrogenase 1 alpha subcomplex subunit 4 like 2 (NDUFA4L2), which suppress oxidative phosphorylation (Figure 5c, 5d) [58-61]. Similarly, analysis of genes differentially expressed between CD133^hi^ and Notch^hi^ cells (*P* < 0.1) (Supplementary Table S9) with GSEA also showed enrichment of hypoxia-related gene sets (Supplementary Table 10a, 10b). Furthermore, analysis of these differentially expressed genes with DAVID [62] revealed that 7 out of the top 10 gene ontology (GO) terms were related to glycolysis and cellular metabolism (*P* < 0.01, Table S8b). These genes were differentially expressed despite CD133^hi^ and Notch^hi^ cells having been cultured under identical normoxic (20% O_2_) conditions prior to RNA isolation.

The localization of CD133^hi^ cells in hypoxic microenvironments, increased hypoxia and decreased perfusion in CD133^hi^-initiated tumors, and the upregulation of a hypoxic transcriptional profile suggested that CD133^hi^ cells preferentially utilize anaerobic glycolysis (Figure 5d). To test this hypothesis, we measured lactate levels in FACS-isolated CD133^hi^ and Notch^hi^ cells in normoxic *vs*. hypoxic conditions. We used western blotting against HIF1α to show stabilization of the protein upon hypoxia, confirming that the low oxygen treatment was effective (Figure 5e). In normoxia, CD133^hi^ cells had 27.5 ± 7.6% higher lactate levels when compared to Notch^hi^ cells (Figure 5f). CD133^hi^ cells also showed significantly lower intracellular pH than Notch^hi^ cells [63], consistent with acidification related to lactate production (Figure 5g). When we subjected CD133^hi^ and Notch^hi^ cells to hypoxia (1% O_2_) for 24 hours, CD133^hi^ cells specifically turned on anaerobic glycolysis even more, manifested as increased lactate levels, as opposed to Notch^hi^ cells, which failed to do so (Figure 5h).

**Figure 6:**
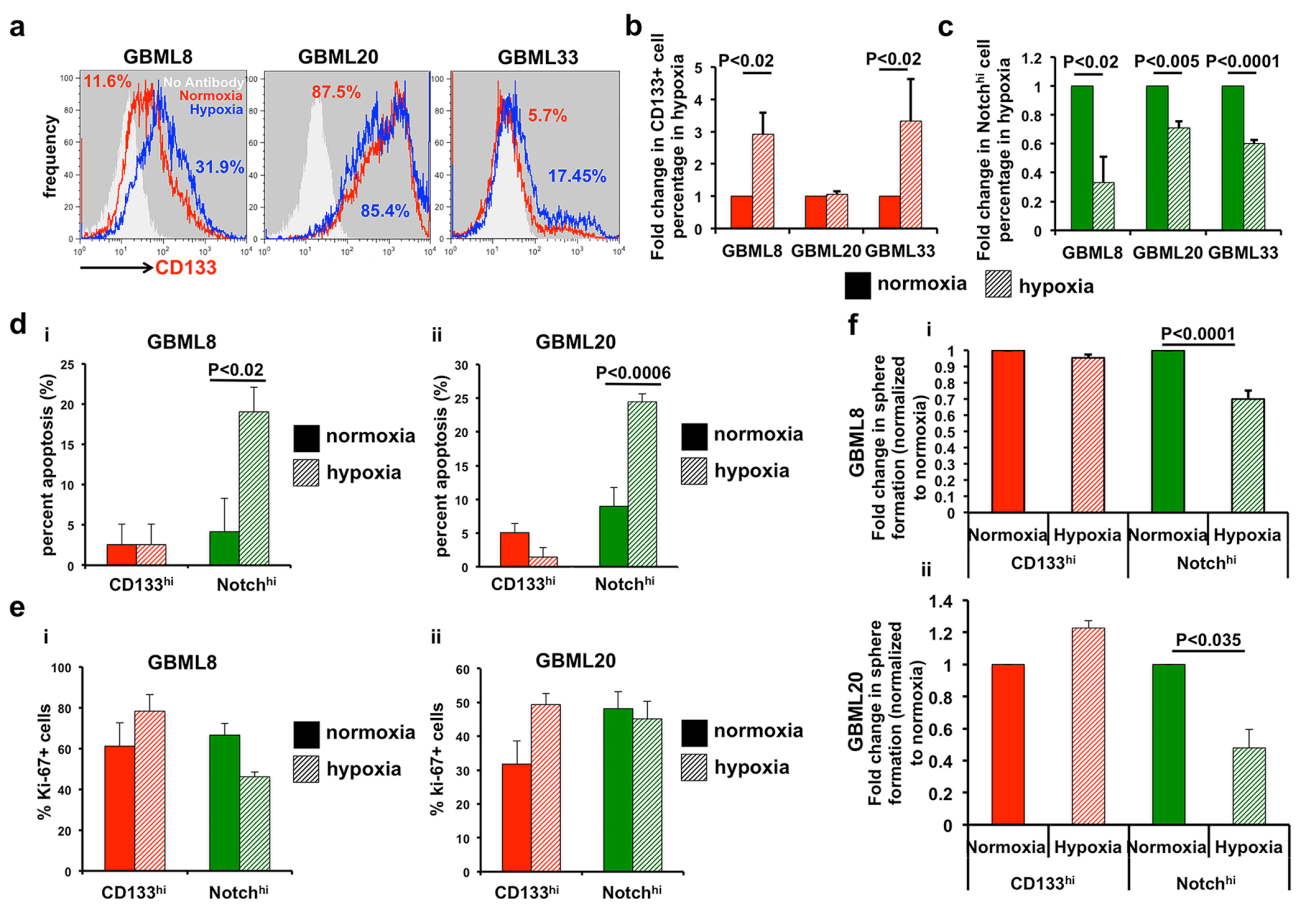
CD133^hi^ GSCs expand in hypoxic conditions at the expense of Notch^hi^ GSCs **a.**, **b.** Flow cytometry shows increased abundance of CD133^h^i cells after 24 hours of hypoxia in 2 of 3 cultures (GBML8 and GBML33: *t*-test, *P* < 0.02). **c.** The abundance of Notch^hi^ GSCs significantly decreases in the same conditions (*t*-test, *P* < 0.02, *n* = 3 primary cultures). **d.** Percent of cells in apoptosis analyzed by TUNEL assay: (i) GBML8: ANOVA, F_(1,8)_ = 5.57, *P* < 0.05; (ii) GBML20: ANOVA, F_(1,8)_ = 10.79, *P* < 0.01. **e.** Percent Ki67+ cells in CD133^hi^ and Notch^hi^ GSCs from two different GBM cultures after 24 hours of hypoxia: (i) GBML8: ANOVA, F_(1,8)_ = 0.047, *P* > 0.05; (ii) GBML20: ANOVA, F_(1,8)_ = 1.9, *P* > 0.05). **f.** FACS-isolated CD133^hi^ and Notch^hi^ cells were allowed to form tumorspheres after 24 hours of hypoxia. Tumorsphere formation was analyzed 7 days later. Notch^hi^ GSCs showed a significant reduction in their tumorsphere formation ability after hypoxia, whereas no significant change was observed in CD133^hi^ GSCs: (i) GBML8: ANOVA, F_(1,8)_ = 20.27, *P* < 0.002; (ii) GBML20: ANOVA, F_(1,8)_ = 56.10, *P* < 0.018.

To test whether the ability of CD133^hi^ cells to utilize anaerobic glycolysis bestowed upon them a selective growth advantage at the expense of Notch^hi^ cells, we subjected primary cultures modified with the NotchLenti reporter to hypoxia for 24 hours. We observed that the percentage of CD133^hi^ cells increased significantly in 2/3 cultures (Figure 6a,b), consistent with prior observations [24]. GBML20, which did not show a significant increase, already had very high levels of CD133^hi^ cells ( > 80%; Figure 6a, 6b). Conversely, Notch^hi^ percentages were significantly lower in all samples after 24 hours in hypoxia (Figure 6c). To identify the mechanism of this observation, we interrogated the apoptotic and proliferative rates in the FACS-isolated subpopulations upon hypoxia (Figure 6d, 6e). TUNEL assay indicated that hypoxia dramatically increased apoptosis in Notch^hi^ but not CD133^hi^ cells (Figure 6di-6dii, Supplementary Figure 9a). We did not observe any significant change in the proliferation rate, as assayed by Ki67 immunostaining, after 24 hours of hypoxia (Figure 6ei-6eii, Supplementary Figure 9b).

We next tested the *in vitro* tumorsphere formation ability of FACS-isolated CD133^hi^ and Notch^hi^ cells under normoxia and hypoxia. CD133^hi^ cells showed equivalent sphere-formation efficiency in the two conditions. However, Notch^hi^ GSCs demonstrated a significant reduction in tumorsphere formation in hypoxia (GBML8: Figure 6fi, GBML20: Figure 6fii). These findings suggest that hypoxia permits tumor growth driven by CD133^hi^ GSCs, which are transcriptionally primed for anaerobic glycolysis. However, Notch^hi^ GSCs undergo apoptosis and, therefore, cannot contribute to tumor growth in hypoxic regions, consistent with their preferential localization to perivascular areas.

### Ectopic expression of NICD1 in CD133^hi^ cells suppresses anaerobic glycolysis and reduces tolerance to hypoxia

**Figure 7:**
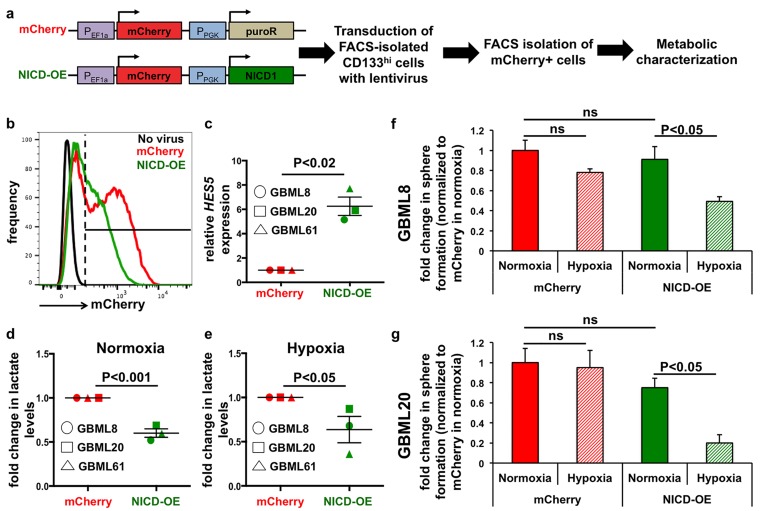
Ectopic Notch activation reprograms metabolism **a.** Schematic representation of the lentiviral constructs used for ectopic activation of the Notch pathway (NICD-OE) or the control vector (mCherry) and the experimental plan. **b.** Representative FACS plot from GBML8 cells that were infected with either NICD-OE or mCherry virus, showing transduction efficiency. **c.** FACS-isolated mCherry+ cells from GBML8, GBML20 and GBML61 cultures showed increased *HES5* transcript in the NICD-OE condition (*n* = 3 primary cultures, *t*-test, *P* < 0.02). **d**., **e**. Lactate level measurements in FACS-isolated mCherry+ cells after 24 hours of (**d).** normoxia or (e) hypoxia. NICD-OE led to significant downregulation in lactate levels in 3 primary cultures: (**d**) *t*-test, *P* < 0.001; (**e)** *t*-test, *P* < 0.05). (**f**-**g)** mCherry+ cells from GBML8 and GBML20 cells transduced with NICD-OE or mCherry virus were subjected to tumorsphere formation assay under normoxia or hypoxia. Ectopic Notch activation led to reduced tumorsphere formation under hypoxic conditions, whereas mCherry control did not show any change (*n* = 3/condition): **f.** GBML8: ANOVA, F_(3,8)_ = 6.543, *P* < 0.05; (**g**) GBML20: ANOVA, F_(3,12)_ = 8.256, *P* < 0.01. ns: not significant.

To test the hypothesis that Notch signaling regulates GBM cells’ response to hypoxia and their metabolism, we ectopically expressed NICD1 in FACS-isolated CD133^hi^ cells using a lentiviral vector, NICD-OE, which allowed identification of transduced cells by mCherry fluorescence (Figure 7a). After 7 days, mCherry+ cells were FACS-isolated and subjected to further characterization (Figure 7a, 7b). NICD-OE led to significant upregulation of the Notch transcriptional target *HES5* mRNA compared to mCherry control virus (6.25 ± 0.76 -fold), confirming ectopic activation of Notch signaling (GBML8, GBML20, GBML61; Figure 7c). When we compared lactate levels in cells transduced with NICD-OE *vs*. control cells, we observed significant reduction under both normoxia (0.61 ± 0.05 -fold of control, Figure 7d) and hypoxia (0.62 ± 0.14 -fold of control, Figure 7e).

To analyze the effects of ectopic Notch activation on tumorsphere formation, we plated freshly isolated mCherry+ cells in limiting dilutions (10 cells/μL), subjected the cells to 24 hours of hypoxia and analyzed sphere formation 7 days later. We found that NICD-OE led to a significant reduction in sphere formation in hypoxia compared to the mCherry control virus (GBML8 and GBML20: Figure 7f, 7g). These assays reproduced the phenotype seen with native CD133^hi^ and Notch^hi^ GSCs and suggested that ectopic activation of Notch signaling is sufficient to reprogram the metabolic profile of CD133^hi^ cells and suppress their tolerance to hypoxia.

## DISCUSSION

**Figure 8:**
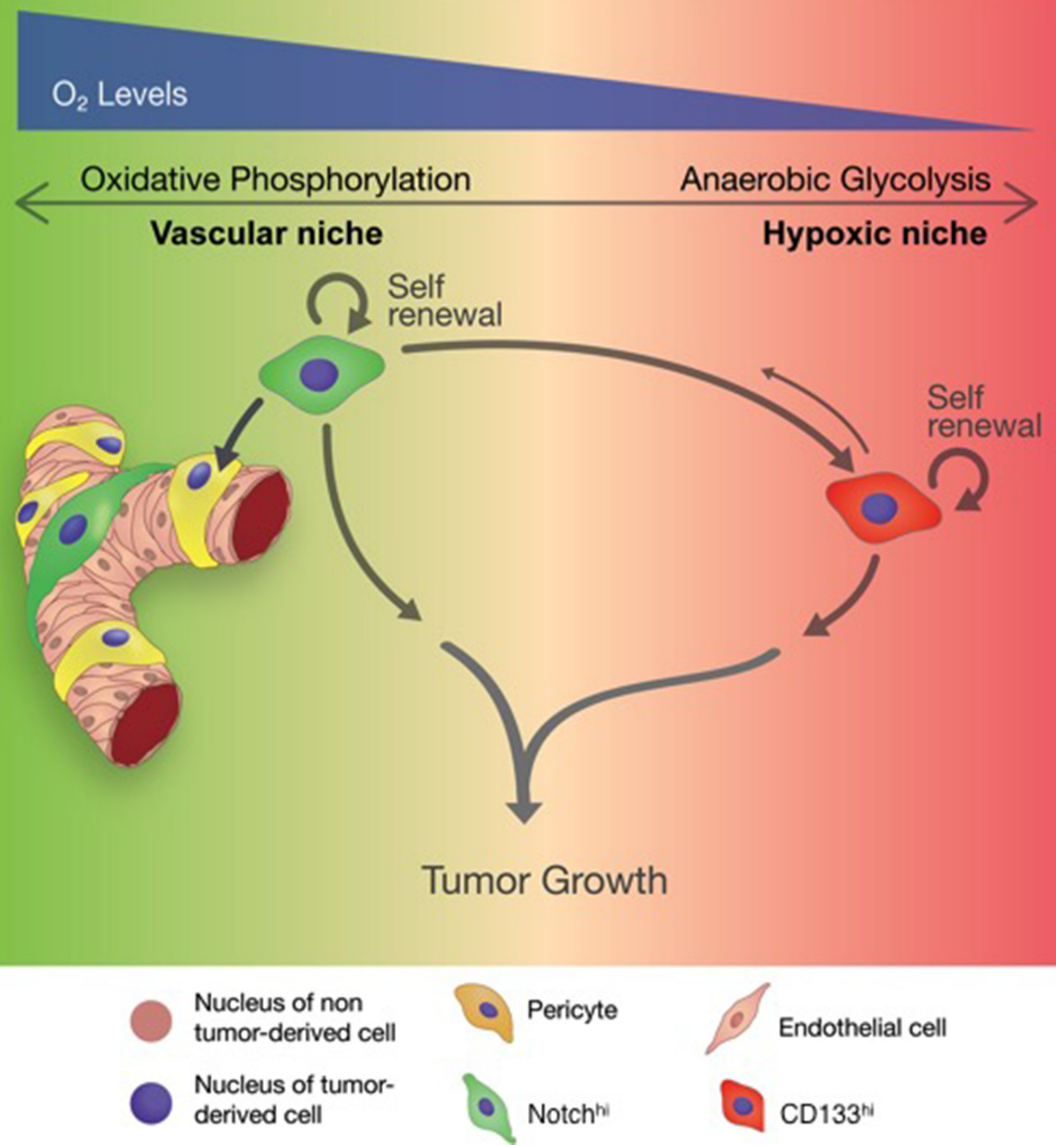
Schematic summarizing the role of distinct GSCs in tumor progression

Inter- and intra-tumoral heterogeneity in GBM represent major obstacles to therapy [12, 14-16]. Here, we provide a novel model for functional and metabolic intratumoral heterogeneity within GBM’s stem cell population. We discovered that activation of Notch signaling and CD133 (PROM1) cell surface expression, two well-characterized GSC markers, demonstrate only partial overlap (Notch^hi^/CD133^hi^ or DP cells). Notch^hi^/CD133^lo^ (Notch^hi^) and CD133^hi^/Notch^lo^ (CD133^hi^) cells, the non-overlapping cell populations, show profound functional differences reflected in their niches, metabolism and differentiation profiles (Figure 8). We propose that this heterogeneity allows GSCs to support tumor growth in a wide spectrum of tumor microenvironments. Our results are reproducible across GBM tumors with a wild-type IDH background, which represent the majority of adult GBM [12].

The tumor microenvironment in GBM is highly variable [11, 17, 64, 65]. Previous research demonstrated a perivascular niche for Nestin-expressing GSCs [18, 66]. However, immunohistochemical studies have demonstrated expression of CD133 not only in perivascular niches, but also in hypoxic regions [19, 20], supporting the idea that GSCs may also populate hypoxic microenvironments in GBM. Indeed, hypoxia and acidic stress promote the GSC phenotype [19, 21-26].

Our findings provide evidence for the co-existence of distinct GSC phenotypes in perivascular and hypoxic niches of any given tumor. In our model (Figure 8), which precisely mirrors our findings in GBM biospecimens, Notch^hi^ GSCs show a strong predilection for vascular niches, likely due to the presence of Notch ligands on endothelial cells. Notch^hi^ GSCs are not merely passive residents in perivascular microenvironments, but rather actively help create them, by transdifferentiating into pericytes. This finding is supported by recent literature showing that NICD1 overexpression induces pericytic differentiation in GBM [55]. We showed that the well-developed tumor vasculature in tumors initiated by Notch^hi^ cells produces adequate blood perfusion to prevent tumor hypoxia, and that the relatively aerobic metabolism of Notch^hi^ GSCs is precisely tuned to this microenvironment. Finally, we demonstrated that activation of Notch signaling is sufficient for metabolic reprogramming of tumor cells by suppressing anaerobic glycolysis.

Our model also shows that CD133^hi^ GSCs populate the tumor more diffusely, with reduced requirement for perivascular localization. This observation is consistent with previous demonstrations of CD133 immunoreactivity in both perivascular and hypoxic niches in GBM [19, 20]. The DP population in our study may indeed reflect this perivascular cell population that expresses CD133 and still relies on Notch signaling. However, our findings strongly suggest a hierarchical relationship between Notch^hi^ and CD133^hi^ cells. We postulate that, as the tumor expands and tumor cells move away from vessels, Notch^hi^ GSCs generate DP and, subsequently, CD133^hi^ lineages. We found that CD133^hi^ GSCs have limited differentiation capacity and do not generate Notch^hi^ cells or pericytes. Tumors initiated by CD133^hi^ cells are hypoxic due to underdeveloped vasculature and reduced blood perfusion. To adapt to this hypoxic microenvironment, CD133^hi^ GSCs specifically entrain a defined transcriptional network that allows them to bias glucose metabolism to anaerobic glycolysis rather than oxidative phosphorylation. While CD133^hi^ cells thrive in hypoxic conditions, Notch^hi^ GSCs undergo apoptosis, suggesting that their preference for aerobic glucose metabolism mandates a perivascular niche. Therefore, we propose that Notch^hi^ and CD133^hi^ GSCs support tumor growth *via* two parallel mechanisms.

We showed that canonical Notch signaling suppresses anaerobic glycolysis and confers vulnerability to hypoxia. This is a novel function of Notch signaling in GBM. Previous reports suggested that hypoxia is associated with induction of several components of the Notch pathway [24, 67]. We propose that this induction is compensatory, in order to boost vascularization and tissue oxygenation by Notch^hi^ lineages. Nonetheless, our data indicate a critical window of vulnerability during hypoxia: Notch^hi^ cells, which drive angiogenesis, are prone to apoptosis due to inability to rely on anaerobic glucose metabolism. Future experiments will need to determine the reason this inherent antithesis characterizes Notch signaling in GBM. One interesting possibility is that the broad differentiation potential of Notch^hi^ cells may mandate aerobic metabolism.

Previous reports suggested that blockade of Notch signaling depletes CD133^hi^ cells [6, 33, 50]. However, GSC sensitivity to blockers of Notch signaling is inversely related to the level of cell surface CD133 expression [51] and GBM cultures can expand in the presence of γ-secretase inhibitors [68], suggesting Notch-independent growth. Our model reconciles these discrepancies. While Notch^hi^ and CD133^hi^ cells represent largely distinct populations, we found that multipotent Notch^hi^ cells generate CD133^hi^ lineages. The reduction in CD133^hi^ cell counts after Notch inhibition can, therefore, be explained by depletion of the Notch^hi^ to CD133^hi^ differentiation program. The recent clinical trial with a Notch inhibitor demonstrated that tumors do recur despite Notch inhibition [50]. We explain this as tumor propagation by CD133^hi^ cells, which are resistant to Notch blockade, as we and Tanaka *et al.* [51] showed, and are already present at the time of Notch inhibition.

Our findings cannot rule out the possibility of GSC types other than Notch^hi^ and CD133^hi^ cells. For example, our xenograft studies demonstrate that the DN population does contain cells with tumor-initiating properties. However, the smaller size of tumors initiated by DN cells suggests a relatively low frequency of tumor-initiating cells in this population.

Overall, our observations suggest tumor progression in GBM is mediated by distinct GSCs populating vascular and hypoxic niches. This heterogeneity provides an escape mechanism for tumors treated with agents targeting a single subtype but not both. For example, most patients with GBM are resistant to anti-angiogenic therapies [2, 3, 69], likely due to selective kill of hypoxia-vulnerable cells but tumor progression by cells that tolerate hypoxia [70]. Likewise, Notch inhibitors failed to prevent recurrence [50], likely due to tumor lineages that do not depend on the Notch pathway. Our model provides a mechanistic understanding of both hypoxia-vulnerable (Notch^hi^) and -resistant (CD133^hi^) GSC lineages. We propose that future treatments will need to take into account this heterogeneity, in order to improve patient outcomes.

## MATERIALS AND METHODS

### GBM biospecimen immunostaining for NICD1

Chromogenic Immunohistochemistry was performed on formalin-fixed paraffin-embedded GBM tissue on a Discovery XT platform (Ventana Medical Systems Inc., Tucson, AZ) using Ventana’s reagents, unless otherwise noted. Six µm-thick sections were deparaffinized in xylene and rehydrated through graded alcohols. Heat induced antigen retrieval was performed in a BioCare Decloaking Chamber in Tris-EDTA buffer for 20 minutes at 120^°^C and 17 PSI and incubated in 3% hydrogen peroxide for 4 min. NICD1 antibody (1:100, Abcam, ab8925) was incubated for 3 hours and detected using anti-rabbit horseradish peroxidase multimer (OmniMap). Immune complexes were visualized with 3,3’-diaminobenzidene (DAB) and enhanced with copper sulfate (ChromoMap). Slides were counterstained with hematoxylin, dehydrated and mounted with permanent media.

### Primary tumor cultures

We followed a protocol approved by NYU Langone Medical Center’s Institutional Review Board (IRB) to procure fresh tumor tissue from consented patients undergoing surgery for resection of GBM (IRB# S12-01130). Primary human GBM cultures were obtained as previously described [44, 46, 47]. Four primary GBM cultures were used for the experiments described here (GBML8, GBML20 and GBML33 were used for NotchLenti experiments; GBML8, GBML20 and GBML61 were used for NICD-OE experiments) (Supplementary Figure 1). DNA was extracted from formalin-fixed paraffin embedded tissue and analyzed using Infinium 450k DNA methylation array, as described previously [47]. Tumors were classified according to their methylation profiles [48].

### Quantification of distance of cells from the vasculature

ImageJ was used for estimating the distance of tumor cells from the vasculature. Three μm-thick optical sections from confocal z-stacks were obtained. Vasculature was identified using CD105 immunostaining. To obtain the number of vessels and vascular area, images were thresholded (Otsu Thresholding, ImageJ) using tissue not treated with primary antibody as negative control, and converted to binary format. Vessels > 10 μm in length were counted. An average of 4 fields were calculated for each animal. In order to quantify the distance of cells from the vasculature, blood vessels were defined as above. Three regions of interest (ROIs) were defined for each image: promixal (0-15 μm from blood vessels), distal (15-30 μm) and outer ( > 15 μm). GFP and CD133 immunofluorescence was identified in each ROI. The estimate of number of cells/ROI was calculated by dividing the total GFP+ or CD133^hi^ positive area by the estimated average area of a cell (86.2 ± 9.7 µm^2^).

### Plasmids and constructs

NotchLenti reporter construct (pGreenFire) was obtained from Systems Biosciences. The NotchLenti-mCherry reporter was constructed by swapping the copGFP cassette with a mCherry cassette. The NotchLineage system was generated using two lentiviral constructs. The CreER^T2^ cassette and reporter construct were kind gifts of Dr. Philippe Ravassard (INSERM, France) [56]. CreER^T2^ was subcloned into the pGreenFire construct in place of copGFP. A loxP-DsRed-STOP-loxP-GFP lentiviral construct was used as the reporter for the NotchLineage experiments (Supplementary Figure 7ci). The lentiviral vector overexpressing NICD1 was generated by cloning the NICD coding frame into pLVX-mCherryN1 (Clontech), into the puromycin cassette. NICD1 plasmid which the subcloning was done was a kind gift of Dr. Iannis Aifantis (NYU School of Medicine).

### Lentivirus production

Lentiviruses were generated in Lenti-X 293 HEK (Clontech) producer cells after lipofection (Lipofectamine-2000, Life Technologies) with a combination of: transfer plasmid, encoding the viral genome; packaging plasmids (ViraPower Lentiviral expression systems, Life Technologies), encoding structural and enzymatic components of viral particles; and envelope plasmids, encoding viral envelope proteins. Lenti-X 293 HEK cells were cultured in Dulbecco’s Minimal Essential Media (DMEM, Life Technologies) supplemented with 10% FBS and non-essential amino acids. Lentiviral supernatant was collected at day 2 and 3 after transfection, filtered (0.45 μm filter) and concentrated with ultracentrifugation (28,000g for 3 hours at 4°C) using a 4% sucrose/PBS cushion. After centrifugation, the supernatant was discarded and viral pellets were resuspended in Opti-MEM medium, aliquoted and stored at -80°C. Titers were determined by flow cytometry or qPCR-based assays (ABM).

### Viral transduction

Primary GBM tumorsphere cultures were dissociated with Accutase (Innovative Cell Technologies). Cells (300 cells/μl) were incubated at 37°C overnight with lentivirus at a multiplicity of infection (MOI) of 5. Protamine sulfate (4 μg/mL) was added to facilitate viral transduction. Three days after transduction, transduced cells were selected with appropriate antibiotics. In the case of NotchLenti, selection with 1 μg/mL puromycin (Life Technologies) was performed for 5-7 days.

### Animals and stereotactic injections into mouse brain

Mice were housed within NYU Langone Medical Center’s Animal Facilities. All procedures were performed according to our IACUC-approved protocol (IACUC# 120310-03). 6-8 week old NOD.SCID male mice (Jackson Laboratory, NOD.CB17-Prkdcscid/J, 001303) were anesthetized with i.p. injection of ketamine/xylazine (10 mg/kg and 100 mg/kg, respectively). They were then mounted on a stereotactic frame (Harvard Apparatus). A midline skin incision was made. A high-speed drill was used to drill a small hole in the calvaria 2 mm off the midline and 2 mm anterior to coronal suture. Five μl of a suspension of human GBM cells (100,000 cells/μl, unless otherwise noted) were injected through a Hamilton syringe (1 μl/min, Harvard apparatus, needle pump) into the frontal lobe through the drilled hole. The injection needle was left in place for an additional 5 minutes after the injection was completed to prevent backflow. The skin incision was sutured and animals were monitored throughout the recovery period.

### Small animal MRI

Tumor formation was analyzed 1.5 months (unless otherwise noted) after injection of tumor cells into the brains of NOD.SCID mice. An MRI device bearing a 7-Tesla horizontal bore Bruker magnet (ID = 300mm with zero boil off technology) in the Small Animal Imaging Core Facility at NYU School of Medicine was used for imaging. Prior to imaging animals were anesthetized with isoflurane gas. Stacked images were processed using ImageJ software. Tumor volumes were calculated with Amira Software.

### Lineage tracing

GBM cells were transduced with the lentiviral NotchLineage system (Supplementary Figure 7ci). FACS-isolated DsRed+ cells were infected with a second lentivirus containing the driver construct. Transduced cells were selected with puromycin (1 μg/ml) for 7 days. Intracranial xenograft tumors were generated. Upon confirmation of tumors with MRI, two pulses of i.p. tamoxifen (150 mg/kg) were administered on sequential days. Animals were sacrificed 1 month after tamoxifen induction and immunofluorescence staining was performed.

### Flow cytometry

For flow cytometric analysis, cells were dissociated with Accutase. CD133 staining was performed with fluorophore-conjugated AC133 antibody (Miltenyi), which recognizes the CD133/1 epitope. The LSRII analyzer (BD Biosciences) was used for flow cytometric measurements. For fluorescence-assisted cell sorting (FACS), a FACSAria cell sorter (BD Biosciences) was used with assistance from the NYU Langone Medical Center’s Cytometry and Cell Sorting Core Facility staff.

### qRT-PCR expression analysis

Lysates and cDNA were prepared from 10,000 FACS-isolated cells using TaqMan Gene expression Cells-to-Ct kit using the manufacturer’s protocol (Ambion, Life Technologies). Taqman gene expression probes against *PROM1 (CD133), NOTCH1, NOTCH2, HES1, HES5, HEY1, DLL1, JAG1* AND *JAG2* (Supplementary Table 11a) (Ambion, Life Technologies) were used to analyze changes in gene expression with qRT-PCR (Applied Biosystems, StepOne Real-time PCR System). Fold changes in expression were calculated using the ΔΔCt method. The *HPRT1* gene was used to normalize results.

### Genomic DNA isolation and genomic PCR

Genomic DNA was isolated from 50,000 FACS-isolated cells using the DNeasy Blood and Tissue Kit (Qiagen). Genomic DNA (100 ng) was used in a nested-PCR to amplify copGFP DNA with the GoTaq DNA Polymerase kit (Promega) (Supplementary Table 11b). pGreenFire plasmid DNA was used as a positive control. PCR products were analyzed with 1.2% agarose gel electrophoresis.

### Immunofluorescence staining and microscopic analysis

When the experimental end-point was reached, animals were anesthetized with Ketamine/Xylazine (10 mg/kg and 100 mg/kg, respectively) and systemically perfused with first Phosphate-buffered Saline (PBS) and then 4% paraformaldehyde. Isolated brain tissue was mounted in OCT (Tissue-Tek) and 30 μm-thick frozen sections were obtained using a cryostat (Leica). Sections were blocked with 10% (w/v) BSA (Sigma), 0.1% Triton X-100 (Sigma) in PBS for 2 hours at room temperature. The primary antibodies and the dilutions used for immunostaining are summarized in Supplementary Table 12. Staining was performed in blocking solution for 18 hours at 4°C. Alexa488, Alexa555 and Alexa647 - conjugated secondary antibodies were used for fluorescent labeling (Life Technologies). Nuclear chromatin was counterstained with DAPI (Sigma). Epifluorescence microscopy was performed on an Eclipse E800 fluorescent microscope (Nikon). For confocal imaging, 30 μm z-stacks were obtained with an LSM700 confocal microscope (Zeiss). Image analyses were performed on ImageJ and Adobe Photoshop.

For apoptosis analysis, Click-iT Alexa Fluor 647 imaging assay (Life Technologies) was used according to the manufacturer’s protocol.

### RNA isolation, library preparation, RNA-sequencing and bioinformatics

RNA was isolated from 30,000 FACS-isolated GBM cells using mirRNeasy Micro RNA isolation kit (Qiagen). RNA-Seq libraries were prepared using the Epicentre (Illumina) TotalScript RNA-Seq kit, starting from 5 ng of DNAse I-treated total RNA, and using oligo-(dT) as the primer for cDNA synthesis. The libraries were pooled equimolarly and run on a HiSeq 2500 sequencing system, as paired 50 nucleotide reads. We sequenced 4 cell populations (CD133^hi^, Notch^hi^, DP and DN) from 2 patient samples (GBML8 in duplicates; and GBML20). A total of two flow cells were used. GBML8 samples (4 cell populations in duplicates) were run in one flow cell; and GBML20 samples (4 cell populations) samples were run in another. Sequencing results were de-multiplexed and converted to FASTQ format using Illumina Bcl2FastQ software. Paired-end reads were aligned to the human genome (build hg19/GRCh37) using the splice-aware STAR aligner [71]. PCR duplicates were removed using the Picard toolkit (http://broadinstitute.github.io/picard). HTSeq package was utilized to generate counts for each gene based on how many aligned reads overlap its exons [72]. On average we obtained 116,558,873 ± 10,631,963 reads. The lowest number of reads obtained was 83,887,138 and the highest was 158,844,383. These counts were then used to test for differential expression using negative binomial generalized linear models implemented by the DESeq2 R package [72]. For cumulative data analysis, GBML8 biological duplicates were first averaged. Then, that average was averaged with GBML20 data.

Gene set enrichment analysis (GSEA [57], http://software.broadinstitute.org/gsea/, Broad Institute) was performed on complete transcriptomes from CD133^hi^ and Notch^hi^ cells with genes pre-ranked by fold change, as well as on differentially expressed genes. Gene Ontology analysis with DAVID [62] (https://david.ncifcrf.gov, NIH) was performed on differentially expressed genes. RNA-seq and GSEA data and analyses are accessible through GEO accession number GSE99180.

### Western blotting

GBM cells were lysed in Lysis Buffer (150 mM NaCl, 50 mM Tris pH 7.4, 1 mM EDTA, 0.1% Triton-X100, 10% glycerol) supplemented with complete protease inhibitor cocktail (Roche). Lysates were centrifuged to remove debris and the supernatant was quantified using the Bradford assay. The supernatant was separated on an SDS-PAGE gel and transferred to a nitrocellulose membrane (Biorad). The membrane was probed with the following primary antibodies: anti-Hif1α (Bethyl Laboratories); and anti-β-Actin (Santa Cruz Biotechnology). Signal was detected with appropriate HRP-conjugated secondary antibodies (Life Technologies) suited for chemiluminescence (Thermo Scientific).

### 24-hour hypoxia treatment

Cells were treated with hypoxic gas mixture (1% O_2_, 5%CO_2_, balanced with N_2_) for 24 hours at 37°C using a hypoxia chamber (Stem Cell Technologies).

### Lactate and intracellular pH measurements

Extracellular and intracellular lactate from cell culture medium and cell lysates, respectively, was measured with a colorimetric assay (Eton Bioscience). For pH recordings, FACS-isolated GBM cells were plated onto laminin-coated coverslips and left to settle for 1-2 hours before recording. Coverslips with attached cells served as the floor of a submersion chamber mounted on the stage of an Olympus IX-73 inverted microscope equipped for epifluorescence. The cells were loaded with pH-sensitive fluorophore BCECF (2′,7′-bis-(2-carboxyethyl)-5-(and-6)-carboxyfluorescein) by incubation with 2 μM of the acetoxy-methylester (Life Technologies, CA) for 10 min at room temperature. During the experiment the cells were perfused at the rate of 2 ml/min with warmed (32°C) standard bicarbonate-buffered saline containing (in mM): 124 NaCl, 26 NaHCO_3_, 3.0 KCl, 1.0 NaH_2_PO_4_, 2.0 CaCl_2_, 1.5 MgCl_2_, and 10 glucose. Solution was gassed with 95% O2 and 5% CO2 and had a nominal pH of 7.4. A 75 W xenon lamp and a monochromator provided alternate 490 and 440 nm fluorescence excitation. For each excitation wavelength, the respective emissions above 535 nm (F490 and F440, respectively) were collected *via* a 40x oil-immersion objective and an intensified CCD camera. Averaged fluorescence from regions of interest around single cells was imaged using ImageMaster software (Photon Technology International). The F490:F440 ratios were converted to pH_i_ (intracellular pH) using the nigericin single point technique, applying a HEPES-buffered calibration solution (150 mM K^+^ and 3 μM nigericin, pH 7.0) at the end of each experiment [20]. Data were referenced to a calibration curve previously constructed using nigericin-150 mM K^+^ solutions buffered with PIPES or HEPES over the pH range of 6.0-8.0.

### Statistical analysis

Statistical comparisons included Student’s unpaired two-tailed *t*-test; one-way and two-way analysis of variance (ANOVA), followed by *post hoc* analysis with Tukey’s test; and Wilcoxon signed-rank test. Statistical significance cutoff was set at *p* < 0.05. Prism (GraphPad) and SPSS software (IBM) were used for statistical analyses. Population statistics were represented as mean ± standard error (SE) of the mean. Number of experiments and the specimens used were clarified in the figure legends for each experiment.

## SUPPLEMENTARY MATERIALS FIGURES AND TABLES




