# Supplementary material for: Notch signaling regulates metabolic heterogeneity in glioblastoma stem cells
Source: Oncotarget. 2017 May 23;8(39):64932–53. doi: 10.18632/oncotarget.18117 (PMC5630302; doi:10.18632/oncotarget.18117)
Supplement: Supplementary file 1 [file oncotarget-08-64932-s001.pdf]

Notch signaling regulates metabolic heterogeneity in glioblastoma stem cells

Supplementary Material

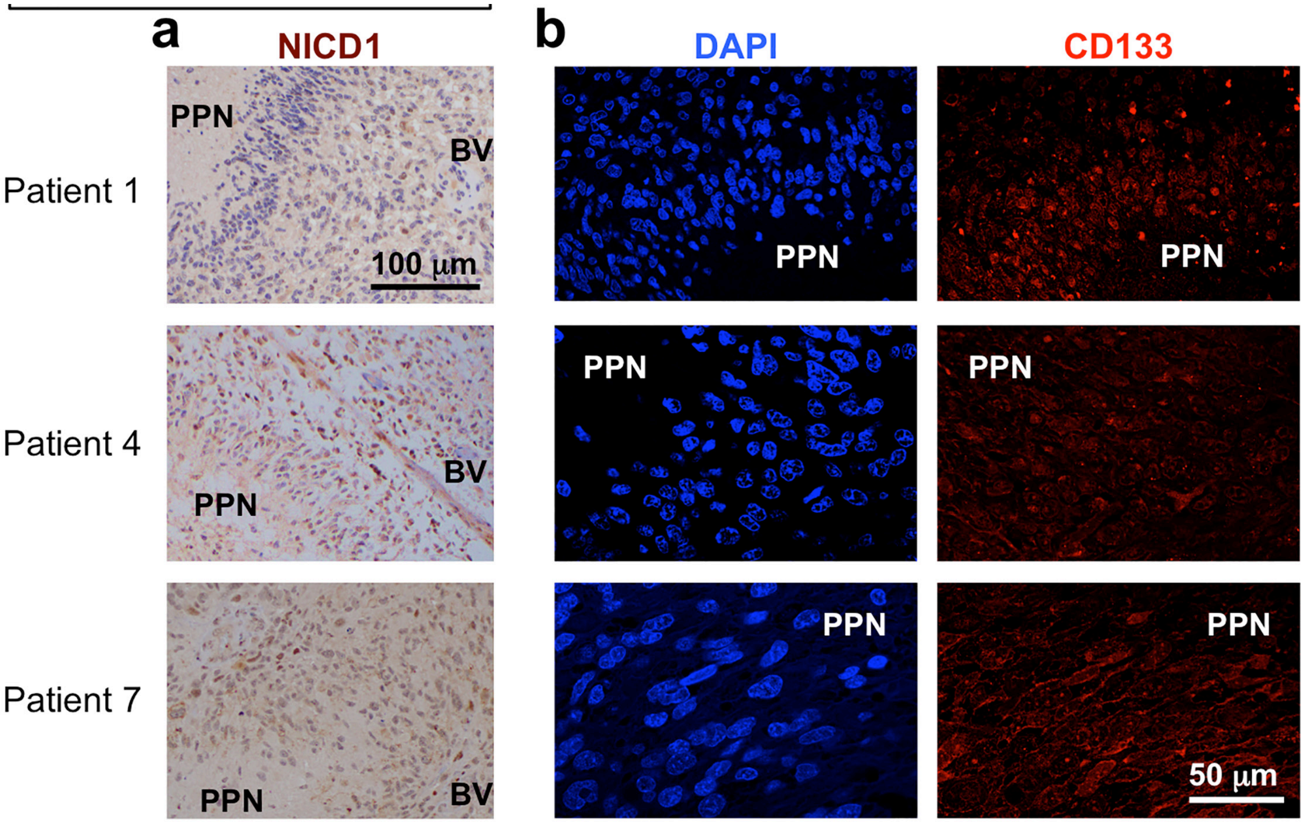

Supplementary Figure 1: NICD1 and CD133 immunoreactivity in human GBM biospecimens. (a) Representative examples of NICD1 immunohistochemistry in 3 patient biospecimens. Note the lack of NICD1 staining in the PPN and the nuclear immunoreactivity in perivascular areas. (b) CD133 immunofluorescent staining in PPN areas from the same 3 biospecimens shown in (a). Unlike NICD1, CD133 is expressed in PPN cells. PPN: pseudopalisading necrosis; BV: blood vessel.

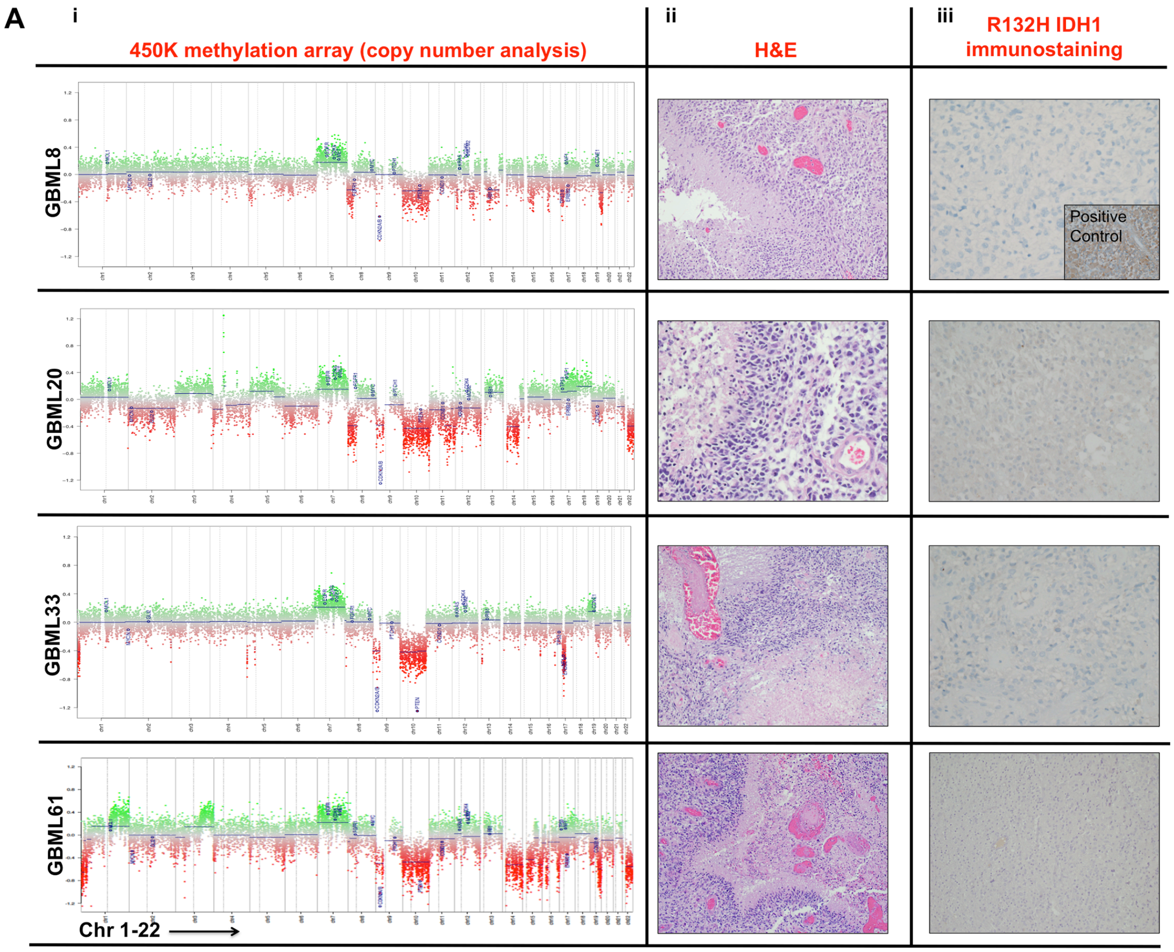

B

Patient sample	Score/subtype by DNA Methylation array	Score/subtype by RNA-seq	Cytogenetics	Other implied changes from CNV analysis	IDH1 mutations	MGMT methylation
**GBML8**	Mesenchymal	Mesenchymal	7+, 9p-, 10-, 17q-, 19p+	CDKN2A loss, PTEN loss, NF1 loss	IDH wild-type	Methylated
**GBML20**	GBM RTK1 (proneural)	Proneural	PDGFRA amp, 7+, 8p-, 10-, 11q-, 14-, 17+	PTEN loss	IDH wild-type	Not methylated
**GBML33**	GBM RTK1 (proneural)	N/A	5p-, 7+, 8p-, 10-, 12q-, 13q-, 17p-, 19q-	PTEN loss, RB1 loss, TP53 loss	IDH wild-type	Not methylated
**GBML61**	GBM RTK1 (proneural)	N/A	1p- 3q+, 7+, 9p-, 10-, 14-, 15q-, 19q-, 22-	PTEN loss	IDH wild-type	Not methylated

Supplementary Figure 2: Molecular characterization of primary GBM lines GBML8, GBML20, GBML33 and GBML61. (a,i) Copy number variation analysis performed by 450K DNA methylation data. (ii) H&E staining of tumor biospecimens showing pseudopalisading necrosis and microvascular proliferation. (iii) Negative immunostaining for the R132H IDH1 mutation. The inset shows a positive control from a different tumor. (b) Table summarizing the molecular subtype and genomic characteristics of the patient samples used in this study.

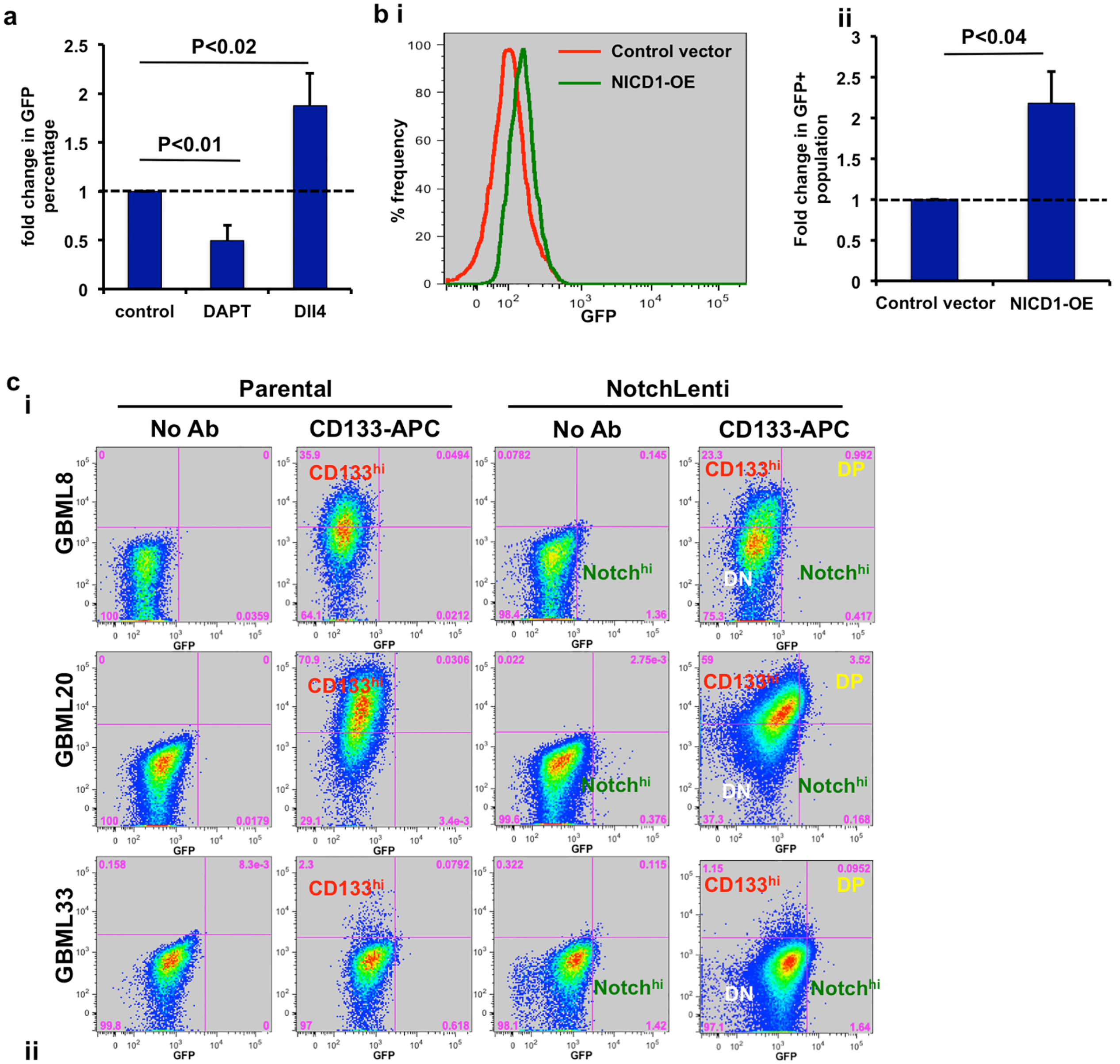

ii

	CD133^{hi}	Notch^{hi}	DP	DN
GBML8	30.73±15.96	1.13±0.49	0.56±0.23	67.58±16.00
GBML20	69.37±6.96	0.16±0.01	1.76±0.89	28.70±6.23
GBML33	3.79±2.52	1.68±0.88	0.10±0.01	94.43±1.84
**Cumulative (n=3 samples)**	34.63±19.02	0.99±0.45	0.81±0.49	63.57±19.08

Supplementary Figure 3: Validation of NotchLenti reporter. (a) Flow cytometric quantitation of the abundance of GFP+ cells in GBML8 cultures treated with 10 μM DAPT (n=3, t-test, P<0.01) or 100 ng/ml Dll4 (n=3, t-test, P<0.02). (b, i-ii) The abundance of GFP+ cells increased after overexpression of NICD (GBML8, n=3, t-test, P<0.04). (c, i) Flow cytometric analysis of 3 primary GBM lines modified with NotchLenti reveals the segregation between CD133^{hi} and Notch^{hi} populations. (c, ii) Table summarizing the cumulative statistics of GSC subpopulations in three different primary GBM lines.

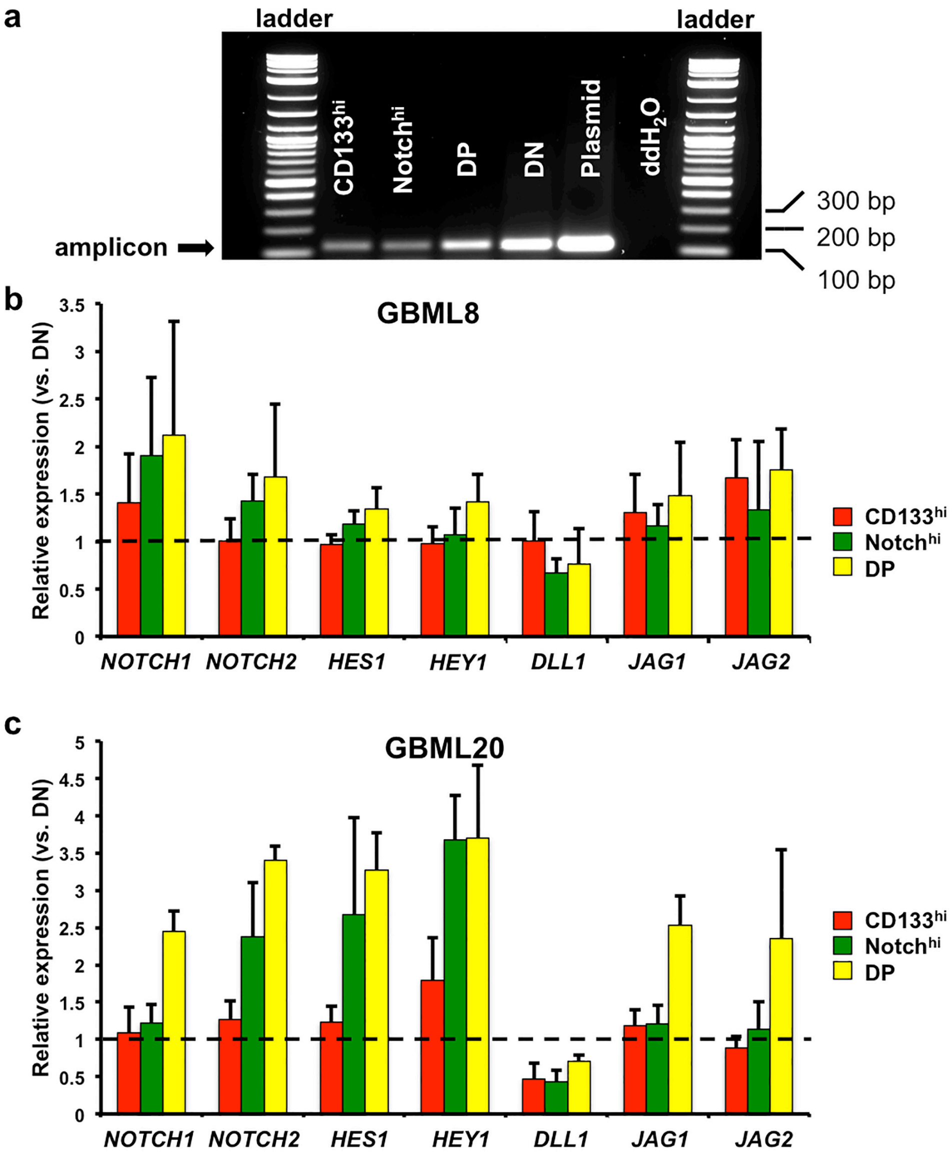

Supplemental Figure 4: Quality controls for FACS-isolated cell populations. (a) Genomic PCR for copGFP was performed on genomic DNA isolated from FACS-sorted populations. The transgene was present in all cell types. The lentiviral plasmid encoding NotchLenti was used as positive control. (b,c) CD133^{hi}, Notch^{hi}, DP and DN populations were FACS-isolated from GBML8 (b) and GBML20 (c) and pertinent transcripts were quantified with qRT-PCR.

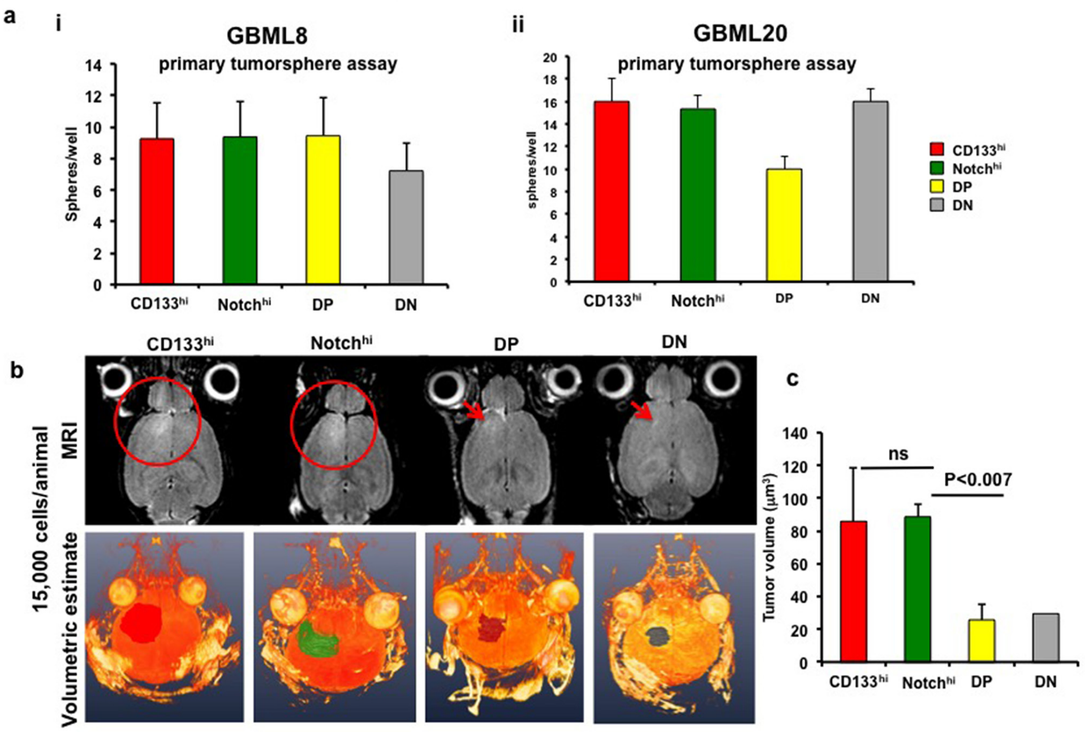

d **primary tumor initiation**

	GBML8				GBML20			
Injection (cells/animal)	CD133hi	Notchhi	DP	DN	CD133 hi	Notchhi	DP	DN
1.5 x 10⁴	3/3	3/3	3/3	2/2	-	-	-	-
1.5 x 10³	5/5	5/5	-	-	3/3	3/3	-	-

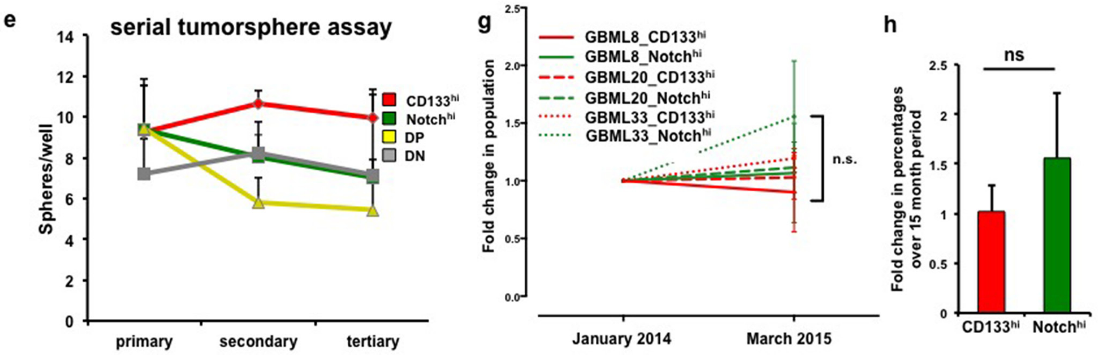

f **secondary tumor initiation**

Cells/animal	CD133hi	Notchhi	DN
1.5x10⁴	3/3	3/3	1/2

Supplementary Figure 5: CD133hi and Notchhi cells fulfill cancer stem cell criteria: self-renewal and tumorigenicity. (a) After FACS isolation, cells were seeded at low density (10 cells/μl) and tumorsphere formation was analyzed 7 days after isolation. The number of spheres formed did not change among distinct subpopulations: (i) n=5 for GBML8; (ii) n=3 for GBML20. (b) All four populations were able to form orthotopic tumor xenografts in mice. MRI was obtained 4 months after cell implantation. Volumetric estimates were obtained with Amira software (representative images of GBML8 was shown). (c) While the size of tumors derived from CD133hi and Notchhi cells was equivalent, Notchhi generated larger tumors than DP and DN cells (GBML8, n=3/condition, except n=2 in DN condition, t-test, P<0.007). (d) Table summarizing the number of animals injected with each cell type and tumor formation efficiency. (e) All populations were able to maintain sphere formation upon serial passaging (GBML8, n=5). (f) Table summarizing efficiency of secondary tumor formation from GSCs isolated from a xenograft tumor generated using GBML20 cells. (g,h) The abundance of CD133hi and Notchhi GSCs in 3 primary cultures is stable during a 15-month period. ns: not significant.

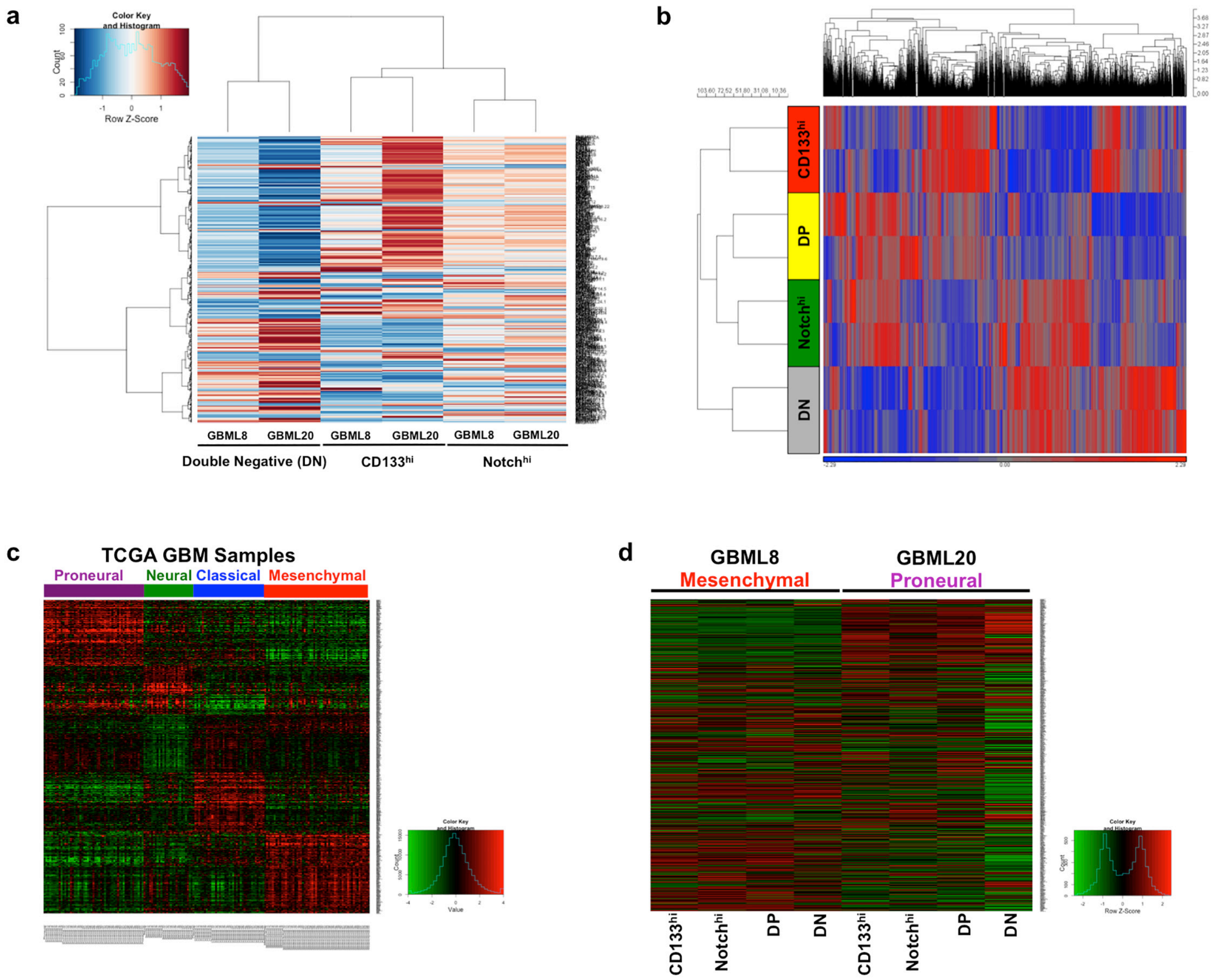

Supplementary Figure 6: CD133^{hi} and Notch^{hi} cells have distinct transcriptional profiles. (a) Supervised hierarchical clustering analysis using 420 differentially expressed genes among CD133^{hi}, Notch^{hi} and DN cells from two different GBM cultures. (b) Unsupervised hierarchical clustering analysis using all expressed genes among CD133^{hi}, Notch^{hi}, DP and DN cells from two different GBM cultures. (c) Supervised hierarchical clustering of 448 genes in 174 TCGA core samples reveals molecularly distinct GBM subtypes. (d) Supervised hierarchical clustering of GBML8 and GBML20 subpopulations using the same 448 genes shows that the molecular signature of the parent tumor is preserved in all four subpopulations (CD133^{hi}, Notch^{hi}, DP, DN).

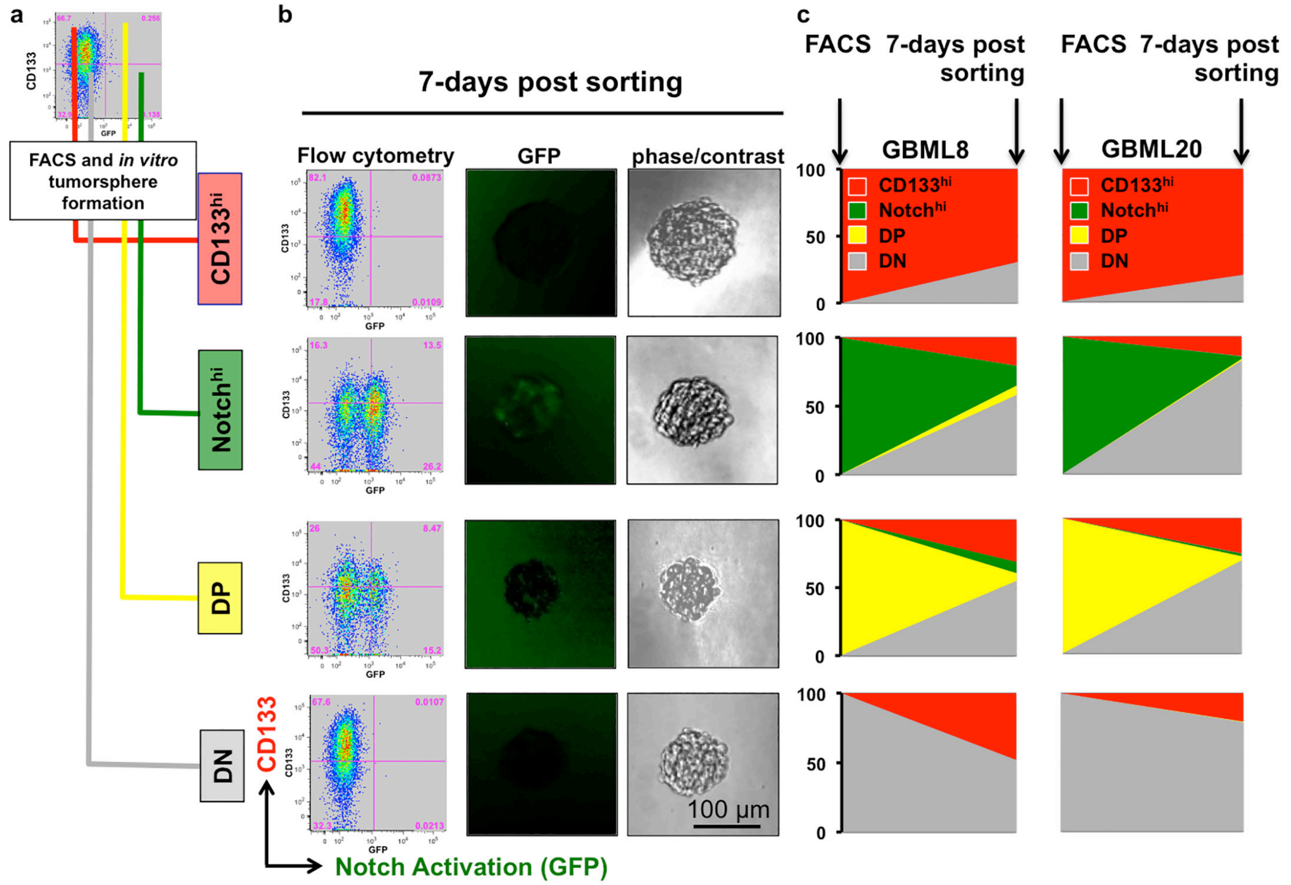

Supplementary Figure 7: Notch^{hi} cells reside higher in the cellular hierarchy in vitro. Cells were isolated with FACS (a) and their progeny were analyzed 7 days later with epifluorescence microscopy (representative images of GBML8 is shown) (b) and flow cytometry (c) (n=5 for GBML8 and n=3 for GBML20).

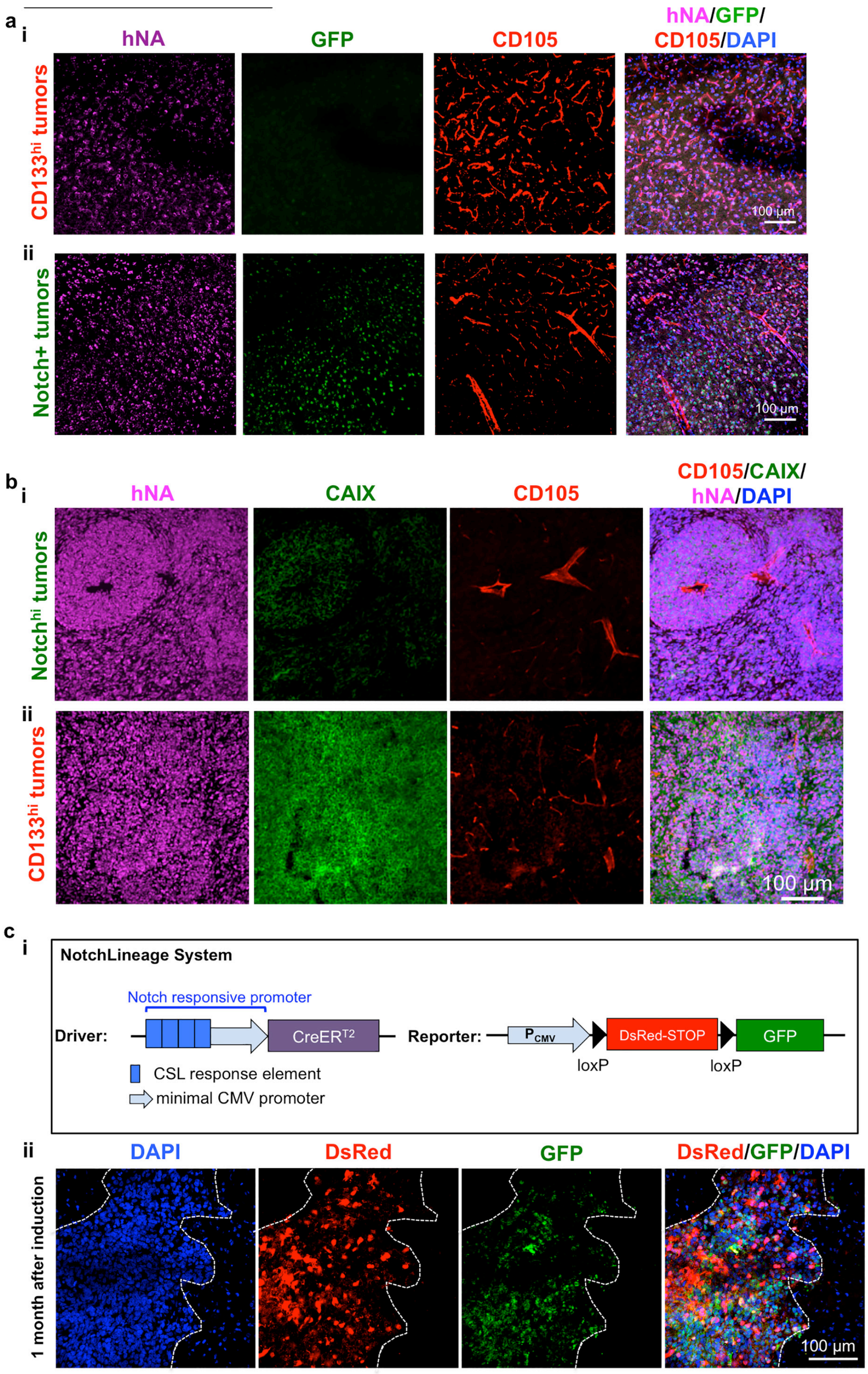

Supplementary Figure 8: Vascular properties of tumor xenografts derived from Notch^{hi} and CD133^{hi} cells. (a) FACS-isolated CD133^{hi} (top panel) and Notch^{hi} (bottom panel) cells from culture GBML20 were injected into the mouse brain (1500 cells/animal, n=3 animals/condition) and tumors were analyzed 8 months after injection. (b) Tumors generated by either CD133^{hi} or Notch^{hi} cells from culture GBML8 were analyzed for expression of hypoxia marker CAIX. Enrichment of CAIX was observed in CD133^{hi} tumors (n=3 tumors/condition). (c,i) Driver and reporter constructs used to generate the NotchLineage GBM cell line (GBML8). (c,ii) Immunofluorescent analysis of DsRed+ xenograft tumors, 1 month after induction with tamoxifen. GFP+ groups of cells mark the progeny of Notch^{hi} GSCs.

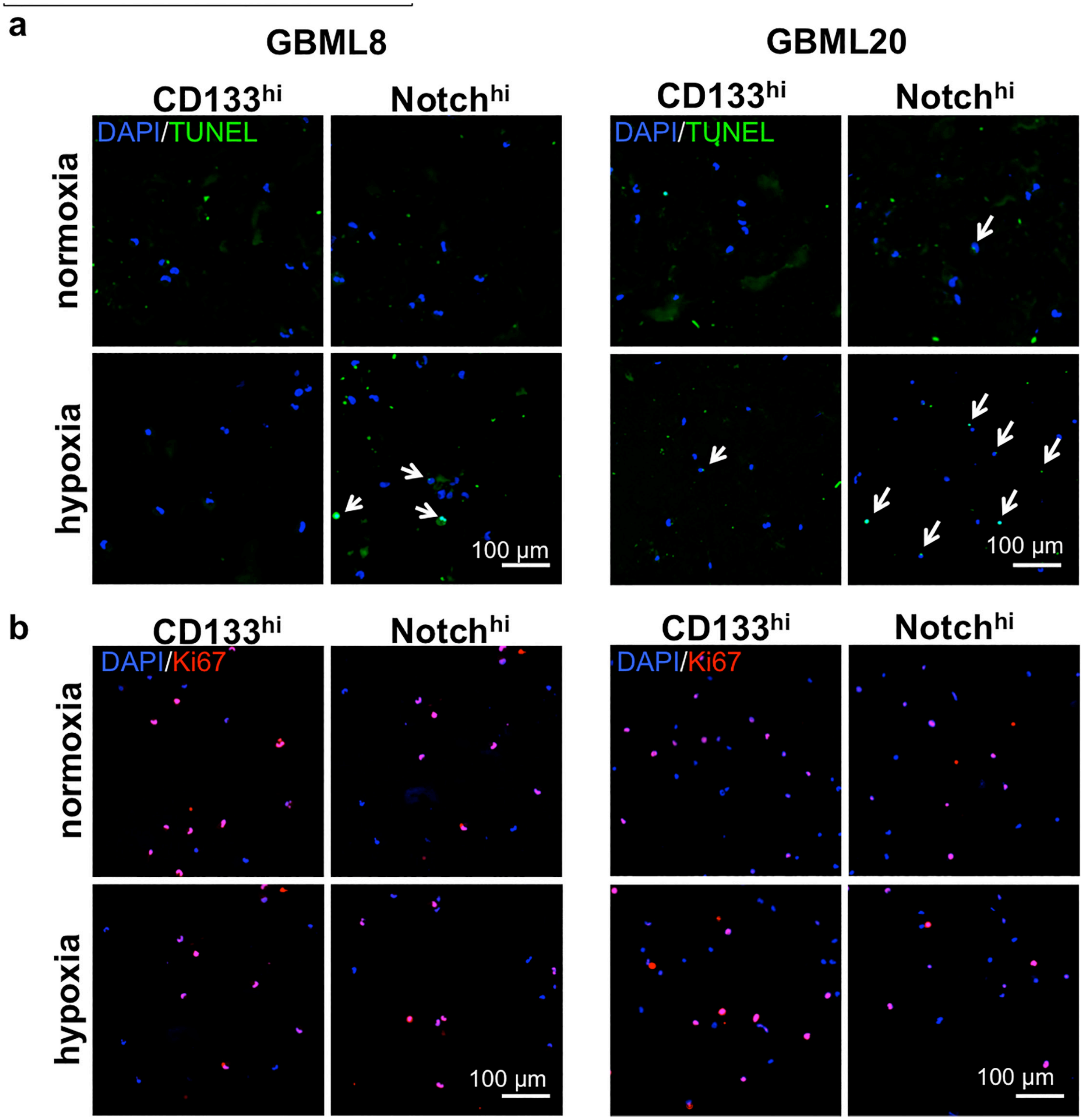

Supplementary Figure 9: Apoptosis and proliferation analysis in CD133^{hi} and Notch^{hi} cells after 24 hours of hypoxia. TUNEL assay showing apoptotic cells (a) and Ki67 immunofluoresence microscopy showing dividing cells (b) in FACS-isolated CD133^{hi} and Notch^{hi} populations from GBML8 (left panel) and GBML20 (right panel), under normoxic and hypoxic conditions. Arrows: TUNEL+ cells.
