# Supplementary material for: Notch signaling regulates metabolic heterogeneity in glioblastoma stem cells
Source: Oncotarget. 2017 May 23;8(39):64932–53. doi: 10.18632/oncotarget.18117 (PMC5630302; doi:10.18632/oncotarget.18117)
Supplement: Supplementary file 2 [file oncotarget-08-64932-s002.docx]

**SUPPLEMENTARY FIGURE LEGENDS, TABLES**

**SUPPLEMENTARY FIGURE LEGENDS**

**SUPPLEMENTARY TABLES**

**Supplementary Table 1. List of patient samples used for NICD1 and CD133 expression analysis.**

**Supplementary Table 2. Genes upregulated in CD133^hi^ vs. DN cells.**

**Supplementary Table 3. Genes upregulated in Notch^hi^ vs. DN cells.**

**Supplementary Table 4. Genes upregulated in both CD133^hi^ and Notch^hi^ vs. DN cells.**

**Supplementary Table 5. Genes downregulated in CD133^hi^ vs. DN cells.**

**Supplementary Table 6. Genes downregulated in Notch^hi^ vs. DN cells.**

**Supplementary Table 7. Genes downregulated in both CD133^hi^ and Notch^hi^ vs. DN cells.**

**Supplementary Table 8. Gene Set Enrichment Analysis on CD133^hi^ and Notch^hi^ transcriptomes.**

**Supplementary Table 9. Genes differentially expressed in CD133^hi^ *vs.* Notch^hi^ cells.**

**Supplementary Table 10. Top 10 (a) GSEA gene sets and (b) DAVID GO terms enriched in CD133^hi^ *vs.* Notch^hi^ cells.**

**Supplementary Table 11. Taqman Gene expression assays (a) and primers (b) used in the study.**

**Supplementary Table 12. Antibodies used in the study.**

**Supplementary Table 1. Patients whose tumors were used for NICD1 and CD133 expression by immunomicroscopy.**

| **Patient** | **Age** | **Sex** | **Diagnosis** | **IDH1 R132H status** | **PPN present** | **NICD1 in PPN** | **Perivascular NICD1** | **CD133 in PPN** | **Perivascular CD133** |
| --- | --- | --- | --- | --- | --- | --- | --- | --- | --- |
| 1 | 65 | F | GBM (WHO IV) | wt | yes | - | + | + | + |
| 2 | 63 | M | GBM (WHO IV) | wt | no | n/a | + | n/a | + |
| 3 | 60 | M | GBM (WHO IV) | wt | no | n/a | + | n/a | + |
| 4 | 65 | M | GBM (WHO IV) | wt | yes | - | + | + | + |
| 5 | 85 | M | GBM (WHO IV) | wt | no | n/a | + | n/a | + |
| 6 | 79 | F | GBM (WHO IV) | wt | yes | - | + | + | + |
| 7 | 83 | F | GBM (WHO IV) | wt | yes | - | + | + | + |
| 8 | 48 | F | GBM (WHO IV) | wt | no | n/a | + | n/a | + |
| 9 | 69 | M | GBM (WHO IV) | wt | no | n/a | + | n/a | + |

n/a: not applicable; +, present; -, absent

**Supplementary Table 2. Genes upregulated in CD133^hi^ vs. DN cells (239 genes, P<0.05)**

| **gene ID** | **log2 change** | **fold change** | **P value** |
| --- | --- | --- | --- |
| PROM1 | 2.93489 | 7.646979465 | 0 |
| FAM107A | 2.50757 | 5.686614476 | 0.002 |
| NDUFA4L2 | 2.10326 | 4.296792177 | 0.002 |
| OLFML2A | 1.95692 | 3.882322591 | 0 |
| IL37 | 1.94602 | 3.853100991 | 0.01 |
| CTB-1144G6.5 | 1.93655 | 3.827891665 | 0.027 |
| ISLR | 1.92006 | 3.784387972 | 0.028 |
| PLA2G7 | 1.91063 | 3.759732449 | 0.018 |
| VCAM1 | 1.90117 | 3.735159886 | 0.019 |
| FGFBP2 | 1.8719 | 3.660142962 | 0.02 |
| AP1M2 | 1.85711 | 3.622812147 | 0.034 |
| CCDC89 | 1.84612 | 3.595319544 | 0.001 |
| ACAN | 1.83823 | 3.575710658 | 0.034 |
| BMP6 | 1.78327 | 3.442054642 | 0.007 |
| PCSK1 | 1.77133 | 3.413685145 | 0 |
| RN7SL124P | 1.768 | 3.405814831 | 0.037 |
| ALPK2 | 1.74046 | 3.341416911 | 0.03 |
| CDK1 | 1.7355 | 3.329948811 | 0.003 |
| MAMDC2 | 1.73488 | 3.328518069 | 0.015 |
| AC005703.3 | 1.70988 | 3.271336121 | 0.045 |
| AC007750.5 | 1.70523 | 3.260809139 | 0.048 |
| CDCA3 | 1.7025 | 3.254644571 | 0.003 |
| COL9A1 | 1.66998 | 3.182101821 | 0.021 |
| MXD3 | 1.66387 | 3.168653706 | 0.012 |
| AURKB | 1.65706 | 3.153731854 | 0.019 |
| C10orf10 | 1.64202 | 3.121025187 | 0.031 |
| KIF23 | 1.63886 | 3.114196546 | 0.004 |
| FAM83D | 1.63365 | 3.10297054 | 0.016 |
| NDC80 | 1.61826 | 3.07004542 | 0.006 |
| ESPL1 | 1.61569 | 3.064581346 | 0.002 |
| PIF1 | 1.58945 | 3.00934602 | 0.008 |
| GALNT15 | 1.58518 | 3.000452311 | 0.014 |
| CCNA2 | 1.57992 | 2.989532718 | 0.003 |
| RRM2P3 | 1.5775 | 2.984522231 | 0.024 |
| TOP2A | 1.56203 | 2.95269021 | 0.035 |
| CDC25C | 1.55739 | 2.943209014 | 0.007 |
| NEIL3 | 1.54942 | 2.926994429 | 0.006 |
| CDH5 | 1.5475 | 2.923101651 | 0.026 |
| NEK2 | 1.5119 | 2.851853752 | 0.008 |
| COLCA1 | 1.49623 | 2.821045627 | 0.046 |
| RP11-726G1.1 | 1.49459 | 2.817840593 | 0.002 |
| TTK | 1.48886 | 2.806671078 | 0.005 |
| KIF2C | 1.45942 | 2.749977853 | 0.008 |
| ESCO2 | 1.44718 | 2.7267454 | 0.014 |
| C2orf48 | 1.44619 | 2.724874906 | 0.018 |
| FAM72C | 1.44102 | 2.715127599 | 0.012 |
| RRM2 | 1.43805 | 2.709543859 | 0.009 |
| E2F8 | 1.43487 | 2.703578038 | 0.007 |
| KIF11 | 1.4316 | 2.697457071 | 0.01 |
| SPC25 | 1.43135 | 2.696989678 | 0.012 |
| CASP4 | 1.41738 | 2.671000044 | 0.033 |
| HJURP | 1.41613 | 2.668686802 | 0.013 |
| LINC00341 | 1.40936 | 2.656193042 | 0.019 |
| KIFC1 | 1.40286 | 2.644252598 | 0.007 |
| RP13-735L24.1 | 1.4026 | 2.643776098 | 0.038 |
| KBTBD11-OT1 | 1.39188 | 2.624204225 | 0.041 |
| PLK1 | 1.38233 | 2.606890527 | 0.016 |
| LAMA3 | 1.37861 | 2.600177299 | 0.011 |
| EMP2 | 1.37474 | 2.593211723 | 0.011 |
| RP11-439C15.4 | 1.36944 | 2.58370257 | 0.022 |
| CKAP2L | 1.36901 | 2.582932604 | 0.011 |
| RTKN2 | 1.36284 | 2.571909717 | 0.012 |
| CENPI | 1.36054 | 2.567812746 | 0.023 |
| ARHGAP11A | 1.3478 | 2.545237001 | 0.009 |
| NCAPG | 1.34232 | 2.535587393 | 0.007 |
| NUSAP1 | 1.33721 | 2.526622274 | 0.01 |
| LINC00669 | 1.33618 | 2.524819057 | 0.024 |
| SHCBP1 | 1.33098 | 2.515735068 | 0.012 |
| LOXL2 | 1.32871 | 2.511779812 | 0.038 |
| AC004381.6 | 1.32719 | 2.509134835 | 0.006 |
| ADCY8 | 1.31587 | 2.489524132 | 0.024 |
| ARHGAP11B | 1.30345 | 2.468184086 | 0.01 |
| CASC1 | 1.30035 | 2.462886254 | 0.05 |
| APLN | 1.29704 | 2.457242089 | 0.002 |
| SKA1 | 1.2887 | 2.443078127 | 0.009 |
| KIF15 | 1.28495 | 2.436736075 | 0.017 |
| DEPDC1 | 1.28459 | 2.436128105 | 0.02 |
| STIL | 1.27563 | 2.421045176 | 0.031 |
| NCAPH | 1.27529 | 2.420474676 | 0.016 |
| ANGPTL4 | 1.27305 | 2.416719442 | 0.03 |
| CDCA8 | 1.27231 | 2.415480155 | 0.011 |
| KIF18B | 1.2691 | 2.41011168 | 0.023 |
| RP11-536O18.2 | 1.2683 | 2.408775601 | 0.027 |
| INCENP | 1.26147 | 2.397398938 | 0.029 |
| CENPA | 1.26118 | 2.396917079 | 0.034 |
| ASPM | 1.26049 | 2.395770976 | 0.031 |
| UBE2C | 1.25681 | 2.389667675 | 0.021 |
| CDCA5 | 1.25237 | 2.382324602 | 0.003 |
| TACC3 | 1.24222 | 2.365622712 | 0.015 |
| MTFR2 | 1.23771 | 2.358239101 | 0.026 |
| CEP55 | 1.23722 | 2.35743828 | 0.03 |
| KPNA2 | 1.22842 | 2.343102391 | 0.029 |
| DEPDC1B | 1.2235 | 2.335125356 | 0.031 |
| ARHGEF39 | 1.22249 | 2.333491157 | 0.015 |
| IQGAP3 | 1.21965 | 2.328902109 | 0.009 |
| SGOL1 | 1.2151 | 2.321568742 | 0.017 |
| ANLN | 1.21252 | 2.317420745 | 0.018 |
| TROAP | 1.1994 | 2.296441448 | 0.033 |
| BUB1B | 1.19766 | 2.293673435 | 0.02 |
| TICRR | 1.1954 | 2.290083179 | 0.023 |
| KIF14 | 1.19323 | 2.286641187 | 0.026 |
| CHEK2 | 1.18705 | 2.276866967 | 0.012 |
| QPRT | 1.18682 | 2.276504009 | 0.016 |
| RACGAP1 | 1.1801 | 2.265924827 | 0.013 |
| CASC5 | 1.17846 | 2.263350475 | 0.03 |
| TFPI | 1.17797 | 2.262581876 | 0.01 |
| FAM72D | 1.17627 | 2.259917333 | 0.027 |
| CDCA2 | 1.1759 | 2.259337818 | 0.037 |
| ZWINT | 1.17543 | 2.258601893 | 0.011 |
| STK17B | 1.17465 | 2.257381099 | 0.012 |
| ERCC6L | 1.16796 | 2.246937507 | 0.037 |
| MR1 | 1.16622 | 2.244229163 | 0.014 |
| PLK4 | 1.16538 | 2.242922855 | 0.011 |
| PRC1 | 1.16485 | 2.242099028 | 0.023 |
| NUF2 | 1.16262 | 2.238636052 | 0.039 |
| GSN | 1.16043 | 2.235240398 | 0.03 |
| ITPR1 | 1.15494 | 2.226750627 | 0 |
| POC1A | 1.15075 | 2.220292886 | 0.018 |
| SPC24 | 1.14603 | 2.213040725 | 0.033 |
| EHD2 | 1.13685 | 2.199003652 | 0.024 |
| KIAA0101 | 1.13298 | 2.193112774 | 0.022 |
| TK1 | 1.12765 | 2.185025323 | 0.044 |
| MYBL2 | 1.12338 | 2.178567781 | 0.018 |
| RP11-798M19.6 | 1.11133 | 2.160447241 | 0.004 |
| BUB1 | 1.11037 | 2.159010111 | 0.036 |
| KIF4A | 1.10995 | 2.158381668 | 0.041 |
| TRIP13 | 1.108 | 2.15546629 | 0.033 |
| ASF1B | 1.09873 | 2.141660798 | 0.023 |
| RP11-303E16.2 | 1.09708 | 2.139212796 | 0.029 |
| ECT2 | 1.08826 | 2.12617449 | 0.03 |
| MKI67 | 1.08544 | 2.122022569 | 0.04 |
| C21orf58 | 1.07862 | 2.112014881 | 0.01 |
| UBE2T | 1.07058 | 2.100277562 | 0.028 |
| RHEBL1 | 1.06516 | 2.092401922 | 0.018 |
| CCDC34 | 1.06108 | 2.086492884 | 0.044 |
| CIT | 1.0531 | 2.074983695 | 0.03 |
| STAC2 | 1.04982 | 2.070271531 | 0.035 |
| MASTL | 1.0443 | 2.06236545 | 0.013 |
| CDCA4 | 1.0414 | 2.058223998 | 0.009 |
| KIAA1524 | 1.03823 | 2.053706475 | 0.022 |
| MYOZ3 | 1.03569 | 2.050093913 | 0.021 |
| FANCD2 | 1.0351 | 2.049255685 | 0.008 |
| CENPL | 1.03079 | 2.043142742 | 0.027 |
| FANCI | 1.02495 | 2.034888859 | 0.028 |
| SSH3 | 1.02287 | 2.03195718 | 0.023 |
| NOSTRIN | 1.02277 | 2.03181634 | 0.013 |
| NECAB1 | 1.01834 | 2.025586929 | 0.034 |
| TPX2 | 1.01311 | 2.018257146 | 0.05 |
| C4orf47 | 1.0064 | 2.008891992 | 0.009 |
| SFXN3 | 1.00523 | 2.007263477 | 0.002 |
| FBXO5 | 0.99757 | 1.99663414 | 0.027 |
| H1F0 | 0.99664 | 1.995347471 | 0.045 |
| FAM111A | 0.99486 | 1.992887125 | 0.028 |
| PPP1R3G | 0.99455 | 1.992458948 | 0.022 |
| HSPA2 | 0.98684 | 1.981839321 | 0.022 |
| DERL3 | 0.98271 | 1.976174027 | 0.045 |
| MND1 | 0.98228 | 1.97558511 | 0.027 |
| C18orf56 | 0.97094 | 1.96011731 | 0.048 |
| EXO1 | 0.96351 | 1.950048492 | 0.027 |
| KIF22 | 0.96312 | 1.949521412 | 0.013 |
| HMGB2 | 0.9609 | 1.946523821 | 0.028 |
| IL17RC | 0.94685 | 1.927659182 | 0.014 |
| NCAPD2 | 0.94392 | 1.923748231 | 0.025 |
| NCAPG2 | 0.93949 | 1.917850149 | 0.039 |
| TRAIP | 0.93847 | 1.916494689 | 0.038 |
| ATAD2 | 0.93027 | 1.905632601 | 0.013 |
| C4orf21 | 0.92952 | 1.904642196 | 0.005 |
| POLQ | 0.92931 | 1.904364975 | 0.004 |
| AUNIP | 0.92558 | 1.899447715 | 0.022 |
| TYMS | 0.92201 | 1.894753275 | 0.043 |
| SUV39H1 | 0.91433 | 1.884693601 | 0.021 |
| CENPU | 0.91113 | 1.880517849 | 0.034 |
| BRIP1 | 0.90509 | 1.872661314 | 0.029 |
| TMEM194A | 0.90228 | 1.8690174 | 0.034 |
| TCF19 | 0.90222 | 1.868939671 | 0.044 |
| HSPB6 | 0.89618 | 1.861131508 | 0.021 |
| GPC6 | 0.89615 | 1.861092807 | 0.009 |
| RP1-152L7.5 | 0.88594 | 1.847968288 | 0.004 |
| SLC16A3 | 0.86835 | 1.82557381 | 0.045 |
| C16orf59 | 0.86644 | 1.823158512 | 0.034 |
| WDR90 | 0.86266 | 1.818387914 | 0.003 |
| THAP10 | 0.86021 | 1.815302528 | 0.006 |
| ORC6 | 0.85826 | 1.812850556 | 0.033 |
| ELL2 | 0.85646 | 1.810590136 | 0.01 |
| SAP30 | 0.83607 | 1.785180558 | 0.001 |
| TDP1 | 0.83025 | 1.777993438 | 0.019 |
| RAD51 | 0.82446 | 1.770872078 | 0.026 |
| HILPDA | 0.81328 | 1.757201943 | 0.017 |
| DHRS4 | 0.81053 | 1.753855635 | 0.009 |
| TMEM237 | 0.78959 | 1.728583146 | 0.006 |
| H2AFJ | 0.78951 | 1.728487296 | 0.041 |
| ZNF850 | 0.78732 | 1.725865456 | 0.047 |
| TMEM106C | 0.78586 | 1.724119772 | 0.02 |
| RRM1 | 0.78509 | 1.723199815 | 0.019 |
| PLOD2 | 0.78319 | 1.72093189 | 0.025 |
| CENPO | 0.77774 | 1.714443071 | 0.047 |
| LIG1 | 0.7766 | 1.713088875 | 0.003 |
| CEP135 | 0.77537 | 1.711628967 | 0.044 |
| CEP112 | 0.77437 | 1.710442967 | 0.02 |
| RNF26 | 0.77436 | 1.710431112 | 0.023 |
| CENPQ | 0.77234 | 1.708037915 | 0.023 |
| PPP1R3C | 0.75734 | 1.690371092 | 0.027 |
| LRIG3 | 0.74696 | 1.678252743 | 0.029 |
| KNTC1 | 0.74499 | 1.675962652 | 0.021 |
| MIR210HG | 0.74329 | 1.673988945 | 0.017 |
| NRM | 0.74301 | 1.673664086 | 0.037 |
| GINS1 | 0.7429 | 1.673536481 | 0.042 |
| POLA2 | 0.73259 | 1.66161944 | 0.038 |
| ENOX1 | 0.73038 | 1.659076028 | 0.01 |
| TRAM2 | 0.72356 | 1.651251646 | 0.004 |
| FAM162A | 0.72317 | 1.650805328 | 0.001 |
| RAD54L | 0.71956 | 1.646679746 | 0.034 |
| CD99 | 0.7066 | 1.631953556 | 0.028 |
| SASS6 | 0.70536 | 1.63055149 | 0.014 |
| N6AMT2 | 0.70222 | 1.627006483 | 0.011 |
| LHPP | 0.69801 | 1.622265556 | 0.026 |
| ZNF331 | 0.69076 | 1.614133607 | 0.046 |
| KIF16B | 0.68175 | 1.604084342 | 0.017 |
| PDLIM3 | 0.66759 | 1.588417323 | 0.044 |
| ERI2 | 0.66738 | 1.588186129 | 0.035 |
| SPIN4 | 0.66677 | 1.587514754 | 0.028 |
| TRIM2 | 0.66328 | 1.583679064 | 0.039 |
| ZMYM1 | 0.66243 | 1.582746275 | 0.045 |
| RNASEH2A | 0.65931 | 1.579327096 | 0.041 |
| RAD54B | 0.65664 | 1.576406934 | 0.036 |
| MLLT3 | 0.6532 | 1.572652585 | 0.02 |
| LDLRAP1 | 0.64908 | 1.568167863 | 0.041 |
| BUB3 | 0.64103 | 1.559442113 | 0.037 |
| SLC25A27 | 0.62916 | 1.546664196 | 0.006 |
| UCP2 | 0.59674 | 1.512295429 | 0.029 |
| GBE1 | 0.58295 | 1.49790902 | 0.028 |
| SUMF2 | 0.53688 | 1.450831526 | 0.037 |
| VEGFB | 0.53222 | 1.446152805 | 0.026 |
| JADE1 | 0.52653 | 1.4404604 | 0.021 |
| LBR | 0.51881 | 1.432772945 | 0.007 |
| EFHC1 | 0.50702 | 1.421111744 | 0.027 |
| P4HA1 | 0.50627 | 1.420373156 | 0.044 |
| IGFBP2 | 0.49969 | 1.413909715 | 0.034 |
| KIAA1715 | 0.47372 | 1.388685592 | 0.027 |

**Supplementary Table 3. Genes upregulated in Notch^hi^ vs. DN cells (103 genes, P<0.05)**

| **gene ID** | **log2 change** | **fold change** | **P value** |
| --- | --- | --- | --- |
| FAM107A | 2.39048 | 5.243317833 | 0.004 |
| CYP17A1 | 2.17117 | 4.503885029 | 0.013 |
| VCAM1 | 2.10947 | 4.315327342 | 0.009 |
| OSTCP6 | 1.94637 | 3.854035873 | 0.026 |
| CASP4 | 1.93517 | 3.824231872 | 0.003 |
| IL6 | 1.89952 | 3.730890453 | 0.03 |
| ISLR | 1.88485 | 3.693145239 | 0.031 |
| CCDC89 | 1.85464 | 3.616614933 | 0.001 |
| RP11-146F11.4 | 1.82996 | 3.55527215 | 0.032 |
| FAM198B | 1.79374 | 3.467125358 | 0.019 |
| WFDC1 | 1.78559 | 3.447594268 | 0.008 |
| RP11-431M7.3 | 1.76558 | 3.400106651 | 0.004 |
| TNFAIP8L3 | 1.74699 | 3.356575282 | 0.018 |
| CXorf64 | 1.74429 | 3.350299335 | 0.043 |
| AQP4 | 1.71985 | 3.294021565 | 0.011 |
| RP11-45A16.4 | 1.71674 | 3.286928331 | 0.034 |
| SERPINB10 | 1.70878 | 3.268842802 | 0.047 |
| COL9A1 | 1.61234 | 3.057473512 | 0.026 |
| KAL1 | 1.60426 | 3.040397599 | 0.02 |
| GALNT15 | 1.59209 | 3.01485789 | 0.014 |
| PIF1 | 1.54103 | 2.910021879 | 0.01 |
| CCL2 | 1.51494 | 2.857869422 | 0.045 |
| GS1-24F4.2 | 1.5099 | 2.847902983 | 0.042 |
| CYP19A1 | 1.47913 | 2.787805673 | 0.049 |
| CDCA3 | 1.44698 | 2.726367419 | 0.013 |
| CDK1 | 1.44695 | 2.726310726 | 0.015 |
| CDC25C | 1.38226 | 2.606764043 | 0.017 |
| RRM2P3 | 1.37795 | 2.598988049 | 0.048 |
| RPE65 | 1.3746 | 2.592960088 | 0.028 |
| ESPL1 | 1.36719 | 2.579676217 | 0.01 |
| NDC80 | 1.3658 | 2.577191962 | 0.02 |
| NEK2 | 1.35726 | 2.561981397 | 0.018 |
| KIF23 | 1.35689 | 2.561324424 | 0.018 |
| FAM83D | 1.35465 | 2.55735067 | 0.046 |
| CASP1 | 1.34907 | 2.547478552 | 0.05 |
| MXD3 | 1.32785 | 2.51028297 | 0.045 |
| PLK1 | 1.31653 | 2.490663293 | 0.022 |
| RP11-1000B6.2 | 1.31197 | 2.482803354 | 0.04 |
| CCNA2 | 1.30034 | 2.462869183 | 0.016 |
| CENPA | 1.29427 | 2.452528667 | 0.029 |
| TTK | 1.29358 | 2.451355973 | 0.015 |
| MR1 | 1.28342 | 2.434153249 | 0.007 |
| ATP5G1P4 | 1.24498 | 2.370152684 | 0.031 |
| NEIL3 | 1.24131 | 2.364131033 | 0.027 |
| SPC25 | 1.22437 | 2.33653395 | 0.031 |
| KIF2C | 1.21733 | 2.325160008 | 0.027 |
| C10orf90 | 1.19418 | 2.288147413 | 0.021 |
| SHCBP1 | 1.19019 | 2.281827924 | 0.024 |
| IFI35 | 1.1824 | 2.269540133 | 0.013 |
| STK17B | 1.17412 | 2.256551962 | 0.012 |
| IQGAP3 | 1.17325 | 2.255191585 | 0.012 |
| A2M | 1.16565 | 2.243342657 | 0.043 |
| KIF18B | 1.16322 | 2.239567268 | 0.037 |
| CEP55 | 1.15827 | 2.231896304 | 0.042 |
| NCAPH | 1.15716 | 2.230179759 | 0.028 |
| KIF11 | 1.14512 | 2.211645258 | 0.038 |
| MTFR2 | 1.14505 | 2.211537951 | 0.039 |
| UBE2C | 1.14363 | 2.209362274 | 0.036 |
| CDCA8 | 1.14164 | 2.206316863 | 0.022 |
| SKA1 | 1.14073 | 2.204925636 | 0.021 |
| ARHGAP11B | 1.13663 | 2.198668346 | 0.025 |
| FAM72D | 1.13414 | 2.194876857 | 0.033 |
| ARHGAP11A | 1.13307 | 2.193249592 | 0.027 |
| DEPDC1 | 1.13274 | 2.192747968 | 0.039 |
| TACC3 | 1.12513 | 2.181212004 | 0.028 |
| CYP2J2 | 1.1192 | 2.172264832 | 0.023 |
| CKAP2L | 1.11548 | 2.166670846 | 0.038 |
| KIFC1 | 1.11257 | 2.162304949 | 0.033 |
| ANLN | 1.11039 | 2.159040042 | 0.03 |
| CDCA2 | 1.10739 | 2.154555109 | 0.049 |
| RRM2 | 1.10594 | 2.152390732 | 0.044 |
| NUSAP1 | 1.08396 | 2.119846791 | 0.036 |
| CHEK2 | 1.08138 | 2.116059215 | 0.022 |
| ARHGEF39 | 1.08026 | 2.114417103 | 0.032 |
| E2F8 | 1.07966 | 2.113537925 | 0.043 |
| NCAPG | 1.07806 | 2.111195236 | 0.029 |
| AMDHD2 | 1.05491 | 2.077588596 | 0.014 |
| KIF14 | 1.05219 | 2.073675283 | 0.049 |
| BUB1B | 1.04129 | 2.058067072 | 0.043 |
| RACGAP1 | 1.02708 | 2.037895395 | 0.031 |
| POC1A | 1.00456 | 2.006331503 | 0.039 |
| TFPI | 0.97921 | 1.97138561 | 0.033 |
| CDCA5 | 0.97843 | 1.970320059 | 0.021 |
| CENPL | 0.97263 | 1.962414774 | 0.036 |
| PLK4 | 0.95496 | 1.938525886 | 0.038 |
| TRAIP | 0.93481 | 1.91163886 | 0.039 |
| C21orf58 | 0.9111 | 1.880478745 | 0.029 |
| KIAA1524 | 0.89287 | 1.856866376 | 0.05 |
| NOSTRIN | 0.83912 | 1.788958599 | 0.041 |
| RP11-303E16.3 | 0.82338 | 1.769546901 | 0.048 |
| RP11-340F14.5 | 0.81062 | 1.753965049 | 0.028 |
| KIF22 | 0.79396 | 1.733827054 | 0.041 |
| FANCD2 | 0.79105 | 1.730333349 | 0.043 |
| SUV39H1 | 0.78231 | 1.719882494 | 0.048 |
| ENOX1 | 0.70225 | 1.627040316 | 0.013 |
| PDLIM3 | 0.68945 | 1.612668602 | 0.037 |
| POLQ | 0.67495 | 1.59654144 | 0.039 |
| BCL2L12 | 0.66465 | 1.585183659 | 0.021 |
| IFI6 | 0.61772 | 1.53444826 | 0.022 |
| LMCD1 | 0.57041 | 1.484945518 | 0.048 |
| LIG1 | 0.54647 | 1.460507736 | 0.036 |
| SLC25A27 | 0.51119 | 1.425225301 | 0.024 |
| C15orf39 | 0.49724 | 1.411510636 | 0.034 |

**Supplementary Table 4. Gene upregulated in both CD133^hi^ and Notch^hi^ vs. DN cells (72 genes, P<0.05)**

|  | **CD133^hi^ vs. DN cells** | | | **Notch^hi^ vs. DN cells** | | |
| --- | --- | --- | --- | --- | --- | --- |
| **gene ID** | **log2 change** | **fold change** | **P value** | **log2 change** | **fold change** | **P value** |
| FAM107A | 2.50757 | 5.686614476 | 0.002 | 2.39048 | 5.243317833 | 0.004 |
| ISLR | 1.92006 | 3.784387972 | 0.028 | 1.88485 | 3.693145239 | 0.031 |
| VCAM1 | 1.90117 | 3.735159886 | 0.019 | 2.10947 | 4.315327342 | 0.009 |
| CCDC89 | 1.84612 | 3.595319544 | 0.001 | 1.85464 | 3.616614933 | 0.001 |
| CDK1 | 1.7355 | 3.329948811 | 0.003 | 1.44695 | 2.726310726 | 0.015 |
| CDCA3 | 1.7025 | 3.254644571 | 0.003 | 1.44698 | 2.726367419 | 0.013 |
| COL9A1 | 1.66998 | 3.182101821 | 0.021 | 1.61234 | 3.057473512 | 0.026 |
| MXD3 | 1.66387 | 3.168653706 | 0.012 | 1.32785 | 2.51028297 | 0.045 |
| KIF23 | 1.63886 | 3.114196546 | 0.004 | 1.35689 | 2.561324424 | 0.018 |
| FAM83D | 1.63365 | 3.10297054 | 0.016 | 1.35465 | 2.55735067 | 0.046 |
| NDC80 | 1.61826 | 3.07004542 | 0.006 | 1.3658 | 2.577191962 | 0.02 |
| ESPL1 | 1.61569 | 3.064581346 | 0.002 | 1.36719 | 2.579676217 | 0.01 |
| PIF1 | 1.58945 | 3.00934602 | 0.008 | 1.54103 | 2.910021879 | 0.01 |
| GALNT15 | 1.58518 | 3.000452311 | 0.014 | 1.59209 | 3.01485789 | 0.014 |
| CCNA2 | 1.57992 | 2.989532718 | 0.003 | 1.30034 | 2.462869183 | 0.016 |
| RRM2P3 | 1.5775 | 2.984522231 | 0.024 | 1.37795 | 2.598988049 | 0.048 |
| CDC25C | 1.55739 | 2.943209014 | 0.007 | 1.38226 | 2.606764043 | 0.017 |
| NEIL3 | 1.54942 | 2.926994429 | 0.006 | 1.24131 | 2.364131033 | 0.027 |
| NEK2 | 1.5119 | 2.851853752 | 0.008 | 1.35726 | 2.561981397 | 0.018 |
| TTK | 1.48886 | 2.806671078 | 0.005 | 1.29358 | 2.451355973 | 0.015 |
| KIF2C | 1.45942 | 2.749977853 | 0.008 | 1.21733 | 2.325160008 | 0.027 |
| RRM2 | 1.43805 | 2.709543859 | 0.009 | 1.10594 | 2.152390732 | 0.044 |
| E2F8 | 1.43487 | 2.703578038 | 0.007 | 1.07966 | 2.113537925 | 0.043 |
| KIF11 | 1.4316 | 2.697457071 | 0.01 | 1.14512 | 2.211645258 | 0.038 |
| SPC25 | 1.43135 | 2.696989678 | 0.012 | 1.22437 | 2.33653395 | 0.031 |
| CASP4 | 1.41738 | 2.671000044 | 0.033 | 1.93517 | 3.824231872 | 0.003 |
| KIFC1 | 1.40286 | 2.644252598 | 0.007 | 1.11257 | 2.162304949 | 0.033 |
| PLK1 | 1.38233 | 2.606890527 | 0.016 | 1.31653 | 2.490663293 | 0.022 |
| CKAP2L | 1.36901 | 2.582932604 | 0.011 | 1.11548 | 2.166670846 | 0.038 |
| ARHGAP11A | 1.3478 | 2.545237001 | 0.009 | 1.13307 | 2.193249592 | 0.027 |
| NCAPG | 1.34232 | 2.535587393 | 0.007 | 1.07806 | 2.111195236 | 0.029 |
| NUSAP1 | 1.33721 | 2.526622274 | 0.01 | 1.08396 | 2.119846791 | 0.036 |
| SHCBP1 | 1.33098 | 2.515735068 | 0.012 | 1.19019 | 2.281827924 | 0.024 |
| ARHGAP11B | 1.30345 | 2.468184086 | 0.01 | 1.13663 | 2.198668346 | 0.025 |
| SKA1 | 1.2887 | 2.443078127 | 0.009 | 1.14073 | 2.204925636 | 0.021 |
| DEPDC1 | 1.28459 | 2.436128105 | 0.02 | 1.13274 | 2.192747968 | 0.039 |
| NCAPH | 1.27529 | 2.420474676 | 0.016 | 1.15716 | 2.230179759 | 0.028 |
| CDCA8 | 1.27231 | 2.415480155 | 0.011 | 1.14164 | 2.206316863 | 0.022 |
| KIF18B | 1.2691 | 2.41011168 | 0.023 | 1.16322 | 2.239567268 | 0.037 |
| CENPA | 1.26118 | 2.396917079 | 0.034 | 1.29427 | 2.452528667 | 0.029 |
| UBE2C | 1.25681 | 2.389667675 | 0.021 | 1.14363 | 2.209362274 | 0.036 |
| CDCA5 | 1.25237 | 2.382324602 | 0.003 | 0.97843 | 1.970320059 | 0.021 |
| TACC3 | 1.24222 | 2.365622712 | 0.015 | 1.12513 | 2.181212004 | 0.028 |
| MTFR2 | 1.23771 | 2.358239101 | 0.026 | 1.14505 | 2.211537951 | 0.039 |
| CEP55 | 1.23722 | 2.35743828 | 0.03 | 1.15827 | 2.231896304 | 0.042 |
| ARHGEF39 | 1.22249 | 2.333491157 | 0.015 | 1.08026 | 2.114417103 | 0.032 |
| IQGAP3 | 1.21965 | 2.328902109 | 0.009 | 1.17325 | 2.255191585 | 0.012 |
| ANLN | 1.21252 | 2.317420745 | 0.018 | 1.11039 | 2.159040042 | 0.03 |
| BUB1B | 1.19766 | 2.293673435 | 0.02 | 1.04129 | 2.058067072 | 0.043 |
| KIF14 | 1.19323 | 2.286641187 | 0.026 | 1.05219 | 2.073675283 | 0.049 |
| CHEK2 | 1.18705 | 2.276866967 | 0.012 | 1.08138 | 2.116059215 | 0.022 |
| RACGAP1 | 1.1801 | 2.265924827 | 0.013 | 1.02708 | 2.037895395 | 0.031 |
| TFPI | 1.17797 | 2.262581876 | 0.01 | 0.97921 | 1.97138561 | 0.033 |
| FAM72D | 1.17627 | 2.259917333 | 0.027 | 1.13414 | 2.194876857 | 0.033 |
| CDCA2 | 1.1759 | 2.259337818 | 0.037 | 1.10739 | 2.154555109 | 0.049 |
| STK17B | 1.17465 | 2.257381099 | 0.012 | 1.17412 | 2.256551962 | 0.012 |
| MR1 | 1.16622 | 2.244229163 | 0.014 | 1.28342 | 2.434153249 | 0.007 |
| PLK4 | 1.16538 | 2.242922855 | 0.011 | 0.95496 | 1.938525886 | 0.038 |
| POC1A | 1.15075 | 2.220292886 | 0.018 | 1.00456 | 2.006331503 | 0.039 |
| C21orf58 | 1.07862 | 2.112014881 | 0.01 | 0.9111 | 1.880478745 | 0.029 |
| KIAA1524 | 1.03823 | 2.053706475 | 0.022 | 0.89287 | 1.856866376 | 0.05 |
| FANCD2 | 1.0351 | 2.049255685 | 0.008 | 0.79105 | 1.730333349 | 0.043 |
| CENPL | 1.03079 | 2.043142742 | 0.027 | 0.97263 | 1.962414774 | 0.036 |
| NOSTRIN | 1.02277 | 2.03181634 | 0.013 | 0.83912 | 1.788958599 | 0.041 |
| KIF22 | 0.96312 | 1.949521412 | 0.013 | 0.79396 | 1.733827054 | 0.041 |
| TRAIP | 0.93847 | 1.916494689 | 0.038 | 0.93481 | 1.91163886 | 0.039 |
| POLQ | 0.92931 | 1.904364975 | 0.004 | 0.67495 | 1.59654144 | 0.039 |
| SUV39H1 | 0.91433 | 1.884693601 | 0.021 | 0.78231 | 1.719882494 | 0.048 |
| LIG1 | 0.7766 | 1.713088875 | 0.003 | 0.54647 | 1.460507736 | 0.036 |
| ENOX1 | 0.73038 | 1.659076028 | 0.01 | 0.70225 | 1.627040316 | 0.013 |
| PDLIM3 | 0.66759 | 1.588417323 | 0.044 | 0.68945 | 1.612668602 | 0.037 |
| SLC25A27 | 0.62916 | 1.546664196 | 0.006 | 0.51119 | 1.425225301 | 0.024 |

**Supplementary Table 5. Genes downregulated in CD133^hi^ vs. DN cells (119 genes, P<0.05)**

| **gene ID** | **log2 Change** | **fold change** | **P value** |
| --- | --- | --- | --- |
| WDR65 | 2.75879 | 6.768283504 | 0.002 |
| XKR7 | 2.58006 | 5.979645676 | 0.003 |
| HIST1H4E | 2.47299 | 5.551932393 | 0.002 |
| HIST1H4D | 2.44739 | 5.454284672 | 0.002 |
| GSX1 | 2.28776 | 4.882973674 | 0.001 |
| HIST1H2BE | 2.25294 | 4.76653206 | 0.005 |
| GPR52 | 2.20557 | 4.612567424 | 0.011 |
| LRRTM1 | 2.17756 | 4.523877927 | 0.001 |
| RP11-158G18.1 | 2.13848 | 4.402979112 | 0.015 |
| GAPDHP55 | 2.07727 | 4.220078981 | 0.002 |
| FCGR1A | 2.04919 | 4.138735353 | 0.019 |
| HIST1H4H | 2.03203 | 4.089799167 | 0.017 |
| RP11-6L6.7 | 2.01784 | 4.049770071 | 0.01 |
| HIST1H2BF | 2.00786 | 4.02185202 | 0.012 |
| HIST1H1T | 1.95989 | 3.890323156 | 0.018 |
| MIR7-3HG | 1.95027 | 3.864468482 | 0.026 |
| RP1-34B20.4 | 1.89263 | 3.713115005 | 0.006 |
| HIST1H2BG | 1.88389 | 3.690688559 | 0.025 |
| RP11-76E16.2 | 1.88091 | 3.68307302 | 0.025 |
| RP11-104O19.2 | 1.87704 | 3.673206491 | 0.011 |
| AL139333.1 | 1.87416 | 3.665881115 | 0.032 |
| RP11-648M2.2 | 1.8644 | 3.641164702 | 0.033 |
| SLCO2A1 | 1.8589 | 3.627309881 | 0.017 |
| S100A7 | 1.83797 | 3.575066307 | 0.035 |
| AC006355.3 | 1.81949 | 3.529564046 | 0.036 |
| RP11-295P9.12 | 1.80579 | 3.496205534 | 0.025 |
| HIST1H2BC | 1.79611 | 3.47282569 | 0.011 |
| SNAP23P | 1.78332 | 3.442173936 | 0.037 |
| RP11-481C4.1 | 1.77376 | 3.419439822 | 0.041 |
| RBMXP4 | 1.7643 | 3.397091318 | 0.039 |
| RNU4-8P | 1.76291 | 3.393819883 | 0.042 |
| AC009302.4 | 1.75562 | 3.37671402 | 0.038 |
| TMSB4XP3 | 1.74955 | 3.362536668 | 0.042 |
| PCLO | 1.7472 | 3.357063904 | 0.023 |
| PIP | 1.73656 | 3.332396344 | 0.043 |
| CTD-2199O4.1 | 1.73263 | 3.323331021 | 0.04 |
| RP11-619L19.1 | 1.72222 | 3.299437295 | 0.046 |
| S100A3 | 1.71627 | 3.285857693 | 0.046 |
| RPL5P14 | 1.70841 | 3.268004568 | 0.049 |
| RP4-609E1.2 | 1.70156 | 3.252524671 | 0.047 |
| GPR85 | 1.69393 | 3.235368409 | 0.045 |
| RP11-258F1.1 | 1.69127 | 3.229408624 | 0.038 |
| HIST1H1PS1 | 1.6722 | 3.187002167 | 0.039 |
| MIR647 | 1.66221 | 3.165009873 | 0.043 |
| RP11-97N19.2 | 1.64329 | 3.123773825 | 0.049 |
| HECW1 | 1.61511 | 3.063349554 | 0.039 |
| FRY-AS1 | 1.61489 | 3.062882452 | 0.014 |
| RP11-66D17.5 | 1.59934 | 3.030046639 | 0.043 |
| KIAA1683 | 1.56139 | 2.951380645 | 0.025 |
| CTD-2072I24.1 | 1.51518 | 2.858344883 | 0.027 |
| RP11-266L9.1 | 1.50414 | 2.836555319 | 0.021 |
| RP11-1029F8.1 | 1.48131 | 2.792021401 | 0.039 |
| RPS6KL1 | 1.47823 | 2.786067091 | 0.02 |
| RP11-649A18.5 | 1.47165 | 2.773389028 | 0.028 |
| AC003682.16 | 1.47067 | 2.771505749 | 0.046 |
| LAMC2 | 1.46015 | 2.751369687 | 0.05 |
| OR6V1 | 1.44955 | 2.731228466 | 0.047 |
| PROX2 | 1.4352 | 2.704196521 | 0.006 |
| RP11-196G18.22 | 1.42098 | 2.677673393 | 0.036 |
| LAPTM5 | 1.42032 | 2.676448699 | 0.048 |
| AC012513.4 | 1.40218 | 2.643006549 | 0.014 |
| CDS1 | 1.37434 | 2.592492832 | 0.013 |
| AC008498.1 | 1.35495 | 2.557882512 | 0.044 |
| RN7SL200P | 1.35107 | 2.551012556 | 0.032 |
| TPTE2 | 1.33116 | 2.516048967 | 0.032 |
| CAMSAP3 | 1.30944 | 2.478453171 | 0.02 |
| HIST1H2AC | 1.30588 | 2.472344869 | 0.048 |
| RXFP4 | 1.28877 | 2.443196668 | 0.043 |
| SHH | 1.27946 | 2.427480994 | 0.014 |
| KCND3-AS1 | 1.27781 | 2.424706288 | 0.02 |
| KIAA1755 | 1.27171 | 2.414475794 | 0.043 |
| HIST2H2BE | 1.26939 | 2.410596192 | 0.045 |
| RN7SKP16 | 1.25765 | 2.391059449 | 0.028 |
| CACNA2D2 | 1.25023 | 2.378793436 | 0.024 |
| RP11-385J1.2 | 1.23616 | 2.355706821 | 0.039 |
| HIST1H4I | 1.23553 | 2.354678349 | 0.022 |
| MIR29A | 1.2253 | 2.338040628 | 0.02 |
| GRIK3 | 1.21444 | 2.32050692 | 0.009 |
| TAS2R9 | 1.19259 | 2.285627025 | 0.005 |
| PRSS37 | 1.18042 | 2.266427481 | 0.049 |
| AC092431.2 | 1.17868 | 2.263695645 | 0.008 |
| RP11-366L5.1 | 1.17046 | 2.250834528 | 0.04 |
| PIK3AP1 | 1.16329 | 2.239675935 | 0.047 |
| RP11-49O14.2 | 1.13724 | 2.199598183 | 0.038 |
| RN7SL832P | 1.12975 | 2.188208182 | 0.003 |
| DGKI | 1.12323 | 2.178341283 | 0.034 |
| HIST1H2AG | 1.08728 | 2.124730703 | 0.047 |
| PCDHGA10 | 1.08572 | 2.122434453 | 0.019 |
| RYKP1 | 1.08002 | 2.114065388 | 0.045 |
| CTD-2165H16.4 | 1.0378 | 2.053094452 | 0.039 |
| FAM65C | 1.02459 | 2.034381151 | 0.042 |
| RP11-506H20.1 | 1.02199 | 2.030718126 | 0.039 |
| SLC22A4 | 0.99695 | 1.995776268 | 0.012 |
| WI2-89927D4.1 | 0.97264 | 1.962428376 | 0.049 |
| RP11-582J16.4 | 0.96752 | 1.955476231 | 0.017 |
| AC016586.1 | 0.96622 | 1.953714962 | 0.038 |
| MEGF11 | 0.96019 | 1.945566105 | 0.007 |
| SNORD64 | 0.9554 | 1.939117197 | 0.048 |
| RP6-109B7.2 | 0.95253 | 1.935263483 | 0.04 |
| CTB-119C2.1 | 0.95096 | 1.933158595 | 0.021 |
| AOC2 | 0.92471 | 1.898302621 | 0.03 |
| DCAF4L1 | 0.90869 | 1.877340057 | 0.017 |
| SLC12A5 | 0.82959 | 1.777180233 | 0.044 |
| AC144652.1 | 0.79281 | 1.732445538 | 0.017 |
| CTD-3131K8.3 | 0.78643 | 1.724801096 | 0.046 |
| FAM222A | 0.78149 | 1.718905223 | 0.023 |
| MIR17HG | 0.76552 | 1.699982624 | 0.038 |
| KBTBD8 | 0.76445 | 1.698722269 | 0.009 |
| C20orf112 | 0.76257 | 1.696510078 | 0.004 |
| HM13-IT1 | 0.76039 | 1.693948484 | 0.047 |
| KIAA1024 | 0.75734 | 1.690371092 | 0.05 |
| RP11-138A9.2 | 0.74947 | 1.681175107 | 0.01 |
| RP3-368A4.5 | 0.74493 | 1.675892953 | 0.036 |
| HELZ2 | 0.71586 | 1.642462008 | 0.026 |
| ZNF697 | 0.62897 | 1.546460517 | 0.022 |
| TMEM150A | 0.60459 | 1.520546567 | 0.028 |
| GDF11 | 0.51765 | 1.431621386 | 0.046 |
| HCG18 | 0.51026 | 1.424306859 | 0.031 |
| ZNF74 | 0.45438 | 1.370193836 | 0.04 |

**Supplementary Table 6. Genes downregulated in Notch^hi^ vs. DN cells (40 genes, P<0.05)**

| **gene ID** | **log2 change** | **fold change** | **P value** |
| --- | --- | --- | --- |
| AC073089.1 | 1.91489 | 3.77085062 | 0.025 |
| AL139333.1 | 1.86229 | 3.635843243 | 0.033 |
| S100A7 | 1.82738 | 3.548919869 | 0.036 |
| RP11-325L12.5 | 1.80023 | 3.482757444 | 0.031 |
| MTND4P10 | 1.78007 | 3.434428381 | 0.041 |
| MAGI1-IT1 | 1.77705 | 3.427246596 | 0.039 |
| CCT7P1 | 1.61238 | 3.057558284 | 0.046 |
| SLC25A2 | 1.61193 | 3.056604731 | 0.036 |
| AC064852.5 | 1.59487 | 3.020672969 | 0.029 |
| AC005082.12 | 1.59453 | 3.01996117 | 0.039 |
| OR6V1 | 1.57593 | 2.981276118 | 0.03 |
| RP11-650J17.2 | 1.57335 | 2.975949407 | 0.045 |
| ANKRD44-IT1 | 1.48781 | 2.804629113 | 0.022 |
| AC009061.1 | 1.48486 | 2.798900111 | 0.05 |
| AC009505.2 | 1.47428 | 2.778449464 | 0.01 |
| HSD11B2 | 1.44707 | 2.726537504 | 0.038 |
| RXFP4 | 1.43327 | 2.700581336 | 0.024 |
| SNORA28 | 1.42825 | 2.691200726 | 0.032 |
| MIAT | 1.42043 | 2.676652776 | 0.048 |
| IGSF6 | 1.36087 | 2.568400171 | 0.038 |
| CTD-2525I3.5 | 1.33515 | 2.523017127 | 0.03 |
| RP11-563J2.2 | 1.33502 | 2.522789791 | 0.03 |
| RP11-274H2.2 | 1.32814 | 2.510787619 | 0.045 |
| RP5-1099D15.1 | 1.32229 | 2.500627212 | 0.021 |
| FAM222A-AS1 | 1.30649 | 2.473390447 | 0.038 |
| PCDHGB3 | 1.28524 | 2.437225939 | 0.031 |
| LINC00643 | 1.257 | 2.38998241 | 0.04 |
| TMPRSS11A | 1.18149 | 2.26810904 | 0.032 |
| SNORD64 | 1.16366 | 2.240250406 | 0.016 |
| CAMSAP3 | 1.1491 | 2.217755005 | 0.038 |
| CTC-412M14.6 | 1.06023 | 2.085263936 | 0.033 |
| RP11-91I20.3 | 1.01026 | 2.014274076 | 0.048 |
| MIR17HG | 0.86067 | 1.815881426 | 0.019 |
| TAS2R9 | 0.8265 | 1.773377898 | 0.047 |
| RP11-421E14.2 | 0.77335 | 1.709234094 | 0.043 |
| MAK | 0.71929 | 1.646371599 | 0.029 |
| TAS2R18 | 0.68994 | 1.613216425 | 0.038 |
| C20orf112 | 0.59287 | 1.508244164 | 0.026 |
| EPHA4 | 0.58597 | 1.501047884 | 0.019 |
| UBN2 | 0.55584 | 1.470024296 | 0.029 |

**Supplementary Table 7. Genes downregulated in both CD133^hi^ and Notch^hi^ vs. DN cells (9 genes, P<0.05)**

|  | **CD133^hi^ vs. DN cells** | | | **Notch^hi^ vs. DN cells** | | |
| --- | --- | --- | --- | --- | --- | --- |
| **gene ID** | **log2 change** | **fold change** | **P value** | **log2 change** | **fold change** | **P value** |
| AL139333.1 | 1.87416 | 3.665881115 | 0.032 | 1.86229 | 3.635843243 | 0.033 |
| S100A7 | 1.83797 | 3.575066307 | 0.035 | 1.82738 | 3.548919869 | 0.036 |
| OR6V1 | 1.44955 | 2.731228466 | 0.047 | 1.57593 | 2.981276118 | 0.03 |
| CAMSAP3 | 1.30944 | 2.478453171 | 0.02 | 1.1491 | 2.217755005 | 0.038 |
| RXFP4 | 1.28877 | 2.443196668 | 0.043 | 1.43327 | 2.700581336 | 0.024 |
| TAS2R9 | 1.19259 | 2.285627025 | 0.005 | 0.8265 | 1.773377898 | 0.047 |
| SNORD64 | 0.9554 | 1.939117197 | 0.048 | 1.16366 | 2.240250406 | 0.016 |
| MIR17HG | 0.76552 | 1.699982624 | 0.038 | 0.86067 | 1.815881426 | 0.019 |
| C20orf112 | 0.76257 | 1.696510078 | 0.004 | 0.59287 | 1.508244164 | 0.026 |

**Supplementary Table 8. Gene Set Enrichment Analysis on CD133^hi^ and Notch^hi^ transcriptomes: gene sets enriched in CD133^hi^ cells are shown (FDR<0.001, top 100 gene sets). Hypoxia-related gene sets are highlighted in green.**

| **gene set** | **size** | **NES** | **FDR** | **Nominal P value** |
| --- | --- | --- | --- | --- |
| PUJANA_BRCA2_PCC_NETWORK | 412 | 9.281712 | 0 | 0 |
| ZHANG_TLX_TARGETS_60HR_DN | 268 | 9.09273 | 0 | 0 |
| DUTERTRE_ESTRADIOL_RESPONSE_24HR_UP | 313 | 9.035122 | 0 | 0 |
| HALLMARK_E2F_TARGETS | 198 | 8.946267 | 0 | 0 |
| SHEDDEN_LUNG_CANCER_POOR_SURVIVAL_A6 | 437 | 8.887847 | 0 | 0 |
| BLUM_RESPONSE_TO_SALIRASIB_DN | 341 | 8.570708 | 0 | 0 |
| KOBAYASHI_EGFR_SIGNALING_24HR_DN | 243 | 8.557932 | 0 | 0 |
| HALLMARK_G2M_CHECKPOINT | 199 | 8.425457 | 0 | 0 |
| ZHANG_TLX_TARGETS_36HR_DN | 184 | 8.325513 | 0 | 0 |
| RHEIN_ALL_GLUCOCORTICOID_THERAPY_DN | 353 | 8.152783 | 0 | 0 |
| ZHANG_BREAST_CANCER_PROGENITORS_UP | 410 | 8.064932 | 0 | 0 |
| PUJANA_XPRSS_INT_NETWORK | 167 | 7.994928 | 0 | 0 |
| HSIAO_HOUSEKEEPING_GENES | 388 | 7.90379 | 0 | 0 |
| WONG_EMBRYONIC_STEM_CELL_CORE | 332 | 7.636943 | 0 | 0 |
| FOURNIER_ACINAR_DEVELOPMENT_LATE_2 | 270 | 7.417406 | 0 | 0 |
| SHEN_SMARCA2_TARGETS_UP | 418 | 7.291222 | 0 | 0 |
| ROSTY_CERVICAL_CANCER_PROLIFERATION_CLUSTER | 136 | 7.2039638 | 0 | 0 |
| DACOSTA_UV_RESPONSE_VIA_ERCC3_COMMON_DN | 476 | 7.191216 | 0 | 0 |
| PYEON_CANCER_HEAD_AND_NECK_VS_CERVICAL_UP | 179 | 7.1759977 | 0 | 0 |
| HALLMARK_MYC_TARGETS_V1 | 199 | 7.1640797 | 0 | 0 |
| BIDUS_METASTASIS_UP | 211 | 7.1628585 | 0 | 0 |
| MITSIADES_RESPONSE_TO_APLIDIN_DN | 245 | 7.151733 | 0 | 0 |
| HORIUCHI_WTAP_TARGETS_DN | 298 | 7.0227513 | 0 | 0 |
| RNA_BINDING | 251 | 6.9520626 | 0 | 0 |
| PUJANA_BRCA_CENTERED_NETWORK | 117 | 6.9415245 | 0 | 0 |
| SOTIRIOU_BREAST_CANCER_GRADE_1_VS_3_UP | 146 | 6.935544 | 0 | 0 |
| ZHANG_TLX_TARGETS_UP | 88 | 6.9355173 | 0 | 0 |
| TOYOTA_TARGETS_OF_MIR34B_AND_MIR34C | 431 | 6.882011 | 0 | 0 |
| CHANG_CYCLING_GENES | 143 | 6.776958 | 0 | 0 |
| ELVIDGE_HYPOXIA_UP | 167 | 6.6682673 | 0 | 0 |
| CHICAS_RB1_TARGETS_GROWING | 235 | 6.6423955 | 0 | 0 |
| ENK_UV_RESPONSE_KERATINOCYTE_DN | 482 | 6.6009755 | 0 | 0 |
| HOFFMANN_LARGE_TO_SMALL_PRE_BII_LYMPHOCYTE_UP | 158 | 6.5946774 | 0 | 0 |
| WANG_RESPONSE_TO_GSK3_INHIBITOR_SB216763_DN | 350 | 6.5912657 | 0 | 0 |
| FUJII_YBX1_TARGETS_DN | 195 | 6.5864577 | 0 | 0 |
| REACTOME_CELL_CYCLE_MITOTIC | 305 | 6.5813546 | 0 | 0 |
| MARKEY_RB1_ACUTE_LOF_DN | 223 | 6.5600424 | 0 | 0 |
| REACTOME_SRP_DEPENDENT_COTRANSLATIONAL_PROTEIN_TARGETING_TO_MEMBRANE | 125 | 6.445501 | 0 | 0 |
| REACTOME_TRANSLATION | 164 | 6.4340806 | 0 | 0 |
| ELVIDGE_HYPOXIA_BY_DMOG_UP | 128 | 6.4263678 | 0 | 0 |
| WHITEFORD_PEDIATRIC_CANCER_MARKERS | 115 | 6.423127 | 0 | 0 |
| VERNELL_RETINOBLASTOMA_PATHWAY_UP | 69 | 6.4157257 | 0 | 0 |
| MISSIAGLIA_REGULATED_BY_METHYLATION_DN | 119 | 6.3404193 | 0 | 0 |
| ZHOU_CELL_CYCLE_GENES_IN_IR_RESPONSE_6HR | 82 | 6.3366714 | 0 | 0 |
| LINDGREN_BLADDER_CANCER_CLUSTER_3_UP | 313 | 6.318196 | 0 | 0 |
| REACTOME_CELL_CYCLE | 394 | 6.318097 | 0 | 0 |
| KEGG_RIBOSOME | 87 | 6.313869 | 0 | 0 |
| WINNEPENNINCKX_MELANOMA_METASTASIS_UP | 157 | 6.3075743 | 0 | 0 |
| BORCZUK_MALIGNANT_MESOTHELIOMA_UP | 298 | 6.2469683 | 0 | 0 |
| MORI_IMMATURE_B_LYMPHOCYTE_DN | 90 | 6.2466288 | 0 | 0 |
| REACTOME_INFLUENZA_LIFE_CYCLE | 151 | 6.245035 | 0 | 0 |
| REACTOME_PEPTIDE_CHAIN_ELONGATION | 101 | 6.220243 | 0 | 0 |
| RICKMAN_TUMOR_DIFFERENTIATED_WELL_VS_POORLY_UP | 227 | 6.2001324 | 0 | 0 |
| JOHNSTONE_PARVB_TARGETS_2_DN | 320 | 6.1616898 | 0 | 0 |
| REACTOME_3_UTR_MEDIATED_TRANSLATIONAL_REGULATION | 122 | 6.129982 | 0 | 0 |
| REACTOME_INFLUENZA_VIRAL_RNA_TRANSCRIPTION_AND_REPLICATION | 117 | 6.115615 | 0 | 0 |
| KONG_E2F3_TARGETS | 94 | 6.0582504 | 0 | 0 |
| DAZARD_RESPONSE_TO_UV_NHEK_DN | 308 | 6.0501842 | 0 | 0 |
| KIM_WT1_TARGETS_DN | 440 | 6.040182 | 0 | 0 |
| ZHOU_CELL_CYCLE_GENES_IN_IR_RESPONSE_24HR | 123 | 6.031068 | 0 | 0 |
| REACTOME_NONSENSE_MEDIATED_DECAY_ENHANCED_BY_THE_EXON_JUNCTION_COMPLEX | 122 | 6.0127916 | 0 | 0 |
| ROME_INSULIN_TARGETS_IN_MUSCLE_UP | 417 | 5.9895835 | 0 | 0 |
| KAUFFMANN_DNA_REPAIR_GENES | 228 | 5.9600086 | 0 | 0 |
| SENGUPTA_NASOPHARYNGEAL_CARCINOMA_UP | 283 | 5.9519424 | 0 | 0 |
| CHIANG_LIVER_CANCER_SUBCLASS_PROLIFERATION_UP | 170 | 5.9290605 | 0 | 0 |
| LI_WILMS_TUMOR_VS_FETAL_KIDNEY_1_DN | 162 | 5.904452 | 0 | 0 |
| CROONQUIST_IL6_DEPRIVATION_DN | 98 | 5.893453 | 0 | 0 |
| LEE_LIVER_CANCER_SURVIVAL_DN | 169 | 5.881713 | 0 | 0 |
| ISHIDA_E2F_TARGETS | 51 | 5.8812814 | 0 | 0 |
| REACTOME_METABOLISM_OF_RNA | 271 | 5.88044 | 0 | 0 |
| ELVIDGE_HIF1A_AND_HIF2A_TARGETS_DN | 102 | 5.863459 | 0 | 0 |
| ORGANELLE_LUMEN | 445 | 5.8496704 | 0 | 0 |
| BENPORATH_ES_1 | 366 | 5.8346386 | 0 | 0 |
| RUIZ_TNC_TARGETS_DN | 137 | 5.815768 | 0 | 0 |
| MARTINEZ_RESPONSE_TO_TRABECTEDIN_DN | 268 | 5.815715 | 0 | 0 |
| REACTOME_DNA_REPLICATION | 184 | 5.8120737 | 0 | 0 |
| REACTOME_MRNA_SPLICING | 107 | 5.8085995 | 0 | 0 |
| BENPORATH_PROLIFERATION | 137 | 5.8034053 | 0 | 0 |
| BURTON_ADIPOGENESIS_3 | 101 | 5.7869525 | 0 | 0 |
| CREIGHTON_ENDOCRINE_THERAPY_RESISTANCE_1 | 500 | 5.7635136 | 0 | 0 |
| MENSE_HYPOXIA_UP | 96 | 5.7520404 | 0 | 0 |
| GRAHAM_CML_DIVIDING_VS_NORMAL_QUIESCENT_UP | 173 | 5.749794 | 0 | 0 |
| MEMBRANE_ENCLOSED_LUMEN | 445 | 5.7389297 | 0 | 0 |
| REACTOME_PROCESSING_OF_CAPPED_INTRON_CONTAINING_PRE_MRNA | 136 | 5.7275925 | 0 | 0 |
| KAUFFMANN_MELANOMA_RELAPSE_UP | 60 | 5.7181473 | 0 | 0 |
| SARRIO_EPITHELIAL_MESENCHYMAL_TRANSITION_UP | 172 | 5.6923094 | 0 | 0 |
| REACTOME_METABOLISM_OF_PROTEINS | 441 | 5.6786656 | 0 | 0 |
| GRESHOCK_CANCER_COPY_NUMBER_UP | 317 | 5.6653214 | 0 | 0 |
| REN_BOUND_BY_E2F | 61 | 5.6499715 | 0 | 0 |
| ELVIDGE_HIF1A_TARGETS_DN | 89 | 5.640759 | 0 | 0 |
| NUCLEOPLASM | 270 | 5.614174 | 0 | 0 |
| STRUCTURAL_CONSTITUENT_OF_RIBOSOME | 79 | 5.5992007 | 0 | 0 |
| TARTE_PLASMA_CELL_VS_PLASMABLAST_DN | 307 | 5.5893526 | 0 | 0 |
| NUCLEAR_LUMEN | 376 | 5.587485 | 0 | 0 |
| REACTOME_MRNA_PROCESSING | 156 | 5.581983 | 0 | 0 |
| KEGG_SPLICEOSOME | 125 | 5.5482755 | 0 | 0 |
| OSMAN_BLADDER_CANCER_UP | 389 | 5.517252 | 0 | 0 |
| SPIELMAN_LYMPHOBLAST_EUROPEAN_VS_ASIAN_UP | 472 | 5.470411 | 0 | 0 |
| CELL_CYCLE_PROCESS | 188 | 5.4666224 | 0 | 0 |
| CROONQUIST_NRAS_SIGNALING_DN | 72 | 5.450184 | 0 | 0 |

NES: normalized enrichment score; FDR: false discovery rate.

**Supplementary Table 9. Genes differentially expressed in CD133^hi^ vs. Notch^hi^ cells (196 genes, P<0.1)**

| **gene ID** | **log2 change** | **fold change** | **P value** |
| --- | --- | --- | --- |
| PROM1 | 2.76396 | 6.792581639 | 0.00000025 |
| NDUFA4L2 | 1.8158 | 3.520547969 | 0.0088232 |
| ACAN | 1.80539 | 3.495236314 | 0.03772652 |
| CA9 | 1.73368 | 3.325750637 | 0.01065589 |
| KRBOX1-AS1 | 1.63462 | 3.105057533 | 0.05711379 |
| FGFBP2 | 1.56939 | 2.967792035 | 0.05025498 |
| PYCARD | 1.54756 | 2.923223222 | 0.07619711 |
| NPTX1 | 1.54557 | 2.919193816 | 0.02911094 |
| RP11-227D13.4 | 1.49376 | 2.816219922 | 0.04814726 |
| RP11-231E19.1 | 1.49259 | 2.813936943 | 0.08765887 |
| LINC00996 | 1.49213 | 2.813039869 | 0.08874121 |
| BMP6 | 1.48277 | 2.794848343 | 0.0251554 |
| RP11-24H1.1 | 1.47447 | 2.778815404 | 0.08694221 |
| LINC00636 | 1.46904 | 2.768376187 | 0.09142606 |
| RNA5SP364 | 1.46651 | 2.763525644 | 0.09326467 |
| RNU6-19P | 1.4629 | 2.756619225 | 0.09444049 |
| IL20RB-AS1 | 1.44986 | 2.731815404 | 0.09211476 |
| ADCY8 | 1.44591 | 2.72434611 | 0.0131579 |
| PMEL | 1.44448 | 2.721647075 | 0.0849206 |
| KCNK3 | 1.44264 | 2.718178124 | 0.08870686 |
| RP11-1167A19.6 | 1.44048 | 2.714111518 | 0.08471613 |
| NPY | 1.43895 | 2.711234688 | 0.01757294 |
| ESPN | 1.43758 | 2.70866129 | 0.08852694 |
| AC005082.12 | 1.41463 | 2.665913554 | 0.06905405 |
| RP11-122A21.2 | 1.38853 | 2.618117779 | 0.05790746 |
| TNMD | 1.38662 | 2.614653917 | 0.09685195 |
| B3GNT7 | 1.38297 | 2.608047237 | 0.07388309 |
| RP11-298E2.2 | 1.33731 | 2.526797413 | 0.08937197 |
| RP11-58K22.4 | 1.33155 | 2.516729216 | 0.09861527 |
| CDH5 | 1.32019 | 2.496989925 | 0.05771547 |
| RNU6-541P | 1.30561 | 2.471882214 | 0.08695638 |
| LOXL2 | 1.26777 | 2.407890857 | 0.04759123 |
| APLN | 1.24734 | 2.374033018 | 0.00324357 |
| MAMDC2 | 1.22691 | 2.340651261 | 0.08151781 |
| STAC2 | 1.21607 | 2.32313018 | 0.01470623 |
| SLC16A3 | 1.20854 | 2.311036427 | 0.00531813 |
| SERPINF1 | 1.19532 | 2.289956193 | 0.03489449 |
| PCSK1 | 1.18627 | 2.275636301 | 0.0043058 |
| KB-1980E6.3 | 1.18289 | 2.270311095 | 0.04786328 |
| RP11-264B14.2 | 1.13873 | 2.201871078 | 0.09062372 |
| BEST3 | 1.13407 | 2.194770363 | 0.07767018 |
| CLDN10 | 1.13356 | 2.193994638 | 0.09794703 |
| C4orf47 | 1.13301 | 2.193158379 | 0.00342336 |
| SNORA28 | 1.11963 | 2.172912379 | 0.09512365 |
| HK2 | 1.08638 | 2.123405641 | 0.02388621 |
| RP11-256I23.3 | 1.06273 | 2.088880556 | 0.08660931 |
| RP11-439C15.4 | 1.05923 | 2.083819042 | 0.0735945 |
| ZNF395 | 1.05152 | 2.072712474 | 0.08005673 |
| TEKT2 | 1.04485 | 2.063151837 | 0.06414699 |
| ESRRB | 1.01691 | 2.023580161 | 0.09614434 |
| RP11-489G11.3 | 1.00613 | 2.008516064 | 0.08466385 |
| RP11-536O18.2 | 0.99566 | 1.993992523 | 0.0782391 |
| SIK1 | 0.99196 | 1.988885193 | 0.08937864 |
| RP11-560J1.2 | 0.98157 | 1.974613095 | 0.09146284 |
| EFR3B | 0.97797 | 1.969691927 | 0.04494608 |
| JPH1 | 0.96586 | 1.953227506 | 0.08638559 |
| HILPDA | 0.94626 | 1.926871014 | 0.0053031 |
| INSIG2 | 0.93883 | 1.916972977 | 0.06794799 |
| DNAH11 | 0.93727 | 1.914901256 | 0.06402863 |
| PDK1 | 0.93559 | 1.912672676 | 0.02124344 |
| ACSBG2 | 0.87955 | 1.839801348 | 0.07948021 |
| TMEM45A | 0.83378 | 1.782349176 | 0.0969685 |
| RP11-554D20.1 | 0.82447 | 1.770884352 | 0.04903137 |
| ITPR1 | 0.81592 | 1.760420406 | 0.01166017 |
| PFKFB4 | 0.81158 | 1.755132563 | 0.07040896 |
| MIR210HG | 0.8111 | 1.754548709 | 0.00934698 |
| MEST | 0.79795 | 1.738628863 | 0.06207968 |
| PDE6A | 0.79769 | 1.738315558 | 0.07072148 |
| SSH3 | 0.7578 | 1.690910149 | 0.08931804 |
| RP11-798M19.6 | 0.75387 | 1.686310262 | 0.04952055 |
| PLOD2 | 0.74868 | 1.680254771 | 0.03250436 |
| HSPB6 | 0.71713 | 1.643908499 | 0.06154991 |
| PPP1R3C | 0.68051 | 1.60270622 | 0.04752934 |
| PGK1 | 0.67174 | 1.59299308 | 0.08600817 |
| FAM162A | 0.66931 | 1.590312185 | 0.00141163 |
| ELL2 | 0.64248 | 1.561010239 | 0.052358 |
| MLLT3 | 0.63033 | 1.547919022 | 0.02506355 |
| P4HA1 | 0.62638 | 1.543686722 | 0.01266826 |
| ENO2 | 0.60467 | 1.520630887 | 0.09156211 |
| RORA | 0.59129 | 1.506593281 | 0.08956001 |
| LLNLR-246C6.1 | 0.57325 | 1.487871569 | 0.09711016 |
| TRIM2 | 0.56307 | 1.477409746 | 0.07974296 |
| DUSP5 | 0.53122 | 1.445150756 | 0.06664767 |
| RPS20P22 | 0.51499 | 1.428984235 | 0.0613274 |
| GBE1 | 0.51198 | 1.426005949 | 0.05285571 |
| UCP2 | 0.50875 | 1.422816884 | 0.06198932 |
| SAP30 | 0.49018 | 1.404620114 | 0.0592823 |
| GPI | 0.44111 | 1.357648491 | 0.0973303 |
| LBR | 0.33146 | 1.25828611 | 0.08624119 |
| KBTBD8 | -0.49452 | 0.709797796 | 0.09187928 |
| LINC01004 | -0.54669 | 0.684588992 | 0.09348456 |
| FGFR3 | -0.6254 | 0.648240022 | 0.04563936 |
| RN7SL832P | -0.69472 | 0.617829216 | 0.06809446 |
| AC147651.3 | -0.73283 | 0.601722413 | 0.06328874 |
| FRMPD1 | -0.75695 | 0.591746019 | 0.07690611 |
| HSD17B3 | -0.78126 | 0.581858396 | 0.07743587 |
| HIST1H2BJ | -0.82239 | 0.565504339 | 0.0778608 |
| PRR22 | -0.82248 | 0.565469063 | 0.03751248 |
| APOL4 | -0.88062 | 0.543133969 | 0.06829 |
| CPB1 | -0.89256 | 0.538657445 | 0.07311651 |
| ATP2B2 | -0.90509 | 0.53399939 | 0.04817604 |
| RP11-416O18.1 | -0.90816 | 0.532864268 | 0.05738482 |
| B3GALT5 | -0.91519 | 0.53027403 | 0.08379252 |
| AOC2 | -0.91866 | 0.529000137 | 0.03129507 |
| MIR29A | -0.95595 | 0.515502025 | 0.07050197 |
| TTC18 | -0.95687 | 0.515173397 | 0.06724281 |
| ART3 | -0.98345 | 0.505768818 | 0.07872503 |
| RP11-506H20.1 | -1.00183 | 0.499366172 | 0.04313496 |
| ERMN | -1.00213 | 0.499262343 | 0.09591379 |
| RP11-577N1.1 | -1.041 | 0.485990494 | 0.09309538 |
| RP6-99M1.2 | -1.05789 | 0.480334054 | 0.09023576 |
| CTD-2287O16.4 | -1.06546 | 0.477820283 | 0.09958784 |
| ALDH1A2 | -1.06646 | 0.477489198 | 0.06546924 |
| CACNA2D2 | -1.07162 | 0.475784441 | 0.05409706 |
| SLC22A4 | -1.09187 | 0.469152871 | 0.00598658 |
| ACSBG1 | -1.11456 | 0.461831986 | 0.08391259 |
| GRIK3 | -1.12433 | 0.458715003 | 0.01538641 |
| RN7SL200P | -1.15592 | 0.44877991 | 0.06777706 |
| KIAA1683 | -1.16601 | 0.445652159 | 0.09509602 |
| ACOT12 | -1.1791 | 0.441626913 | 0.02247568 |
| DHRS2 | -1.18332 | 0.440337006 | 0.07950235 |
| RP11-661A12.12 | -1.18832 | 0.438813556 | 0.08927962 |
| LAPTM5 | -1.19168 | 0.43779276 | 0.09777474 |
| HCLS1 | -1.19348 | 0.437246882 | 0.08918292 |
| HIST1H2AG | -1.2207 | 0.429074479 | 0.02561179 |
| RP11-385J1.2 | -1.22338 | 0.428278156 | 0.04090642 |
| CTD-2072I24.1 | -1.22796 | 0.426920694 | 0.07487725 |
| ENTPD2 | -1.23087 | 0.426060438 | 0.09951224 |
| RN7SL592P | -1.23168 | 0.425821294 | 0.07636879 |
| RP11-290H9.4 | -1.23448 | 0.424995656 | 0.09977457 |
| TPTE2 | -1.26029 | 0.417460037 | 0.04232507 |
| RP11-49C9.2 | -1.26916 | 0.414901276 | 0.04154351 |
| PON1 | -1.28681 | 0.409856278 | 0.07089855 |
| CPED1 | -1.29539 | 0.407426013 | 0.04805181 |
| RNU2-17P | -1.30431 | 0.404914721 | 0.08896293 |
| HIST1H2BC | -1.32029 | 0.400454434 | 0.06325709 |
| RP11-649A18.5 | -1.3214 | 0.400146446 | 0.04872187 |
| RP3-400B16.1 | -1.32505 | 0.39913536 | 0.07208134 |
| LAMC2 | -1.32962 | 0.397873026 | 0.07430423 |
| HIST1H4E | -1.33518 | 0.396342615 | 0.09315785 |
| APOD | -1.33539 | 0.396284927 | 0.04559239 |
| RPE65 | -1.34257 | 0.394317597 | 0.03211933 |
| SLCO2A1 | -1.34287 | 0.39423561 | 0.08638586 |
| RP11-133K1.6 | -1.34457 | 0.393771336 | 0.08756226 |
| AC013439.4 | -1.34786 | 0.392874381 | 0.08978915 |
| PAWR | -1.35916 | 0.389809188 | 0.09648852 |
| KRT18P62 | -1.36964 | 0.386987803 | 0.09973925 |
| G6PC2 | -1.37023 | 0.386829574 | 0.09725216 |
| FAM189A2 | -1.39126 | 0.381231702 | 0.00329635 |
| CD36 | -1.41593 | 0.374768084 | 0.09988771 |
| RP1-155D22.1 | -1.42051 | 0.373580226 | 0.09396627 |
| SLC1A7 | -1.42103 | 0.373445599 | 0.08734353 |
| AJ239322.1 | -1.42103 | 0.373445599 | 0.0937741 |
| AC009542.2 | -1.43165 | 0.370706676 | 0.09862429 |
| FOXE3 | -1.43959 | 0.368672062 | 0.08096001 |
| RP4-609E1.2 | -1.45208 | 0.365494094 | 0.09078338 |
| RP11-571L19.7 | -1.45385 | 0.365045955 | 0.09109176 |
| GAPDHP55 | -1.45488 | 0.364785427 | 0.03339502 |
| LINC00520 | -1.45887 | 0.363777949 | 0.09529081 |
| RP11-321E8.5 | -1.46168 | 0.363070093 | 0.09543209 |
| RP11-503I22.2 | -1.46288 | 0.362768225 | 0.07376001 |
| SCN1A | -1.46645 | 0.361871652 | 0.01025446 |
| TMEM253 | -1.46693 | 0.361751273 | 0.01799411 |
| LRRTM1 | -1.47136 | 0.360642168 | 0.0237838 |
| CARD6 | -1.47211 | 0.360454734 | 0.09269756 |
| GABRP | -1.47498 | 0.359738382 | 0.08289039 |
| SNRK-AS1 | -1.47568 | 0.359563878 | 0.09119939 |
| CYP19A1 | -1.47943 | 0.358630477 | 0.04979652 |
| RAD17P1 | -1.47965 | 0.358575792 | 0.08853259 |
| RP11-178C3.2 | -1.47995 | 0.358501236 | 0.08737905 |
| RP1-34B20.4 | -1.48777 | 0.356563269 | 0.03128895 |
| C7orf34 | -1.4878 | 0.356555855 | 0.07298582 |
| XXbac-B444P24.14 | -1.49645 | 0.354424441 | 0.08738523 |
| HIST1H2BF | -1.51102 | 0.350863067 | 0.05884574 |
| TPH2 | -1.52236 | 0.348115993 | 0.06583285 |
| RP11-524F11.1 | -1.52318 | 0.347918187 | 0.06519829 |
| MYOT | -1.52746 | 0.346887558 | 0.07893736 |
| RP11-213G2.5 | -1.53824 | 0.34430523 | 0.04499741 |
| RP11-464I4.1 | -1.53993 | 0.34390214 | 0.06830747 |
| RP11-97N5.2 | -1.57415 | 0.335840937 | 0.01478213 |
| IL6 | -1.58502 | 0.333320048 | 0.07001024 |
| ABCA4 | -1.59892 | 0.330124016 | 0.06741441 |
| RBMXP4 | -1.59973 | 0.32993872 | 0.06195622 |
| HIST1H4D | -1.60654 | 0.328384969 | 0.0420685 |
| SEMA3D | -1.63054 | 0.322967299 | 0.06157846 |
| RP11-133O22.6 | -1.64138 | 0.320549708 | 0.06114531 |
| RP11-76E16.2 | -1.65528 | 0.317476124 | 0.04944526 |
| RP4-622L5.7 | -1.67967 | 0.312154031 | 0.05520287 |
| LPAL2 | -1.70508 | 0.306704237 | 0.02870321 |
| GLULP3 | -1.72257 | 0.303008464 | 0.04790144 |
| RP11-295P9.12 | -1.72832 | 0.301803198 | 0.03145773 |
| CTD-2547L16.3 | -1.81785 | 0.283643361 | 0.03513883 |
| RP11-158G18.1 | -1.8194 | 0.283338784 | 0.0376842 |
| IL32 | -1.87065 | 0.273450196 | 0.02938481 |
| ABCB1 | -2.06718 | 0.238625479 | 0.01803522 |
| DSPP | -2.49202 | 0.177757212 | 0.00334141 |

**Supplementary Table 10. Top 10 (a) GSEA gene sets and (b) DAVID GO terms enriched in genes differentially expressed between CD133^hi^ *vs.* Notch^hi^ cells.**

**(a)**

| **gene set** | **size** | **NES** | **FDR** | **Nominal P value** |
| --- | --- | --- | --- | --- |
| MENSE_HYPOXIA_UP | 15 | 2.5071154 | 0 | 0 |
| KRIEG_HYPOXIA_NOT_VIA_KDM3A | 17 | 2.3654783 | 0.0012982456 | 0 |
| ELVIDGE_HYPOXIA_UP | 13 | 2.2615654 | 0.0024714237 | 0 |
| ELVIDGE_HIF1A_AND_HIF2A_TARGETS_DN | 13 | 2.2201686 | 0.0022821391 | 0 |
| MARTORIATI_MDM4_TARGETS_FETAL_LIVER_UP | 10 | 2.2100518 | 0.0023983428 | 0 |
| ELVIDGE_HYPOXIA_BY_DMOG_UP | 13 | 2.198943 | 0.0024692074 | 0 |
| ELVIDGE_HIF1A_TARGETS_DN | 12 | 2.1023552 | 0.0044012475 | 0 |
| QI_HYPOXIA | 11 | 2.0956895 | 0.0038510915 | 0 |
| NAKAMURA_TUMOR_ZONE_PERIPHERAL_VS_CENTRAL_DN | 20 | 1.9948369 | 0.01000314 | 0 |
| BASAKI_YBX1_TARGETS_DN | 12 | 1.9654329 | 0.011677007 | 0.0085227275 |
| WINTER_HYPOXIA_METAGENE | 11 | 1.6340482 | 0.07854654 | 0.036745407 |

NES: normalized enrichment score; FDR: false discovery rate.

**(b)**

| **GO Term** | **P value** | **Genes** | **Fold Enrichment** |
| --- | --- | --- | --- |
| GO:0061621~canonical glycolysis | 1.57E-06 | GPI, PFKFB4, ENO2, HK2, PGK1 | 56.63630229 |
| Glycolysis | 1.00E-04 | GPI, ENO2, HK2, PGK1 | 43.50713908 |
| GO:0006096~glycolytic process | 1.96E-04 | GPI, ENO2, HK2, PGK1 | 34.64809082 |
| Cleavage on pair of basic residues | 0.00171 | PCSK1, NPY, PMEL, APLN, CDH5, BMP6 | 6.834736376 |
| Carbohydrate transport and metabolism | 0.00201 | TRIM2, GBE1, HK2 | 31.98947368 |
| hsa00010:Glycolysis / Gluconeogenesis | 0.00323 | GPI, ENO2, HK2, PGK1 | 12.89179104 |
| Disease mutation | 0.00530 | DNAH11, ESRRB, ITPR1, KCNK3, ESPN, PROM1, PDE6A, GPI, PCSK1, TRIM2, GBE1, PLOD2, ACAN, SIK1, PGK1, LBR | 2.124807107 |
| GO:0030199~collagen fibril organization | 0.00748 | P4HA1, ACAN, LOXL2 | 22.65452092 |
| hsa00500:Starch and sucrose metabolism | 0.00943 | GPI, GBE1, HK2 | 19.63068182 |
| GO:0006094~gluconeogenesis | 0.00945 | GPI, ENO2, PGK1 | 20.08014354 |

**Supplemental Table 11. Taqman Gene expression assays (a) and primers (b) used in the study.**

| **a** |  |
| --- | --- |
| **gene ID** | **Taqman Gene Expression Probe ID** |
| *PROM1* | Hs00195682_m1 |
| *NOTCH1* | Hs01062014_m1 |
| *NOTCH2* | Hs01050702_m1 |
| *HES1* | Hs00172878_m1 |
| *HES5* | Hs01387463_g1 |
| *HEY1* | Hs01114113_m1 |
| *DLL1* | Hs00194509_m1 |
| *JAG1* | Hs01970032_m1 |
| *JAG2* | Hs00171432_m1 |
| *HPRT1* | Hs02800695_m1 |
|  |  |
| **b** |  |
| **Primer name** | **Sequence** |
| copGFP-Forward | 5’-CTTCCTGCACGCCATCAA-3’ |
| copGFP-Reverse | 5’-TTGAAGTCGCCGATCACG-3’ |

**Supplementary Table 12. Antibodies used in the study.**

| Target | Catalog No | Company | Dilution |
| --- | --- | --- | --- |
| NICD1 | ab8925 | Abcam | 1:100 |
| CD133/1 (W6B3C1) | 130-092-395 | Miltenyi | 1:50 |
| DsRed | 632496 | Clontech | 1:500 |
| copGFP | AB513 | Evrogen | 1:500 |
| GFP | ab13970 | Abcam | 1:500 |
| CD105 | AF1320 | R&D Systems | 1:200 |
| Human nuclei (hNA) | MAB1281 | Milipore | 1:200 |
| HIF1α | A300-286A | Bethyl Labs | 1:100 |
| α-SMA | ab5694 | Abcam | 1:100 |
| CAIX | ab15086 | Abcam | 1:200 |
| Ki67 | ab15580 | Abcam | 1:200 |
